
JOURNAL INFORMATION
==============================
NLM Title Abbreviation: J Orthop Traumatol
Journal ID: J Orthop Traumatol
NLM Journal ID: 101090931

Journal of Orthopaedics and Traumatology : Official Journal of the Italian Society of Orthopaedics and Traumatology

ISSN: 1590-9921
EISSN: 1590-9999
Publisher: Springer

ARTICLE INFORMATION
==============================
PMCID: PMC4417749
DOI: 10.1007/s10195-011-0151-1
Article ID: 151
Article version: 1
Subjects: Events

In-Depth Oral Presentations, Oral Communications and Round Table

Electronic publication date: 2011 Sep 2
Print publication date: 2011 Oct
Volume: 12
Issue: Suppl 1
First page: 125
Last page: 164
Copyright: © The Author(s) 2011
License: Open AccessThis article is distributed under the terms of the Creative Commons Attribution 2.0 International License (https://creativecommons.org/licenses/by/2.0), which permits unrestricted use, distribution, and reproduction in any medium, provided the original work is properly cited.
License URL: https://creativecommons.org/licenses/by/2.0/

Keywords: Total Knee Arthroplasty, Hyaluronic Acid, UHMWPE, Slip Capital Femoral Epiphysis, Demineralized Bone Matrix
issue-copyright-statement: © The Author(s) 2011

==============================
J Orthopaed Traumatol (2011) 12 (Suppl 1):S125–S164
DOI 10.1007/s10195-011-0151-1

5 OCTOBER 2011

### In-Depth Oral Presentations, Oral Communications and Round Table

### IN-DEPTH ORAL PRESENTATIONS

### *AT13 – BIOMATERIALS AND COATINGS IN ORTHOPAEDICS 1*

### Nerve repair in developmental age: synthetic tubulization versus nerve grafting

F.M. Senes*, N. Catena

IRCCS Istituto Giannina Gaslini (Genova, IT)

**Introduction** Traumatic nerve injuries are common occurrences in developmental age, often due to blunt injury, sometimes to open lesion. In the child the small size of the nerves, the need to find appropriate nervous tissue to repair of such injuries and the aesthetic damage to the area of donor site for nerve graft have incentivated the study of alternative methods (tubulization) instead of classical nerve grafting. Moreover case series of nerve injuries in children are few in number and few are the report of nerve repair by means of synthetic tubulization. Our group applied synthetic tubulization in children since the year 2005, therefore the comparison of two homogeneous groups of nerve repair appeared to be a validation of the methods.
**Materials and methods** We considered 20 patients, affected by brachial plexus or peripheral nerve injuries of upper and lower limb and treated in the period from January 2005 to December 2009. Ten patients of them underwent nerve repair by means of synthetic tubulization, while the others had nerve graft repair.
**Results** Evaluation of results confirms how difficult is to compare nerve injuries, especially when nerve damage is related to large trunks, such as brachial plexus. Nerve guides in peripheral injuries repair, and particularly in brachial plexus surgery, showed slower clinical and electrophysiological signs of recovery compared to nerve grafting, but similar at a long distance in time. Failure turned out to be possible in both the methods.
**Discussion** Regardless of the outcome in itself, the peculiarity of the paper is to specify the role of nerve repair in children by means of synthetic tubulization.
**Conclusions** Nerve tubulization can be considered during repair of nerve lesions in developmental age as a technical advance in the presence of large nerve gaps when conventional nerve graft is insufficient, or even can represent an alternative to nerve graft. In small children, it is sometimes difficult to find veins with appropriate diameter to create biological nerve guides. In these cases, it is mandatory to use synthetic reabsorbable guides and thus spare autologous structures and avoid morbidity of donor site. The only disadvantage of this approach is the high cost of the tube and the low adaptability of synthetic guides compared to autologous nerve grafts.

### Ion levels in trabecular and conventional titanium cups

A. Bistolfi*[1], R. Lo Baido[1], A. Cimino[1], D. Sorasio[1], A. Massè[2], G. Maina[1], G. Massazza[1], M. Crova[1]

[1]Ospedale CTO/M. Adelaide, Dipartimento di Ortopedia, Traumatologia e Medicina del Lavoro (Turin, IT);
[2]Università degli Studi di Torino, Ospedale S. Luigi (Turin, IT)

**Introduction** Osteo-integration of non-cemented acetabular component in hip prostheses is related to several factors, like the roughness of the surface. A new type of extremely porous biomaterial for the acetabular cups, the Trabecular titanium (Delta TT, Lima), was recently developed. It has a macroscopic surface which is similar to that of the cancellous bone. It is also biocompatible and it has good mechanical properties. This material, when compared to full titanium conventional cups (Delta, Lima), has a surface hundreds times wider due to the 3-dimensional morphology. Aim of this study is to evaluate the urinary and ematic level of metal ions released from Trabecular titanium acetabular cups (D-TT) compared to conventional full titanium cups (D).
**Materials and methods** Twenty patients entered the study: 10 subjects (average age, 70 years) were implanted with the Delta (D) cup and 10 subjects (average age, 64 years) were implanted with the Delta-TT (D-TT) cup. Aluminum (Al), Vanadium (V) and Titanium (Ti) in urine and in blood were assessed before surgery (T0) and then after one week (T1), 3 months (T2) and 6 months (T3). An ICP/MS Inductively-coupled plasma mass spectroscopy Agilent 7500 was used for the analysis of the samples (calibration solutions prepared with AccuTrace multielement certified standard, referred to a primary standard solution NIST), analytical range of 0–100 μg/l and the 5 point calibration curve. Statistical analysis between the two groups and the variance within each group were studied.
**Results** The two groups were homogeneous for patients characteristics. No differences were detected at T0 and T1 between the patients and the values in the population. Also, no significant differences were observed on blood and urine between the two groups at different times, as well as within the same group at different times. Only the concentration of vanadium in urine at T1 was higher than in group D-TT.
**Discussion** Although the studied metals are not reported as toxic in humans at the concentrations detected after total joint replacement, ion concentration in tissues is always a concerning topic. The starting hypothesis was that a much larger surface of the new Trabecular titanium would determine a higher release of metal ions.
**Conclusions** These results show no differences from conventional and Trabecular cups.

### Cementless acetabular fixation using modular porous tantalum implants after radiation therapy for pelvic malignancies

N. Fabbri*, C. Errani, M. Mercuri

Istituto Ortopedico Rizzoli (Bologna, IT)

**Introduction** Durable acetabular fixation is difficult to obtain after radiotherapy for pelvic malignancy. Acrylic cement is a short-term satisfactory solution but is associated with significant tendency to fail at a follow-up longer than 3 years. Porous tantalum has been very successfully used during the last decade in joint revision surgery and its great potential for osseointegration makes it an ideal material for use in biomechanically difficult situations such as radiotherapy sequelae. Goal of this study is the analysis of results obtained using a porous tantalum cementless acetabular component in patients undergoing total hip replacement after radiation therapy.
**Materials and methods** This is a retrospective study of 6 patients after primary (4 cases) or revision surgery of previous failure (2 cases) after radiation therapy; 4 females and 2 males; average age, 49 years (26–76 years). Diagnosis related to radiation therapy was a primary musculoskeletal neoplasm of the pelvis in 4 cases and hematologic/secondary condition in 2 cases. At the time of surgery, none of the patients presented evidence of clinical activity of the underlying disease at a minimum follow-up of 2 years (2–12 years). The 2 revision cases consisted of an aseptic loosening and a deep infection, at 3 years and 1 year respectively from previous implant. Deep infection was treated by staged revision. In 2 cases, the porous tantalum acetabular component was associated with an "augment" to fill a segmental periacetabular defect and support the cup. Minimum follow-up after placement of the porous tantalum socket was 2 years. In all cases, mean follow-up was 3 years (2–5.5 years).
**Results** In none of the cases there was local recurrence. There was 1 case of recurrent hip dislocation requiring further surgical management. There was neither clinical nor radiographic evidence of acetabular or femoral loosening at the most recent follow-up. Implant was well functioning and stable in all cases.
**Discussion** These results are very promising to obtain durable acetabular fixation after radiotherapy and are related to the great potential for osseointegration of porous tantalum.
**Conclusions** Porous tantalum has been very satisfactory at short term follow-up, demonstrating reliable capability to obtain biologic fixation after radiation therapy. A longer follow-up is necessary to identify later potential failures.

### Human adipose stem cells can differentiate in an osteoblastic-like phenotype on trabecular titanium scaffolds without osteogenic medium

M. Marullo*[1], M. Scaffino[1], L. Botta[2], G. Gastaldi[3], F. Benazzo[4]

[1]Clinica Ortopedica e Traumatologica, IRCCS Policlinico S. Matteo (Pavia, IT);
[2]Dipartimento di Fisiologia, Università di Pavia (Pavia, IT);
[3]Dipartimento di Fisiologia, Università di Pavia e Centro di Ingegneria Tissutale (Pavia, IT);
[4]Clinica Ortopedica e Traumatologica, IRCCS Policlinico S. Matteo e Centro di Ingegneria Tissutale (Pavia, IT)

**Introduction** Pluripotent human adipose tissue-derived stem cells (hASCs) can differentiate into multiple mesenchymal cell lineages. Previous studies demonstrated that the material, the geometry and the porosity of scaffolds affect cell binding and migration depth of cellular ingrowth, and that hASCs can differentiate in an osteoblastic-like phenotype when seeded on trabecular titanium scaffolds with osteogenic medium. Aim of this study was to demonstrate that trabecular titanium scaffolds could induce osteogenic differentiation of hASCs also without osteogenic medium.
**Materials and methods** hASCs were isolated from subcutaneous adipose tissue, seeded on monolayer and on trabecular titanium scaffolds (Ti6Al 4 V, LimaCorporate, Villanova di San Daniele del Friuli, Italy; average porosity, 65%, average diameter of cell pores, 640 μm) and incubated at 37° C in 5% CO(2) with osteogenic and proliferation medium in static conditions. Cells before seeding corresponded to reference group. After seeding on scaffolds, hASCs were cultured for 7 days in the presence of proliferation medium before the osteogenic medium was added (Time 0). The osteogenic differentiation was detected with real-time PCR to analyze the expression of ALP and RUNX-2 genes, at time 0 and after 3, 7 and 21 days from differentiation. Alkaline phosphatase activity on cell lysates was also measured after 7 days from differentiation.
**Results** *Monolayer*: both ALP and RUNX-2 gene expression and alkaline phosphatase activity were higher in hASCs grown in presence of osteogenic medium.
*Trabecular titanium scaffolds*: in hASCs grown in proliferation medium, ALP gene expression was higher than reference group still at time 0, and remained 2.5 fold higher at time 3 and 7. Similar results were found with osteogenic medium. RUNX-2 gene expression was higher in hASCs grown in osteogenic medium, and increased also in the presence of proliferation medium respect to reference group. At time 7, there were no difference in alkaline phosphatase activity of hASCs grown with proliferation and osteogenic medium.
**Conclusions** hASCs on trabecular titanium expressed higher amount of ALP and RUNX-2 gene expression in proliferation medium with respect to cells on monolayer in the same condition. The expression of osteogenic genes similar in cells grown on scaffold with both medium, leads to the conclusion that trabecular titanium scaffold seems to have not only osteoconductive but also osteoinductive properties.

### War against polyethylene oxidation – lipoic acid – is it an alternative to vitamin E?

F. Conteduca*, F. D'Angelo, R. Iorio, J. Conteduca, L. Caperna, A. Ferretti

Ospedale S. Andrea (Rome, IT)

**Introduction** Failure of prosthetic implants is frequently reported in literature and is related to wear and mechanical failure of ultra high molecular weight polyethylene (UHMWPE). These phenomena are strictly connected to aging and oxidation of plastic material, because irradiation with gamma ray produces OH radicals. A blended alpha-tocopherol UHMWPE is a valid alternative to limit this dangerous aging effect on prosthetic plastic material.
**Materials and methods** The present study takes into consideration a new substance to dope irradiated UHMWPE. Lipoic Acid (LA) was mixed with UHMWPE powder (GUR 1050) and solid pieces were made with 0,1%, 0,3%, 0,5%, 1%, 2% lipoic acid. Solid polyethylene was gamma ray irradiated with 30 kGy (Isomedix, Northborough, MA). An owen (80° Celsius) was used to produce an aging effect for 30 days in the doped and control samples (Conventional not doped polyethylene). This process simulates an aging effect of 10 years into the body. A Furier Transfer Infra Red (FTIR) was used to study oxidation of blended and not blended polyethylene on the surface and 1 mm under the surface. Specifically 1 mm under the surface is the most responsible layer of delamination in prosthetic material.

**Results** Control FTIR shows in the doped lipoic acid samples a lower oxidation index *versus* not doped polyethylene samples. This effect was also obtained in 0.1 concentration of lipoic acid samples.

**Discussion** This knowledge demonstrate that doped UHMWPE could be a valid alternative for the future. Lipoic acid is an antioxidant powder physiologically produced from our body and biologically known for this reason. Lipoic acid can be easily added to polyethylene because is a powder and it is simultaneously hydrosoluble and liposoluble. A low cost can be taken into consideration according to a very low concentration effect.

**Conclusions** This paper demonstrate the possibility of lipoic acid to reduce oxidant effect on aged gamma irradiated polyethylene like alpha tocopherol.

### Metal hypersensitivity in patients with total knee arthroplasties

D. Tigani*[1], D. Granchi[2], E. Cenni[2], N. Baldini[2]

[1]Divisione di Ortopedia Ospedaliera, Policlinico Le Scotte (Siena, IT); [2]Laboratorio di Patofisiologia degli Impianti Ortopedici, Istituti Ortopedici Rizzoli (Bologna, IT)

**Introduction** TKA failure is generally multifactorial. Chronic inflammation following the generation of wear particles has been recognized as the main biological mechanism leading to implant failure which may be induced, including a delayed-type hypersensitivity reaction (DTH). The main goals of our study were to evaluate (a) the frequency of skin sensitization in patients who had undergone TKA, and (b) the clinical impact of this event on the outcome of the implant. For this purpose we chose to use patch testing by applying a panel of haptens representative of cobalt-based alloys (CoCrMo), titanium-based alloys (TiAlV), and bone cements.

**Materials and methods** Ninety-four Caucasian individuals were enrolled in this retrospective case control study. Three groups of patients were evaluated: the first group (no Implant) included 20 patients who were candidates for TKA; the second group consisted of 27 individuals who had already undergone TKA that was clinically and radiographically stable (stable TKA); the third group consisted of 47 patients who had already undergone total knee arthroplasty but showed clinical and radiographic evidence of failure (failed TKA).

**Results** The frequency of positive skin reactions to at least one hapten was significantly higher in group of patients tested after TKA and no differences were observed between stable and loosened implants. In 'No Implant' group, the frequency of positive patch testing for at least one metal hapten was 15%, and differed significantly from that observed in patients with Stable TKA. Patients with stable CoCrMo/TiAlV implants showed a higher percentage of positive patch testing to vanadium in comparison to individuals of the pre-implantation group.

**Discussion** This study confirms that in patients with a TKA the frequency of positive patch testing is higher than in the normal population, although no predictive value is attributable to the sensitization because patch testing was not able to discriminate between stable and loose implants. On the contrary, the presence of symptoms of metal allergy before implantation should be taken into account as a potential risk factor for TKA failure. Ceramics therefore may be a solution for patients with allergies to implant materials and may have an improved resistance to wear, followed by reduced aseptic loosening rates Since the biologic effect of wear particles depends on the material and volume of wear particles, materials with less wear are of scientific interest.

### *AT14 – BIOMATERIALS AND COATINGS IN ORTHOPAEDICS 2*

### Biomolecular coatings on bone implant devices: in vitro and in vivo results on collagen I nanolayers

M. Morra*, C. Cassinelli, G. Cascardo, D. Bollati

Nobil Bio Ricerche Srl (Portacomaro-AT, IT)

**Introduction** Surface modification of implant devices by molecules that can stimulate bone regeneration is a hot-topic in orthopaedic materials coating. Type I collagen is a key-molecule in osteogenesis. The aim of this work is to present our in vitro and in vivo results on collagen I nanolayers coating of bone implant devices.

**Materials and methods** In vitro tests were performed on Ti disks, both coated by porcine collagen I and uncoated control. The process involves chemical bonding to prevent attack by collagenase in the implant site. Surface characterization confirms that collagen I fibrils, a few tens of nanometers thick and a few hundreds of nanometers long, homogeneously cover the treated surface. Adhesion and growth of human mesenchymal cells (HMC) were evaluated, together with gene expression by RT–PCR. In vivo tests were performed by implanting coated and uncoated titanium screws in rabbit (both trabecular and cortical bone), followed by istomorphometry at 4 weeks. Statistical significance of differences between data was checked by Wilcoxon signed rank test.

**Results** Adhesion and growth of HMC on titanium is affected by the collagen nanolayer. The number of adhering cells is greater on the coated samples at every experimental time ($p < 0.05$, at 4, 12, 24 and 96 h). At longer time (2–3 weeks) cells grown on collagen coated surfaces display a different morphology as compared to control, and gene expression by RT–PCR shows overexpression of pro-osteogenic genes, in particular alkaline phosphatases. As to in vivo tests, both bone-to-implant contact and bone ingrowth are significantly greater on collagen coated samples. Differences are more pronounced in trabecular bone.

**Discussion** This work is an example of the ongoing evolution of implant devices from mechanics to biology. Collagen I coating offer to HMC a competitive advantage in the race for the surface of the implanted devices, enhancing adhesion and promoting differentiation towards the osteogenic pathway. This specific bioactivity is reflected in in vivo results, that show faster and more pronounced bone regeneration at the implant surface. From a clinical point of view, main advantages of surface biomolecular stimulation are expected in marrow-rich trabecular bone, such as vertebral bodies, while we are starting evaluation on pathologic bone (osteoporotic, diabetic patients).

**Conclusions** Nanometer thin, collagenase resistant, porcine collagen I coating on implant devices promotes HMC adhesion and differentiation in vitro and stimulates faster peri-implant bone regeneration in vivo.

### A new metal part finishing method for polyethylene wear reduction in total knee prostheses

A. Gigante[1], V. Cigna*[1], S. Manzotti[1], T. Villa[2], M. Mattioli-Belmonte[3]

[1]Dipartimento di Patologia Molecolare e Terapie Innovative, Clinica di Ortopedia e Traumatologia, Facoltà di Medicina e Chirurgia, Università Politecnica delle Marche (Ancona, IT);
[2]Dipartimento di Ingegneria Strutturale, Politecnico di Milano e IRCCS Istituto Ortopedico Galeazzi (Milan, IT);
[3]Dipartimento di Patologia Molecolare e Terapie Innovative, Sezione di Istologia ed Embriologia, Facoltà di Medicina e Chirurgia, Università Politecnica delle Marche (Ancona, IT)

**Introduction** Wear of ultrahigh molecular-weight polyethylene (UHMWPE), with generation of submicrometer- and micrometer-sized particles, has been associated with osteolysis and artificial joint failure. Wear extent is influenced by several factors, some of which are related to manufacturing. Microloy® is a new metal component finishing that involves surface grinding with high-precision multiaxial CNC grinders and an electrochemical mirror-polishing. This method reduces roughness to < 0.03 μm Ra, as well as shape errors, i.e. deviation from the theoretical surfaces, to a few microns.The purpose of this work was thus to determine the effect of Microloy® finishing on UHMWPE wear in pre-implant mobile-bearing knee prostheses.
**Materials and methods** Comparative (traditional *vs.* Microloy® finishing) wear simulation tests were performed (500,000 cycles pin on disk tests and 1,500,000 cycles four degrees of freedom knee simulator tests using GKS Prime® (Permedica S.p.A). Wear was evaluated by gravimetric analysis. Wear debris isolation was performed digesting the serum with proteinase K and vacuum filtering the obtained solution. The filters were scanned at SEM.
**Results** POD test showed 4,87% less wear in the Microloy®-finished pins ($p = 0{,}0439$) in comparison with traditional finishing. In the tests on the knee simulator the Microloy® finished prosthesis showed 48.5% less wear compared with the device with traditional finishing ($p = 0.0020$). A significantly ($p < 0.05$) smaller proportion of the filter was occupied by UHMWPE debris from the Microloy® finished compared with the traditionally finished components, especially the 1 μm pore filters (collecting 10 μm–1 μm debris) ($p = 0.001$).
**Conclusions** Microloy® metal finishing method significantly reduces knee prosthesis UHMWPE wear and debris, particularly those particles implicated in osteolysis. This can lead to clinical advantages in terms of enhanced prosthesis survival.

### In vitro evaluation of a phosphate-based multifunctional bone substitute

D. Bollati*, M. Morra, C. Cassinelli, G. Cascardo

Nobil Bio Ricerche (Portacomaro-AT, IT)

**Introduction** Synthetic bone substitutes based on hydroxyapatite and/or tricalcium phosphate play basically a mechanical role in regeneration of bone defects. There is presently a significant research effort aimed at endowing these products with multifunctional properties, such as molecular stimulation of bone regeneration and antibacterial effects. In this communication we present our results on in vitro characterization of a granular synthetic bone substitute, endowed with a biomolecular coating based on complexes among collagen, hyaluronan and vancomycin. The aim of the work is to confirm the effect on cell stimulation and to evaluate drug-release and antibacterial properties.
**Materials and methods** Granules (diam., max 2 mm–min 0.3 mm) made by 75% beta-tricalciumphosphate and 25% hydroxyapatite were coated with hyaluronan-collagen-vancomycin complex. The process provides a biomolecular coating a few hundreds nanometers thick. The obtained product was analyzed by infrared spectroscopy and HPLC, and used as a substrate for inflammatory cells (J774-A2 macrophages) and human mesenchymal cells (HMC) culturing. RT–PCR measurements were performed at different time interval. The antibacterial effect was evaluated through zone of inhibition test in Agar germ, seeded with *Staphylococcus epidermidis.*
**Results** HPLC measurement shows that about 100 micrograms vancomycin are loaded for every 100 milligrams of phosphate ceramic. Vancomycin release in physiological buffer occurs for a few weeks, about 70% is released in the first week. Zone of inhibition test confirms the antibacterial properties of the product, preventing the growth of *S. epidermidis* (zone of inhibition about 1 cm in the adopted conditions). RT–PCR measurements confirm the effect on J774-A2 cells, decreasing the pro-inflammatory response (in particular IL-1, IL-6 e MCP-1) and upregulating pro-osteogenic transcripts in HMC.
**Discussion** Most of the present-day research effort in implant devices aims at producing multifunctional surfaces, that combine different stimulatory effects on host tissues. The material described adds to the mechanical properties of the synthetic device the biomolecular (collagen/hyaluronan) and the pharmaceutical (vancomycin release) functions, aimed at a faster bone growth in the local presence of antibacterial effect.
**Conclusions** Present results show that biomolecular surface engineering can upgrade the properties of bone substitutes that exert mainly a mechanical function. Even if these encouraging in vitro results should be confirmed by forthcoming in vivo tests, it is clear that surface engineering applied to bone substitutes can play a fundamental role in the development of novel material of clinical interest.

### Hip resurfacing or traditional total hip replacement? A review of the recent literature

D. Lazzara*, A. Petrini

SC Ortopedia e Traumatologia, Ospedale San Giovanni di Dio (Florence, IT)

**Introduction** Resurfacing hip arthroplasty is becoming more popular for younger or with a greater functional demand patients, with degenerative hip disease. However, taking into account the data reported in the literature regarding revision rates and adverse reactions to any individual hyper-reactivity to metal debris (joining metal to metal) [1, 2], some authors question the supposed superiority of "resurfacing" compared to traditional total hip replacement. In this meta-analysis, we aimed to compare clinical and radiological results and complication rates of the two procedures, analyzing the most recent authoritative literature on the subject.
**Materials and methods** We performed a systematic review of all published articles (Medline, CINAHL, Amed, EMBASE, PubMed) until January 2011. We compared the clinical and radiographic results of resurfacing series with those of traditional total hip prosthesis and evaluated carefully the incidence and the type of complication rate reported by various authors.

**Results** 55 studies were analyzed with the results of 3957 resurfacing total hip replacement, compared with 3465 conventional total hip arthroplasties. The functional results are generally better or comparable after resurfacing, however, this type of prosthesis exhibits an higher incidence of complications such as aseptic failure of components, postoperative fractures, revision of the components, incidence of heterotopic ossification.

**Conclusions** Based on the analysis of the recent literature regarding the comparison of traditional and resurfacing hip arthroplasties, it can be concluded that, given the increased risk of heterotopic ossification, aseptic loosening and revision surgery after hip resurfacing, traditional total hip replacement is still superior in terms of implant survival [3].

**References**

1. Corten K, Ganz R, Simon J-P, Leunig M (2011) Hip resurfacing arthroplasty: current status and future perspectives. European Cells and Materials 21:243–258
2. Garbuz DS, Tanzer M, Greidanus NV, Masri BA, Duncan CP (2010) Metal on metal hip resurfacing versus large-diameter head metal on metal total hip arthroplasty. A randomized clinical trial. Clin Orthop Rel Res 468:318–325
3. Jiang Y, Zhang K, Die J, Shi, Z, Zhao H, Wang K (2011) A systematic review of modern metal on metal total hip resurfacing vs standard total hip arthroplasty in active young patients. J Arthoplasty 26(3):419–426

### Chondral lesions of the knee treated with A.M.I.C. technique: results of 97 cases after a 2-year follow-up

A. Siclari*, E. Boux

Ospedale degli Infermi (Biella, IT)

**Introduction** Treatment of cartilage lesions is a challenging problem for the orthopaedic surgeon. Recent progresses in tissue engineering and in mesenchimal cells use represent an effective new possibility: it has been demonstrated that scaffolds made in jaluronic or polyglicolic acid added with PRP (platelet rich plasma) are able to induce patient's mesenchimal cell recruitment and chondrogenesys.

**Materials and methods** Our procedure is entirely arthroscopic. From July 2007 to February 2009 we treated 111 lesions in 97 patients, 73 tibial and 38 femoral. 21 patients had a kissing lesion, the biggest lesion was 5 $cm^2$. Patients were evaluated with KOOS before intervention and after 3, 6, 9, 12 months.

**Results** Before surgery mean score was 50.25 pts: 24 months after intervention mean score was 82.85 ($p < 0.0001$), 32.60 pts higher. An arthroscopical second-look was performed 9 months post-operatory in 10 patients with bioptic samples of new cartilage: macroscopically it appears whiter than the other cartilage, smooth but with some little corrugation, with a consistence similar to normal cartilage, stuck to the bone. Istologically, in every case the scaffold disappeared and was replaced by a new jaline-like cartilage.

**Discussion** One patient underwent an arthroscopy 24 months after the implant: we found smooth and good quality cartilage, a bit darker than normal. Bioptic samples confirmed the presence of jaline cartilage. Every patient had a MRN of the knee 24 months after the procedure: the examinations demonstrated good reconstruction of chondral defect in each case.

**Conclusions** The AMIC appears a reliable technique for the treatment of cartilage lesions of the knee.

### Modular couplings in the hip joint prosthesis: titanium or CoCrMo?

P. Dalla Pria*[1], E. Sandrini[1], G. Rondelli[2], M. Juszczyk[1]

[1]Samo SpA. (Bologna, IT);
[2]C.N.R. (Milan, IT)

**Introduction** In the hip prostheses titanium modular couplings can be subjected to fatigue failure or cold welding due to fretting corrosion. To prevent these risks CoCrMo modular components are used.

**Materials and methods** Mechanical properties and corrosion resistance of modular necks in CoCrMo and Ti6Al4 V were compared. The modular necks were coupled to titanium stems in the same configuration in terms of length, anteversion and CCD angle. Fatigue tests were performed according to the ISO 7206-4 standard. Corrosion tests were performed according to the ASTM F1875-98 standard. Fretting is an electrochemical phenomenon, therefore the intensity of current between stem and neck were recorded. The liquid solutions were then analyzed with Atomic Mass Spectroscopy technique to determine the metal ions concentration. Coupling surfaces were observed by optical and electron microscopy to check the wear.

**Results** CoCrMo necks fatigue strength is about 20% higher than the Ti6Al4 V necks. The average gravimetric wear of the CoCrMo/Ti6Al4 V coupling was compared to the traditional Ti6Al4 V/Ti6Al4 V coupling (3.22 *vs.* 7.14 μg). Abrasion caused by fretting was found on the surface of the Ti6Al4 V necks. Conversely, the surface of the CoCrMo necks showed no wear and very low oxidation caused by the contact with the titanium stem. The analysis of the physiological solution showed no significant traces of metal elements released by the CoCrMo necks.

**Discussion** CoCrMo modular necks are mechanically stronger than the Ti6Al4 V ones and unlike these they are not subjected to fretting corrosion, surface abrasion and ions release.

**Conclusions** CoCrMo is more reliable than Ti6Al4 V in terms of mechanical strength and fretting. In case of revision CoCrMo necks disassembling is easier because cold welding does not occur. However, CoCrMo alloys are contraindicated for allergic patients.

### *AT15 – HAND AND WRIST*

### Bone substitutes in corrective osteotomies of distal radius

M. Rampoldi*, A.M. Artale, P. Mariano

Ospedale CTO, UOC Chirurgia della Mano (Rome, IT)

**Introduction** Purpose of the study is to report our experience with the use of calcium phosphate cement as an alternative to autologous bone grafting in corrective osteotomies of malunited distal radius fractures.

**Materials and methods** Twelve patients (7 male and 5 female, mean age 54 years) with a malunited distal radius fracture underwent surgical corrective osteotomy using fixed angle volar plate and calcium-phosphate cement as bone substitute. Patients were evaluated clinically (ROM, grip strength, Mayo wrist score) and radiographically (bone healing, radial tilt, radial inclination, ulnar variance) at a mean follow-up of 13 months (range 8–21).

**Results** All osteotomies healed. A mean preoperative dorsal tilt of 23° improved to 2° of volar tilt after surgery, radial inclination from 10°

to 22°, ulnar variance from +4 mm to 0.5 mm. Mean grip strength at the final follow-up measured 85% of the uninjuried side. Mean flexion–extension motion improved from 90° preoperative to 115°, radio-ulnar deviation from 25° to 43°, forearm rotation from 118° to 160°. According to the Mayo Wrist score system 3 patients had excellent results, 7 good and 2 fair; mean increase after surgery was 30 points.

**Discussion** Use of bone substitutes in corrective osteotomies avoids donor site related complications, reduces surgical time and ease the anesthetic management of the patient. Injectable calcium phosphate cement is a bioresorbable osteoconductive material with good biocompatibility and mechanical properties when associated to stable bone fixation. In our series all osteotomies consolidated without major complication and significant loss of correction.

**Conclusions** Bone substitutes represent a viable alternative to autologous bone grafting in corrective osteotomies of malunited distal radius fractures. Its advantages include ready availability, lack of donor site morbidity, easy application, safety. Conjunction with rigid stable fixation seems mandatory to maintain reduction and prevent loss of the achieved radiographic correction thus leading to satisfactory clinical results.

### Abnormal post-operative electrophysiological findings after carpal tunnel release: one-year follow-up

A. Merolli*, M. Lo Monaco, M. Masciangelo, C. Del Regno, F. Catalano

Università Cattolica (Rome, IT)

**Introduction** Recurrence of carpal tunnel syndrome after carpal tunnel release is a rare event, however practitioners may address again the patients to the surgeon if pain of any kind occurs in the treated hand. Electrophysiological evaluation is seek for diagnostic purposes. The electrophysiological outcome after carpal tunnel releases has been the subject of a limited number of studies. Our objective was to evaluate, during a one-year period, which changes occurred in the electrophysiological parameters and comparing them with the clinical outcome.

**Materials and methods** Twenty patients which received open-surgery in a consecutive series (no patient was excluded) were studied by measuring electrophysiological parameters at 1-month pre-operatively and, then, at 1-, 3-, 6-, 9- and 12-month post-operatively. Sensory nerve conduction velocity of median nerve (digit1-wrist and digit3-wrist) and radial nerve (digit1-wrist), as well as distal motor latency of median nerve, were measured. Furthermore, the shift between the latency of median and radial sensory nerve action-potentials (double peak shift), which is recorded following thumb stimulation, was investigated; this latter parameter is independent from possible inter-individual variations in nerve conduction velocity.

**Results** At 3 months, all patients experienced a distinctive remission in clinical signs and this was maintained afterwards; however, nerve conduction parameters did not returned to normality and this was true for most of them even after 12 months. The shift between the latency of median and radial sensory nerve action-potentials persisted in all patients at 12 months.

**Discussion** Despite surgical treatment let the electrophysiological parameters to move towards physiological values, normality was hardly reached; this sort of "electrophysiological scar" was particularly evident when the "double peak shift" was measured.

**Conclusions** These results lead us to suggest that abnormal post-operative electrophysiological findings, in the long term, should not substantiate the diagnosis of a poor outcome or recurrence as long as there is the clinical picture of remission.

### Personal experience about radial shortening osteotomy combined with a revascularization procedure in the treatment of Kienbock's disease

A. Marcuzzi*, R. Gabrieli, A. Russomando, A. Landi, M. Lando

Struttura Complessa di Chirurgia della Mano, Azienda Ospedaliero-Universitaria del Policlinico di Modena (Modena, IT)

**Introduction** Kienbock's disease, is a rare wrist disease. Although the aetiology is related to avascular necrosis of lunate, the actual cause of blood loss supply is not clear. The symptoms include usually pain and local swelling of the wrist, and functional limitation. At X-Ray lunate's deformation can be observed and in the advanced phases it is collapsed and fractured. During the last years several surgical treatments have been proposed for Kembock's disease, treatment. Surgical procedures performed can be classified in 4 groups: (1) biomechanical; (2) biological; (3) arthroplasties; (4) arthrodesis.

**Materials and methods** We report our experience about Kienbock's treatment with Hori's technique based on the implantation of a dorsal metacarpal artero-venous pedicle into the lunate, associated to radius shortening osteotomy. Between 1996 and 2008, in the Hand Surgery Unit of Modena, 21 patients underwent this procedure. The results were assessed with the use of pre and post-operative clinical (active and passive range of motion, grip strength, pulp and key pinch strength, pain evaluation) and radiographic studies (carpal height, Stahl's index, DISI or VISI instability and bone healing; 13 patients underwent MRI post operatively to evaluate bloody supply).

**Results** All patients underwent clinical and radiographic exams with a mean follow-up of 62 months (from 124 months, to 25 months). Post-operatively pain relief was achieved in the 86% of patients. Wrist motion and grip strength improved, 79% of patients reached more than the 80% of motion compared to the uninvolved side and the 58% of patients reached more than the 80% of grip strength compared with the uninvolved side. At radiographic evaluations carpal height was improved in the 40% of patients, Stahl index improved in the 63% of patients, and DISI and VISI instability didn't keep on. In all cases a bone healing was observed. In all 13 patient who performed RMI a partial bloody supply to the lunate was observed.

**Discussion** This technique is not easy to be performed.

**Conclusions** This technique allows to reach good results also in the advanced stages of the disease and it is a good alternative procedure to the partial wrist arthrodesis, because it often preserves a good wrist range of motion and anatomy.

### The F. Brunelli technique of looping the APL hemi-tendon to the first intermetacarpal ligament after trapeziectomy: one-year follow-up

L. Rocchi, A. Merolli*, A. Morini, C. Cotroneo, F. Catalano

Università Cattolica (Rome, IT)

**Introduction** Trapeziectomy and ligament reconstruction is preferred by surgeons concerned that telescoping of the thumb may reduce its function. However, theoretically ligamentoplasties are at risk to develop tendinosis or tendon rupture or a complex regional pain syndrome type 1. We tested the looping of a slip from the Abductor Pollicis Longus tendon around the first intermetacarpal ligament, aiming at a surgical treatment which does not require bone tunneling or looping around a tendon. Our results support the hypothesis that this new technique is a valid addition among treatments for carpometacarpal arthritis.

**Materials and methods** Forty-two patients were followed-up for one year. Each patient had subjective assessment for: pain; function (DASH) overall satisfaction. An objective assessment was enterprised for: first web span angle; abduction and opposition; key pinch; grip strength. Tests were performed prior to surgery, then at 3, 6 and 12 months. X-ray films were taken to monitor thumb height.

**Results** A significant improvement in all the parameters was measured in all patients. X-ray films showed the maintenance of a physiological height after one year. We recorded one complication of keloid and two of temporary dysesthesia but no cases of tendinosis, delayed rupture, or CRPS 1. Mean operative time was 27 min.

**Discussion** Simplification and search for a technique which avoids the looping around a tendon is the reason for which we performed this study. Advantages are the little number of steps, short time of surgery and comfortable postoperative prescriptions for the patient. The technique provides a distal suspension point (without bone tunnelling). It is quite respectful of anatomy and physiology, in minimizing the re-routing of functioning tendons.

**Conclusions** We propose it as an effective procedure both to expand the "weaponary" for treating the CMC osteoarthritis and/or to simplify the ligamentoplasties already in use.

### Treatment of unstable fractures of the distal radio with volar fixed angle plate

G. Manetti, L. De Luca*, M. Sangiovanni, D. Chicon Paez

SOD Ortopedia Traumatologica, A.O.U. Careggi (Florence, IT)

**Introduction** Because of demographic changes in industrialized countries, an increase by more than 50% of fractures of the distal radius is expected by 2030. Young patients usually show intra-articular and fragmented fractures mainly due to high energy trauma, whereas elderly patients' characteristics of these fractures is osteoporosis, which frequently leads to instability (following the criteria of Lafontaine) and secondary loss of reduction.

**Materials and methods** At our Department 33 patients (24 women, 9 men) were treated for fractures of the distal radius with volar fixed-angle plates. The mean age was 52 years (range 19–88). Plaque (DVR Hand Innovation) was positioned after the fracture reduction, through a surgical approach of Henry at the distal radius. In the post-operative patients retained splint for 1 week. Splint shall be removed daily to perform passive and active assisted mobilization. We evaluated the subjective postoperative results with the postoperative Michigan Hand Outcomes Questionnaire scores, and clinical and radiological results.

**Results** Patients were evaluated at an average follow-up of 14.5 months (range 4–20). Subjectively with the Michigan score the following results were recorded: function 85.75% (70–100%), daily activities 90% (80–95%), work 92.85% (85–95%), pain 9% (0–15%), satisfaction 86.9% (58.3–91.6%). The clinical recovery, compared to the contralateral, was: flexion 87%, extension 93%, ulnar deviation 89%, radial deviation 96%, Pronation98%, Supination 95%. Radiographically, we obtained an acceptable reduction in 91% of cases, all fractures were consolidated without loss of reduction. Complications were observed as transient irritation of the extensor pollicis longus, a lesion of the sensory branch of the radial nerve, long extensor of the thumb injury and an unaesthetic surgical scar.

**Discussion** There are many systems for the treatment of unstable fractures of the distal radius. Volar fixed-angle plates at the distal radius by allowing, through a simple gateway, a direct anatomic reduction of the articular surface, a stable internal fixation, a decrease of the period of immobilization resulting in early functional recovery. Also, there is a low complication rate (10%).

**Conclusions** This treatment is good for unstable distal radius fractures in patients, including elderly, with high functional demands, preventing a secondary loss of reduction, common with other types of treatment.

### *AT16 – HIP*

### Femoro-acetabular mini open osteoplasty vs. hip resurfacing in degenerative Toennis 2 stadium: results at 2-year follow-up

V. Bellotti*[1], R. Ledesma[1], C. Cardenas[1], D. Marinó[2], M. Ribas[1]

[1]Instituto Universitario Dexeus (Barcellona, IT); [2]Clinica Ortopedica, Università Federico II Napoli (Naples, IT)

**Introduction** The femoro-acetabular osteoplasty results in degenerative Toennis 2 patients are unpredictable. The purpose of our study is to compare the results in the short term of femoro-acetabular mini open osteoplasty and hip resurfacing.

**Materials and methods** We performed a prospective not randomized study in 99 consecutive patients suffering FAI, with a minimum 2-year follow-up. All of them were in a degenerative Toennis 2 stadium. Group A: 58 hip resurfacing. Group B: 47 femoro-acetabular mini open osteoplasties. Womac and Merle d'Aubigne scores were used for evaluation.

**Results** Average improvement of MDA scores was 4.9 points in group A and 2.3 in group B ($p < 0.001$). Average improvement of Womac scores was 42.3 points in group A and 21.7 in group B.

**Discussion** Hip resurfacing prosthesis showed better clinical and functional results on a short term follow-up in patients with Toennis 2 stadium.

**Conclusions** By this study's evidence, we recommend the implant of a hip resurfacing prosthesis in symptomatic patients in degenerative Toennis 2 stadium.

### Metal on metal THA: outcome and complications in 60 months. A prospective randomized study

C. Castelli*, F. Barbieri, R. Ferrari

USC Ortopedia e Traumatologia, Ospedali Riuniti di Bergamo (Bergamo, IT)

**Introduction** The aim of this study is to report on metal ion levels in patients in whom a cementless stem and cementless acetabular cup were placed both made of titanium but with a metal–metal bearing surface.

**Materials and methods** 53 subjects (May 2004–April 2006) were recruited into a prospective clinical trial. All patients received a cementless femoral stem (SummitTM), acetabular shell (PinnacleTM) with a 36 mm metal-on-metal articulation (UltametTM). Radiographs, Harris Hip scores were collected preoperatively, at 6, 12, 24, 48, 60 months. Whole blood samples were collected pre-operatively, at 1 week, 6, 12, 24, 60 months, frozen and analysed using mass spectrometry by an independent laboratory. Statistical comparisons were by using a two-tailed, nonpaired Students *t*-test.

**Results** Metal ion values were available for 25 patients at 60 months. The median values were 0.55 ug/l for Cr and 0.58 ug/l for Co. Median pre-operatively levels were 0.38 ug/l for chromium and 0.05 ug/l for cobalt There were five marginal outliers (patients who have either a Cr or Co level of > 1.5 ug/l). Maximum values were 3.00 ug/l (Cr) and 4.15 μg/l (Co). Over the whole cohort (53 patients) there were 2 revisions (after 5 years due to inflammatory local reactions) and no significant radiographic changes. In this series, within a 60-month follow-up, there were no radiographic changes, and no systemic reactions. The median metal ion levels reported are low (< 0.6 ug/l for both Cr and Co). In this study the ion levels have stabilised by 6 months supporting hip simulator data showing that appropriately designed metal–metal bearings 'run-in' quickly. Using the more consistent Cr levels there is a significant increase from pre-operatively levels to 6 months ($p = 0.003$). There are no differences between the 6 months and the 12, 24, and 60 months levels ($p = 0.39$, 0.57 respectively). Comparisons between different metal ion studies are notoriously difficult. However in absolute values these metal ion values at 60 m are below; the lowest reported values for surface replacement. The statistical significance of these differences is most known. However these observations are broadly in line with clinical and laboratory results.

**Conclusions** This study shows that correctly placed acetabular components with metal–metal bearings can consistently produce very low levels of whole blood metal ions without any significant outliers.

**Suggested readings**

1. Hart et al. (2008) Proc AAOS San Francisco, Vol 9, P082
2. Isaac et al. (2008) Trans ORS San Francisco, Vol 33:232
3. Venditolli et al. (2007) J Bone Joint Surg 89B:441–448

### Cam-type femoro-acetabular impingement: hip arthroscopy vs. anterior open approach

L. Pierannunzii*, M. Caforio, M. d'Imporzano

Istituto Ortopedico G. Pini (Milan, IT)

**Introduction** So far no comparative studies were published between open and arthroscopic management of femoro-acetabular impingement. The present study compared cam-impingement treatment with anterior open approach and arthroscopic procedure.

**Materials and methods** We compared 20 patients who underwent hip arthroscopy for cam-FAI (*group A*) and 20 patients who underwent a modified Smith-Petersen approach, without surgical dislocation (*group O*). The two groups were comparable as for age, sex, male-to-female ratio. Modified HHS, pain VAS, flexion range and satisfaction were calculated 6 weeks, 3 months, 6 months and 1 year after surgery. Significance level was set at 0.05.

**Results** The clinical outcome was significantly better in *group A* after 6 weeks, but the differences became non-significant at 1 year for all measured variables but flexion, that kept being higher in *group A*. In *group O* a patient developed bulky heterotopic ossifications and another one a lesion of the LFC nerve. In *group A* an abdominal extravasation of irrigation fluid and two hematomas occurred (with no permanent consequences).

**Discussion** The short-term clinical outcome of hip arthroscopy seems to be better than open surgery, likely because of the lower soft tissue damage. However 1 year later the differences, if any, are much smaller. Obviously this study has several weak points, mainly the short-term follow-up and the unblinded assessment of results.

**Conclusions** The tissue-sparing features of hip arthroscopy, together with the chance of dealing with intra-articular pathology, induce us to consider this procedure as a first choice in the management of cam-impingement, although long-term results of both techniques may be quite similar.

### Femoro-acetabular impingement. Arthroscopic osteoplasty: clinical and functional evaluation of 167 cases with a minimum 14-month follow-up

N. Santori*[1], A. Bertino[1], E. Theodorakis[2], V. Arceri[2], D. Potestio[3]

[1]Casa di Cura Quisisana (Rome, IT);
[2]Policlinico Umberto I (Rome, IT);
[3]Casa di Cura Villa del Rosario (Rome, IT)

**Introduction** Arthroscopic osteoplasty for femoro-acetabular impingement (FAI) is gaining more and more consensus mostly because of the considerable invasiveness of open surgery. Moreover, candidates for FAI surgery are usually young, and this evidence encourages the use of a less-invasive techniques.

**Materials and methods** From January 2006 to June 2010 we treated 151 hips in 136 patients with arthroscopic osteoplasty for femoro-acetabular impingement. Patients mean age was 34.8 years, and average follow-up was 17.6 months. No patients were lost to follow-up. All patients were studied preoperatively with conventional X-rays and magnetic resonance imaging. In 61 cases artro MRI was avalaible. Post-operatively, correction of the deformity was evaluated with X-rays. Co-existing intraarticular pathologies were recorded. Chondral damages were classified according to the Outerbridge classification. Clinical outcomes were measured with a visual analogue scale for pain, modified Harris Hip Score and improvement of range of motion.

**Results** 131 out of 167 treated hips had a torn acetabular labrum. In 114 hips there was an associated cartilage damage either of the femoral head or, more frequently, of the acetabular side. In most cases the damage consisted in cartilage – labrum disjunctions, presence of positive wave sign, Outerbridge grade I–II cartilage damage. Only in 28 cases the chondral damage was classified as Outerbridge grade III–IV. The modified Harris Hip Score gave excellent results in 105 patients (63%), good results in 43 patients (26%), acceptable results in 14 (8%) and bad results in 5 (3%). Visual analogue scale for pain scores significantly improved and we recorded an average improvement of hip internal rotation in flexion of 19 degrees. Patients with more severe cartilage lesions had a significant inferior clinical outcome.

**Discussion** The present study confirms that arthroscopic osteoplasty can provide results at least equivalent to those obtained with open surgery. Moreover, differently from open surgery, in this series no patients presented after surgery clinical and/or functional outcomes worst than pre-operatively.

**Conclusions** Clinical results obtained were directly dependent on the entity of cartilage damage and on the magnitude preoperative alfa angle.

### In vitro evaluation of hip joint contact areas during femoro-acetabular impingement (FAI) test

C. Signorelli*[1], N. Lopomo[2], T. Bonanzinga[2], G. Giordano[3], E. De Momi[1], G. Ferrigno[1], M. Safran[4], S. Zaffagnini[2]

[1]Dipartimento di Bioingegneria, Politecnico di Milano (Milan, IT);
[2]Laboratorio di Biomeccanica ed Innovazione Tecnologica, Istituto Ortopedico Rizzoli (Bologna, IT);
[3]U.O. Ortopedia e Traumatologia, Ospedale "Morgagni-Pierantoni" (Forlì, IT);
[4] Stanford University-Medical Center (Stanford, USA)

**Introduction** Due to the fact that often the femoro-acetabular impingement (FAI) occurs in combination with other pathologies related to the hip joint, the diagnosis could be difficult to achieve. To overcome these issues, different approaches have been proposed. The goal of the present study is to evaluate the dislocation of the contact areas during the physical examination commonly applied to make the femoro-acetabular diagnosis and whether they are able to effectively emphasize the presence of FAI. The following configurations were tested: 90° Flexion-Adduction-Internal Rotated, Extension-Adductio-External Rotation, Extension-Neutral-External Rotation. As a preliminary study to define the contribution of the soft tissues to hip range of motion, we tested three different conditions: intact hip, after removal of muscles and after partial capsulectomy.
**Materials and methods** Eight fresh frozen hips from four cadavers were tested. The specimens were securely fixed to a wood base in supine and neutral alignment, the load was manually applied and each test was repeated three times. An optoelectronic system (Blu-IGS, Orthokey, DE) was used to acquire the kinematics and anatomical data. The Cardano's angles have been calculated with the goal to evaluate the reproducibility of the performed motion. In particular we evaluated the Interclass Correlation Coefficient between the three repetitions of each movement. A spherical model was developed for both the acetabulum and femoral head surface to quantify the contact areas between them. To facilitate the description of the results we got, we used the common clock-face representation for the acetabulum.
**Results** The main ICC calculated was up to 0.7 for all the tests. We found the contact areas mainly localized between 8–12 o'clock during "Ext-Add-ExtRot" and between 9–11 o'clock during "Ext-Neu-ExtRot". While "90Flex-Add-IntRot" provoked a spreader dislocation of the contact areas. The previous results remain valid even after the skin and muscles removal and partial capsulectomy.
**Discussion** The present work does present some limitations. The use of cadaveric specimens implies the absence of muscular control. Moreover the load was manually applied without any control.
**Conclusions** The results confirm that the tests analyzed could be used as a method for verifying the presence of FAI. In particular, to analyze the most posterior region of the acetabulum, it is suggested to rich a configuration where the hip is both extended and external rotated. On the other hand the absence of contact areas in the most anterior portion of the acetabulum seems to challenge the effectiveness of these tests to quantify the anterior instability of the hip.

### Periacetabula osteotomy: preliminary experience of the Italian group for hip conservative surgery

A. Massè[1], A. Aprato*[1], L. Turchetto[2], G. Grappiolo[3], A. Campacci[4], R. Ganz[5]

[1]Ospedale San Luigi di Orbassano, II Facoltà di Medicina, Università di Torino (Orbassano-Turin, IT);
[2]Ospedale di Portogruaro (Portogruaro, IT);
[3]Istituto Clinico Humanitas (Rozzano-Milan, IT);
[4]Ospedale Sacro Cuore Don Calabria (Negrar-Verona, IT);
[5]The Hip Service, Insespital, Bern University (Berna, CH)

**Introduction** In literature many acetabular osteotomies are described but in the last years the periacetabular osteotomy as described by Ganz gained a huge popularity. This procedure is indicated for hip dysplasia in adult age and in children with closed bone growth plate. Aim of this study is a preliminary radiographic evaluation of the PAO performed by the Italian group for hip conservative surgery.
**Materials and methods** 15 periacetabular osteotomies were performed in 13 patients for hip dysplasia. Surgical treatments were performed between 2008 and 2010. Average patients' age was 21 years at time of surgery. Pre-operative and the last X-rays were evaluated for cervico-diaphysial angle, later centre edge, acetabular index, and the presence of a cross-over sign. Average follow-up was 1.4 years.
**Results** Average preoperative cervicodiaphysial angle was 138° (range, 126°–150°), average acetabular index was 28° (range, 20°–44°). Average LCE was 4° (range, −34°–26°). Cross over sign was positive in 4 hips. Average post-operative cervicodiaphysial angle was 137° (range,128°–140°), average acetabular index was 10° (range, 8°–14°) and average LCE was 28° (range, 28°–36°).
**Discussion** This study shows radiographic results similar to other published casuistics with a good correction of the evaluated signs of hypocoverage; but a long term re-evaluation is mandatory to confirm the hypothesis that PAO could be a satisfactory procedure in our hands. This study is limited by the lack of a clinical evaluation, for the small number of patients and the short follow-up but represents the first experiences of this group with this technique.
**Conclusions** Our study cannot claim to add significant evidence to the efficacy of the PAO but several published studies showed good long term results, and these data suggest us to continue with this technique although PAO presents different surgical tricks and a long learning curve.

### Biometrical hip reconstruction using the fitmore stem

M. Bombelli*, M. Memminger

Ospedale Centrale Bolzano (Bolzano, IT)

**Introduction** Beside perceiving the final goal of a long lasting painfree articulation, modern total hip replacement should also fulfill the functional requirements of a increasingly demanding and younger population. The replaced hip must guarantee a primary solid mechanical stability, allow for a rapid biological integration and must represent the tool for the most accurate and biometrical correct articular reconstruction according to the pre-existing anatomical morphology. Tissue sparing surgery represents the connection ring between the theoretical advantages of a correct articular restoration and the actual outcome.
**Materials and methods** A radiographic study was performed on 100 patients operated with an hemispherical cup and the fitmore stem with particular regard to offset and leg length comparing the postoperative result with the contralateral non affected side. The comparative analysis was performed on 75 patients excluding cases of contralateral arthritis and cases that could not be matched because of other reasons.

**Results** A subtle mean difference of −0.54 mm could be achieved regarding the post-operatively offset. In one third of the cases the offset matched exactly the contralateral value, in one third of the patients it differed by a mean value of −3.03 mm, in the remaining third it was increased on average by 1.84 mm. Leg length was increased by an average of 0.44 mm compared to the contralateral side, in 60% of the cases leg length was equal, in 22% the leg resulted shorter on average by −2.6 mm, 17% of the cases longer on average by of 3.52 mm. Considering a tolerance of ± 5 mm, leg equal length was restored in 95% of the cases.
**Discussion** The fitmore's stem design is based on different a radius of curvature according to the distinct femoral anatomy in varus or valgus hips compared to regular hips. Its considerable number of sizes in each family group allows a precise geometrical reconstruction of the new articulation provided preoperative planning is reproduced during surgery by an accurate surgical technique.

### Mini-invasive treatment of necrosis of the femoral head with the use of biotechnology

G.M. Calori*, M. Bucci, P. Fadigati, E. Mazza, M. Colombo, C. Ripamonti

C.O.R., Istituto Ortopedico G. Pini (Milano, IT)

**Introduction** Osteonecrosis of the femoral head at an early stage is still a challenge for orthopaedic surgeons, mainly because it affects young patients (between the third and fifth decade of life). Total hip arthroplasty remains the gold standard treatment although it is a drastic choice. For this reason various alternative techniques have been proposed (artrodiastasi, Mont technique, vascularized fibular transplant, with core decompression or not insertion of fixation and growth factors) aiming at preserving the femoral head.
**Materials and methods** Starting from the idea of core decompression we developed a new surgical technique with a dedicated instrument that combines the removal of necrotic tissue (core decompression) with the inclusion of growth factors (rh-BMP-7) and an equine bone scaffold flexible enriched with autologous mesenchymal cells. We treated 18 patients with this method out of a total number of 20 affected patients, all patients had more than one stage IIIc according to Steinberg classification. Each patient was studied pre-operatively by clinical, radiographic, MRI and CT. Patients were then re-evaluated clinically and radiographically at 1, 3, 6, 12 and 24 months, an initial CT scan was performed at 3 months followed by a control MRI at 6 months.
**Results** We obtained excellent results in 17 cases of clinical and imaging (non-progression of necrosis, cortical conservation profile of the femoral head, the absence of structural collapse and good articulation) at 18-month medium follow-up; 2 cases of failure with persistence of symptoms and progression of necrosis; 1 case of sub-trochanteric fracture after trauma.
**Discussion** The technique appears to offer good results especially given the short time required for surgery, the simplicity of the surgical technique and ability to save still healthy joint in patients young.
**Conclusions** With the encouraging results obtained we can conclude in favour of the application of biotechnology in polytherapy in the treatment of necrosis of the femoral head until a stage IIIc. The treatment does not preclude further surgery and prosthetic replacement and is much less invasive and well tolerated by most patients when compared with vascularized fibula autograft.

### Use of bone allografts added with mesenchymal stem cells and growth factors in the treatment of necrosis of femoral head

P. Di Benedetto*, V. Cainero, R. Gisonni, A. Beltrame, C. Concina, A. Causero

Clinica Ortopedica, Azienda Ospedaliero-Universitaria di Udine (Udine, IT)

**Introduction** The necrosis of the femoral head is a fairly widespread problem especially in young people between 30 and 50 years. Excluding post-traumatic forms, the etiology is not yet fully understood. There is a higher incidence in certain groups of patients as in patients receiving high-dose steroid therapy for long periods, in organ transplants or patients with SLE. Several studies in the literature show that core decompression is the method of choice for the treatment of necrosis of the femoral head in the early stages, with good clinical results in the short term. Given the numerous cases of progression and collapse of the femoral head after core decompression some authors have proposed the use of allograft bone grafts.
**Materials and methods** From May 2008 to October 2009, 12 patients (2 in stage I, 9 in stage II and 1 in stage IV according to the ARCO classification) underwent core decompression with bone graft added to mesenchymal stem cells and PrP. Mesenchymal stem cells were obtained from bone marrow aspirate from the iliac crest. Patients were evaluated in the preoperative and at 3, 6, 12, 24 months with clinical evaluation including Harris Hip Score (HHS), radiographic and MRI evaluation.
**Results** The patients have shown complete recovery of motion in the absence of hip pain and return to normal daily activities. Furthermore, a progressive improvement of HHS values was obtained, passing from the mean pre-operative 38.3 ± 9.9 to 79.9 ± 7.5 at 3 months ($p < 0.0001$), to 85.0 ± 8.5 at 6 months ($p < 0.0001$) To 84.9 ± 16.9 at 12 months ($p = 0.0002$) and 88.6 ± 4.1 to 24 months ($p < 0.0001$). The patient with stage IV sec. ARCO underwent hip artheroplasty. In the radiographic and MRI evaluation was not observed any progression of lesions (Arlet and Steinberg classifications, respectively), except in the patient with femoral head collapse.
**Conclusions** In conclusion, the results show that core decompression combined with bone grafting, mesenchymal stem cells and PrP may be a useful and effective method for the treatment of necrosis of the femoral head at an early stage. These data should be confirmed, however, extending the follow-up and increasing patient's number.

### Intra-articular injection of autologous growth factors for degenerative pathology of the hip joint

E. Costabile*[1], A. Crescibene[1], F. Togo[1], H. Almolla[2], M. Napolitano[3], C. Giannuzzi[2], M. Bossio[3], G. Guido[2], S. Matera[3]

[1]U.O.C. di Ortopedia e Traumatologia, Azienda Ospedaliera di Cosenza (Cosenza, IT);
[2]U.O.C. di Radiologia, Azienda Ospedaliera di Cosenza (Cosenza, IT);
[3]U.O.C. di Immunoematologia e Medicina Trasfusionale, Azienda Ospedaliera di Cosenza (Cosenza, IT)

**Introduction** Articular degenerative disease is very common among the population and it is a frequent cause of disability. Harris considers that almost all cases of hip joint arthrosis are due to some kind of development anomaly or to collagen derangement. The cellular adaptation to the articular damage determines pathological modifications of the liquid content in the collagen structure and in the proteoglycans composition, this results in cartilage degeneration and subsequent reduction of the articular space.
**Materials and methods** We treated 11 patients with age superior to 45 years, having a diagnosis of degenerative disease of the hip joint for more than one year. Each patient had a series of intra-articular injections under ultrasound guidance of plasma rich platelets (PRP) for a total of 4 injections applied on a weekly basis. Preliminary clinical evaluation included administration NRS and WOMAC questionnaires, these tests were repeated at the end of treatment and yet another time at 6 months from the treatment end. Average patients age is 55.18 years (46–72); 7 males and 4 females were included; 8 patients presented primitive disease, and the remaining 3 had secondary disease; in 7 cases the left hip joint was affected and in 6 cases it was the right side which was affected. Two patients had X-ray examination classified as grade IV of Kellgren-Lawrence, 7 were grade III, 1 was grade II and 3 were grade I. Average BMI was 25.13 (21–31).
**Results** Good improvement was evaluated according the NRS scale, pre-treatment scale was 7 ± 2.30, while post-treatment scale resulted in 3.46 ± 2.02, 8 patients presented an average scale of 1.75 ± 1.03 at 6 months follow-up. Average WOMAC score was 41.69 ± 22.78 before treatment, while evaluation a week after the last injection gave a 78 ± 12.06 score. Patients who completed 6 months follow-up scored on average 86.25 ± 4.59 points. In no case adverse effects or allergic reactions occurred.
**Discussion** We intend to present a pilot study, the target is to propose autologous blood platelet derived growth factors as treatment for degenerative pathology of the hip joint.
**Conclusions** The method is very simple, cheap, safe and efficient in reducing pain and recovering articular function.

### Both columns fractures of the acetabulum: posterior column reduction through the ilio-inguinal approach (preliminary results)

A. Nicodemo*, G. Governale, A. Stucchi, M. Capella, M. Deregibus, A. Massè

Ospedale San Luigi di Orbassano, Università di Torino (Turin, IT)

**Introduction** Between 2002 and 2009 we operatively treated 193 acetabular fractures. Among these, 44 both-columns fractures according to the classification of Letournel and Judet were reviewed in order to evaluate the preliminary results.
**Materials and methods** 44 cases of open reduction and internal fixation of displaced both columns fractures were studied, 40 hips were operated with the ilio-inguinal approach alone, 1 with Kocker Langenbeck and Smith petersen combined. The 3 remaining hips were operated with a double Kocher-Langenbeck and ilio-inguinal approach. Every case was evaluated on X-rays according to Matta criteria and clinically with the Harris hip score and the WOMAC score at a mean 37-month follow-up (range, 13–76 months) after the injury.
**Results** The mean Harris hip score was 85.8% (range, 30%–100%) while the mean WOMAC score was 88.3 points (range, 39.1–100). The main complications were 4 early post-traumatic arthritis operated by hip replacement. One patient developed a gastric carcinoma and got lost at follow-up.
**Discussion** The anatomic reduction is the first goal that surgeons must achieve in order to obtain good results. The reduction quality was adversely affected by the complexity of the fracture, associated injury of the femoral head, the patient's age, and the interval between the injury and the reduction. Most of the fractures (91%) were correctly reduced using the ilio-inguinal approach alone, through the medial window of that approach and with the help of dedicated surgical tools.
**Conclusions** These preliminary results indicate that it is possible to reduce both columns fractures through a single approach in order to decrease infections, operating times, skin scars and rehabilitation times. Surgeon experience and a long learning curve can help in decision making and in obtaining an anatomical reduction with a low rate of post-operative complications in this very hard orthopaedic field.

### *AT17 – KNEE 1*

### Comparison between manual and computer assisted techniques in patients treated with open wedge high tibial osteotomy for varus knee deformity

R. Iorio*, M. Pagnottelli, A. Vadalà, S. Giannetti, P. Di Sette, P. Papandrea, F. Conteduca, A. Ferretti

UOC di Ortopedia e Traumatologia, S. Andrea Hospital (Rome, IT)

**Introduction** Open wedge high tibial osteotomy is an established technique to treat symptomatic varus malaligned knees. The purpose of our study is to compare the clinical and radiological results of two groups of patients treated with either conventional or computer assisted open wedge high tibial osteotomy. We established the goals of correction in obtaining a postoperative mechanical axis as between 2 and 6 degrees valgus and an increase or decrease of posterior tibial slope between −2 and +2 degrees.
**Materials and methods** In our study we prospectively followed-up 24 patients (27 knees) affected by varus knee deformity and operated with OWHTO. They were randomly divided in two groups, A (11 patients, 13 knees) and B (13 patients, 14 knees). All patients were evaluated both clinically and radiologically, pre-operatively and post-operatively. For clinical assessment, two questionnaires were used: the Knee Society Score and the Modified Cincinnati Rating System Questionnaire. All patients were radiologically evaluated with a comparative lower limb weight-bearing digital radiograph, a standard digital antero-posterior (A-P), a latero-lateral (L–L) radiograph of the knee and a Rosemberg view.
**Results** At mean follow-up of 39 months we reassessed all patients. Clinical evaluation showed no statistical difference ($p > 0.05$) between the two groups. Radiological results showed an 24% (3 patients) reproducibility in achieving a mechanical axis of 182°–186° in the group A compared to a 86% (12 patients) reproducibility in achieving a mechanical axis of 182°–186° in the group B ($p = 0.0392$). Furthermore, only in the 24% of patients treated with the conventional technique, we obtained an increase or decrease of posterior tibial slope between −2 and +2 degrees, in patients treated with the navigation system we obtained this result in 100% of cases ($p < 0.0003$).
**Discussion** In literature, studies concerning patients treated with HTO, agree in describing a good long-term clinical results, but there is considerable controversy regarding the ideal valgus correction angle. The use of navigation system for the tibial osteotomy has been recently introduced and studies present in literature are still discordant concerning its utility. The aim of this study is to evaluate the accuracy and reproducibility of the navigation system in the correction of varus knee deformity.
**Conclusions** High tibial osteotomy with navigator is more accurate and reproducible in the correction of the deformity in both coronal and sagittal plane compared with standard technique.

### Use of an electronic system for balancing a total knee arthroplasty

F. D'Angelo*, T. Binda, P. Cherubino

Clinica Ortopedica, Università dell'Isubria (Varese, IT)

**Introduction** The soft-tissue balancing plays a key role in the result of a total knee replacement. Currently it is a key operator dependent, linked to the surgeon's experience accumulated over the years. In order to standardize this feature a new mechanical system for intra-operative assessment of ligament tension was developed This system measures objectively the strength in the two compartments before the final cuts are made, thus balancing the ligamentous tension with a modification in femoral component rotation.
**Materials and methods** Between April 2010 and January 2011 E-Libra (Synvasive Technology Inc.) tool was used in 50 consecutive total knee arthroplasties. The mean age was 69.55 years (range 47–85 years), 14 males and 36 females. All operations were performed by a single surgeon, using the same surgical approach, median parapatellar aggression line, and using the same model of a prosthetic posterior cruciate sacrifice. All patients were evaluated clinically using the Knee Society Score before the operation and subsequent controls.
**Results** In none of the 50 cases lateral release was performed, the patients included in this study reported no complications peri-or post-operative. The clinical evaluation showed an improvement in both scoring (82.7 points and 88.2 respectively for clinic and function).
**Discussion** To sum up, the use of this device provides patient-specific rotational orientation of the femoral component. As such, the femoral component is no longer tied to conventional methods such as the epicondylar axis, the Whiteside line or 3° of external rotation with reference to the posterior condyles, which are typical anatomical landmarks often used. However, these anatomical references lead to a decrease in asymmetry of the flexion space in about 10% of cases.
**Conclusions** Due to the short follow-up period, only favourable impressions of the simplicity of the instrument and the reproducibility of the ligament balancing can be expressed.

### Prospective randomized comparison of double bundle vs. anatomical single bundle ACL reconstruction: two-year follow-up

A. Ampollini*, F. Morici, S. Nicoletti, P. Adravanti

Casa di Cura Città di Parma (Parma, IT)

**Introduction** Single bundle ACL reconstruction with ST/G is increasingly used in a large number of patients and it allows obtaining very good clinical and subjective results; however, functional tests show a persistent rotational instability. Biomechanical studies seem to indicate that double bundle ACL reconstruction (DB) allows to obtain increased anterior and rotational stability compared to single bundle (SB). The aim of this prospective randomized controlled study was to compare the clinical outcome of patients treated either with a personal SB (free-hand trans tibial femoral tunnel) or with a DB technique (Out-In for PL femoral tunnel/In–Out for AM femoral tunnel).
**Materials and methods** A total of 60 patients with complete ACL rupture (age range, 16–40 years) were prospectively randomized to SB ($n = 30$) and to DB ($n = 30$) groups. Patients were evaluated pre-operatively and after surgery by Lysholm score, IKDC form and KT-2000, with a minimum follow-up of 24 months. No significant differences in background factors (age, time from injury, sex) between the two groups were observed ($p > 0.05$). Homogeneity was also found in term of pre-operative Lysholm score, IKDC and KT-2000 between SB and DB group ($p > 0.05$).
**Results** During follow-up 5 patients of SB group and 4 of DB group were lost. One patient of DB group was excluded from the study for neo-ligament rupture. So the total number of patients at two-year follow-up was 25 for both groups. After 24 months from surgery both groups showed significant improvements in Lysholm, IKDC and KT-2000 ($p < 0.001$) compared to pre-operative observations; however, no significant differences were observed in term of post-operatively values between SB and DB groups (Lysholm score 98.14 ± 10.03 and 97.22 ± 12.82; IKDC normal + nearly normal 95% and 100%; KT-2000 1.68 ± 1.26 and 1.03 ± 1.92 for SB and DB respectively; all $p > 0.05$).
**Discussion** In our study we did not detect any advantage in using double bundle ACL reconstruction in term of clinical outcome and knee stability in the short-medium term. In our opinion, this lack of difference in our study could be related to the anatomical positioning of the AM bundle in the single bundle technique.

### Use of transepicondilar aligner device (TAD) in 900 TKA implanted between December 2007 and December 2010

G. Pipino*[1], D.C Vaccarisi[2], G. Maffei[1]

[1]Ospedale Villa Regina (Bologna, IT);
[2]Ospedale Civile Imola (Imola, IT)

**Introduction** The transepicondilar aligner device (TAD) is a mechanic system that allows the right rotational positioning of knee femoral prosthesis component based on transepicondilar axis. It also helps more rapid space in flexion calculation, can be used with every prosthesis instrumentation, finally it is easy to use and uptake. Actually, we are studying a new evolution of this device that will allow to calculate both transepicondilar line and space in flexion, ensuring a superior prosthesis stability and duration.
**Materials and methods** All 900 TKA have been implanted by the same expert surgeon through a miniparapatellar approach, using three different prosthesis models (PS mobile platform, CR mobile platform, CR fixed platform).
**Results** Results were evaluated using OKSS. Transepicondilar line rotational angle average, calculated by TAD with posterior reference was of 4.5° ± 0.5° of external rotation in valgus knee and 3.5 ± 0.5 of external rotation in varus knee. This angle was calculated both in operation room and on pre-operative CT. Only in 8 cases of varus knee, transepicondilar line rotational angle was of 1° of internal rotation, they were 8 varus congenital internally rotated tibias.
**Discussion** Identification of the transepicondilar line is easy, it is enough to feel epincondyles, the more prominent markers in L–L, to trace the line and then to set TAD, or you must calculate the line on pre-operative CT and then to set TAD according with its angle. Space in flexion is more difficult to calculate although it is very important for prosthesis kinematic. An exact calculation of transepicondilar line and space in flexion generates a fulcrum with constant moment during implant work, realizing right compression and slipping forces that do not generate dissecting lines that deforms polyethylene.

**Conclusions** In our study of 900 TKA implanted with TAD, the common factor was almost total breakdown of patellar pain, prosthesis stability, more easy calculations and realization space in flexion with improvement of prosthesis kinematic, no patellar prosthesis, and subjective and objective feeling of almost normal joint with ROM between 0°–130°.

### Preliminary results of a ceramic femoral component in total knee arthroplasty

F. Benazzo*[1], S.M.P. Rossi[1], P. Bergschmidt[2], R. Bader[2], D. Ganzer[3], C. Hauzeur[3], C. Lohmann[4], D. Tigani[5], N. Rani[5], C. Zorzi[6], V. Madonna[6], E. Saura Mendoza[7], G. Kundt[8], H.R. Bloch[9], S. Burelli[9], G. Carli[9], W. Mittelmeier[2]

[1]Clinica Ortopedica e Traumatologica, Fondazione IRCCS Policlinico San Matteo (Pavia, IT);
[2]Department of Orthopaedics, University of Rostock (Rostock, DE);
[3]Department of Orthopaedics, Klinikum Neubrandenburg, Klosterberg 1° (Altentreptow, DE);
[4]Department of Orthopaedics, University of Magdeburg (Magdeburg, DE);
[5]Istituti Ortopedici Rizzoli, 7[a] Divisione (Bologna, IT);
[6]Ospedale Sacro Cuore Don Calabria, Clinica Ortopedica e Traumatologica (Negrar, IT);
[7]Hospital General Universitario De Elche (Alicante, Spain);
[8]Department of Medical Informatics and Biometry, University of Rostock (Rostock, DE);
[9] Lima Corporate Spa (Villanova di San Daniele-Udine, IT)

**Introduction** Total knee arthroplasty (TKA) is one the most successful procedures in orthopaedic surgery, with good outcomes in pain reduction and function recovery. However, the increase in life expectancy and in patients' needs have led to develop new designs and materials. The introduction of alternative materials, as ceramic, is important because allows the reduction of polyethylene wear, one of the most important causes of mobilisation, and represents a solution for the growing problem of metals hypersensitivity. The goal of this prospective study is to evaluate clinical and radiographic outcomes of a total knee prosthesis with an innovative femoral ceramic component.

**Materials and methods** 108 patients (110 knees) underwent a total knee arthroplasty with a ceramic BIOLOX® delta femoral component (Multigen Plus Ceramic Knee, Limacorporate SpA, Villanova di San Daniele, Udine) in 7 European centres. The average age of the patients was 67.7 ± 5.9 years. They were female in 81 cases (73.6%) and male in 29 (26.4%) and had an average height and weight respectively of 165.9 ± 7.7 cm e 79.0 ± 11.7 kg (BMI 28.7 ± 3.5). They mainly suffered from primary osteoarthritis (93%), osteonecrosis (4%), rheumatoid arthritis (2%) and, lastly, secondary osteoarthritis (1%). Clinical evaluation was performed preoperatively, at 3, 12, 24 and 60 months using the Hospital for Special Surgery Score (HSS), WOMAC score e SF-36 questionnaire. Radiographic evaluation was planned at the same time points using the immediate postoperative as baseline. The statistical analysis was performed with the Friedman (FR) and Wilcoxon (WI) tests, with $p$-value $< 0.05$.

**Results** The minimum follow-up was 12 months. The average HHS value significantly improved from 55.5 ± 11.5 in the pre-operative to 77.2 ± 10.3 and 85.6 ± 8.9, respectively after 3 and 12 months (WI test $p = 0.001$). Significant improvements were identified in all domains, especially pain and ROM (WI test: pain $p < 0.001$, function $p < 0.001$, muscular strength $p < 0.001$, deformity $p < 0.001$). Radiolucent lines have been identified in 3 cases, but without signs of progression. No implant-related complications, signs of mobilisation and failures occured.

**Conclusions** Short-term clinical and radiographic outcomes in the use of the ceramic in knee femoral component arthroplasty are very promising. A long-term follow-up is necessary to confirm the results and evaluate the survivorship rate of the implants.

### *AT18 – KNEE 2*

### Medial open wedge high tibial osteotomy: hydroxyapatite-based spacers without bone graft treatment

P.P. Cané*, M. Fravisini, V. Cigna

U.O. Ortopedia Sol et Salus (Rimini, IT)

**Introduction** Medial Open wedge osteotomy envisages the creation of a bone defect on tibial tuberosity using a drill and osteotome technique filling the gap using autologous bone withdrawn from the iliac crest. Nevertheless, avoiding autologous bone transplantation, reduces the drawback related to associated morbidity and decreases the time of surgical procedure [1]. The aim of this clinical investigation was to demonstrate the effectiveness of a hydroxyapatite based spacers (ENGIpore) to fill the bone gap after osteotomy. An improvement of post-operative course was evaluated as well.

**Materials and methods** In the prospective randomized study 70 patients underwent a medial open wedge high tibial osteotomy. All surgical procedures were performed by the same surgeon using standard surgical procedure and the same plate with locked screws was used for fixation in all cases. Patients were randomly divided in two groups (n = 35): *Group I* received no treatment and the gap was left empty (*Control Group*), in the *Group II* (*Treatment group*), the patients were treated with biomimetic ENGIpore bone spacers based on hydroxyapatite. All patients followed a standard post-operative rehabilitation protocol.

**Results** At follow-up, no significant differences were observed between the two groups after X-ray evaluation, although in the ENGIpore group the clinical evaluation exhibited a shorter recovery of normal activity with less pain in comparison to the untreated group.

**Conclusions** The results of this prospective trial demonstrated that the use of a hydroxyapatite bone graft as a filler in the open wedge high tibial osteotomy lead to a faster recovery and pain relief.

**Reference**

1. El Assal MA, Khalifa YE, Abdel Hamid MM, Said HG, Bakr HM (2010) Opening-wedge high tibial osteotomy without bone graft. Knee Surg Sports Traumatol Arthrosc 18(7):961–966.

### Isolated medial patellar femoral ligament reconstruction for recurrent patella dislocation: a prospective cohort study

M. Ronga*[1], F. Oliva[2], U. Longo[3], V. Teata[4], G. Capasso[5], N. Maffulli[6]

[1]Dipartimento di Scienze Chirurgiche Ricostruttive e Tecnologie Avanzate, Università dell'insubria (Varese, IT);
[2]Department of Orthopaedics and Traumatology, University of Rome "Tor Vergata" School of Medicine (Rome, IT);
[3]Department of Orthopaedic and Trauma Surgery, Campus

Biomedico University (Rome, IT);
[4]Department of Sports Medicine, Olympic Center (Angri, IT);
[5]Department of Orthopaedics and Traumatology, Second University of Napoli (Naples, IT);
[6]Centre for Sports and Exercise Medicine, Queen Mary University of London (London, UK)

**Introduction** Several medial patellofemoral ligament (MPFL) reconstruction procedures have been proposed to manage recurrent patellar dislocation. No clear superiority of a surgical technique has been evident over another. This prospective cohort study evaluates clinically and functionally a selected group of patients who underwent isolated anatomic MPFL reconstruction with autogenous hamstring grafts for chronic patellar dislocation. Hypothesis: The reconstruction of the MPFL using hamstring passed through a double patellar transverse bony tunnel technique is an effective management modality for recurrent patellar dislocation in patients without any predisposing factors.
**Materials and methods** Twenty-eight patients with chronic patellar instability without any anatomic predisposing factors, experiencing recurrent unilateral patellar dislocation, were included in the study. The patients were regularly followed-up postoperatively at 2, 4, 8, 12, and 24 weeks, and then annually. Evaluation included the modified Cincinnati and the Kujala scores, anthropometry, plain radiography, and isokinetic dynamometry.
**Results** The average follow-up was 3.1 years (range, 2.5–4 years). The mean modified Cincinnati score increased from 52 preoperatively to 89 ($p = 0.001$). The mean Kujala scores increased from 45 preoperatively to 83 ($p = 0.03$). The muscle volume of the thigh of the operated limb increased with time, but remained less well developed than the nonoperated limb ($p = 0.04$). The mean Insall-Salvati index was 1.1 (range, 0.9–1.2) preoperatively and remained within normal range (1.1 [range, 0.9–1.2]) ($p = 0.07$), at latest follow-up. Significant isokinetic strength differences were found between the operated and the contralateral limbs ($0.05 < p < 0.006$), even at the latest follow-up. Three patients experienced a new patellar dislocation.
**Discussion** The high percentage of success (79.2%) and the low patellar dislocation rate (10.7%) in our series probably depends on patient selection. The percentage of redislocation is comparable with that in previous studies. Our technique does not preclude further surgical procedures, such as the Elmslie-Trillat osteotomy. Inhibition of the knee extensor muscle complex seems to persist despite subjectively successful surgery, and despite appropriate postoperative rehabilitation, muscle atrophy may be difficult to overcome. It is possible that heavy resistance training may have to be implemented to improve these findings.
**Conclusions** MPFL reconstruction using hamstring tendon passed through a double patellar transverse bony tunnel technique is a safe, reliable management option for recurrent patellar dislocation in patients without any predisposing anatomic factors. The superiority of this technique over others can only be established in an adequately powered randomized controlled trial with long term follow-up.

### Pulsed electromagnetic field stimulation in TKA: a prospective randomized clinical study

S. Nicoletti*, A. Ampollini, P. Banchini, P. Adravanti

Casa di Cura Città di Parma (Parma, IT)

**Introduction** Pulsed electromagnetic field stimulation (PEMF) is known to exert an anti-inflammatory effect on several tissues, including cartilage and subchondral bone. The purpose of our study was to evaluate the clinical effect of PEMF stimulation on patients who undergone TKA on pain, joint swelling and functional recovery.
**Materials and methods** Before surgery, 26 patients candidate to cemented PS TKA were randomly assigned to experimental (A, n = 12) and control groups (B, n = 14). Inclusion criteria were age between 60–85 years, gonarthrosis, varus and valgus knee malalignment < 20°; exclusion criteria were previous surgery to the same knee, ipsilateral hip prosthesis, rheumatoid arthritis, autoimmune and systemic disease, BMI > 30 kg/m$^2$. Both groups of patients received the same standard peri- and post-operative protocol. All patients received the same rehabilitation protocol, starting from day 1. Just patients of group A received the PEMF stimulation (I-ONE therapy, IGEA, Carpi, Italy) for 4 h/day for 60 days, starting from day 3 post-surgery. Patients were evaluated at 1, 2 and 6 months after surgery using VAS Knee Scale, Knee Score and SF-36 questionnaire. Joint swelling was also evaluated.
**Results** No significant differences in background factors between the two groups were observed ($p > 0.05$). Homogeneity was also found in term of pre-operative SF-36, VAS, Knee Score and swelling between group A and B ($p > 0.05$). After 1 month from surgery just patients of group A showed a significant improvement respect to baseline (day 3 after surgery) in term of functional recovery ($p < 0.05$); however starting from 2 months these values were comparable between the two groups, and they both were significantly higher than the baseline level ($p < 0.001$). The knee rating values were very similar in the two groups, with significant differences respect to baseline ($p < 0.001$) already starting from 1 month after surgery. Joint swelling was significantly reduced in group A respect to group B ($p < 0.05$) both at 1 and at 2 months, whereas at 6 months data between the two groups were comparable. Pain, evaluated by VAS knee scale, was significantly reduced in both groups already from 1 month after surgery; however at this time point we observed a significant difference between the two groups ($p < 0.05$), with better results in group A.
**Discussion** Even if in a small sample size, patients treated with I-ONE therapy show a better outcome in term of pain, functional recovery and joint swelling, above all in the first month after surgery. This can be due to the anti-inflammatory effect of PEMF on the whole joint tissues.

### The three-in-one procedure for recurrent dislocation of the patella in skeletally immature children and adolescents

M. Ronga*[1], F. Oliva[2], U. Longo[3], V. Testa[4], P. Cherubino[1], N. Maffulli[5]

[1]Dipartimento di Scienze Chirurgiche Ricostruttive e Tecnologie Avanzate, Università dell'Insubria (Varese, IT);
[2]Department of Orthopaedics and Traumatology, University of Rome "Tor Vergata" School of Medicine (Rome, IT);
[3]Department of Orthopaedic and Trauma Surgery, Campus Biomedico University (Rome, IT);
[4]Department of Sports Medicine, Olympic Center (Angri, IT);
[5] Centre for Sports and Exercise Medicine, Queen Mary, University of London (London, UK)

**Introduction** Recurrent patellar dislocations are common injuries in children and adolescents. This prospective cohort study evaluates clinically and functionally a selected group of skeletally immature individuals patients who underwent the 3-in-1 procedure for chronic patellar instability.
**Materials and methods** Twenty-five skeletally immature patients (age, 13.5 ± 3.8 years), without any anatomic predisposing factors, who were suffering from recurrent unilateral patellar dislocation were included in the study and followed until skeletal maturation. The 3-in-1 procedure is an extensor mechanism realignment of the knee combining lateral release, vastus medialis obliquus muscle advancement, and transfer of the medial third of the patellar tendon to the medial collateral ligament. The patients were regularly followed-up postoperatively at 2, 4, 8, 12, and 24 weeks, and then annually. Evaluation included the modified Cincinnati and the Kujala scores, anthropometry, plain radiography, and isokinetic dynamometry.

**Results** The average follow-up was 3.8 years (2.5–6). The mean modified Cincinnati score increased from 51.7 to 94.3 ($p < 0.02$), while the mean Kujala scores increased from 52.4 to 93.8 ($p < 0.02$). The muscle volume of the thigh of the operated limb increased with time, but remained significantly less well developed than the muscle volume of the thigh of the nonoperated limb ($p = 0.03$). The Insall-Salvati index remained essentially unchanged, being 1.04 preoperatively and 1.02 at latest follow-up. Significant differences were found between the operated and the contralateral limb in the various isokinetic strength variables at all angular velocities measured at the latest follow-up ($0.05 < p < 0.0042$). Only 1 patient suffered a patellar redislocation.

**Discussion** The minimum follow-up of 2.5 years and until skeletal maturation is long enough to consider that the results of surgery had stabilized. In a prospective randomized study, Palmu et al. observed that most of the first redislocations occurred within 2 years after surgery. Side-to-side differences in isokinetic strength and in anthropometric indices persist despite subjective success of the procedure. It is possible that heavy resistance training may have to be implemented to overcome these findings. Correct indications and a careful preoperative evaluation are of fundamental importance for successful long-term outcome. The 3-in-1 procedure does not preclude further surgical stabilization, as do the Elmslie-Trillat procedure or reconstruction of the medial patellofemoral ligament, when failure occurs after skeletal maturity.

**Conclusions** The 3-in-1 procedure is a safe, reliable management option for recurrent patellar dislocation in skeletally immature patients. Long-term evaluation is necessary, particularly to monitor the possible development of patellofemoral osteoarthritis.

### Return to sports activity after unicompartimental knee arthroplasty

M. Lo Presti*, F. Iacono, D. Bruni, M.P. Neri, G. Raspugli, M. Marcacci

Istituto Ortopedico Rizzoli (Bologna, IT)

**Introduction** There is continuing debate regarding the best treatment for young patients with unicompartimental osteoarthritis of the knee. In recent years, unicompartimental knee arthroplasty (UKA) has increased in popularity.

**Materials and methods** The authors surveyed 53 patients (range 46–66 years; mean age 59.79 years at review) by questionnaires to determine their sporting and recreational activities at a mean follow-up of 24 ± 6 months (range 12–48) after unicompartimental knee arthroplasty (De Puy International Ltd., with a cemented all- poly tibial component). We also assessed the state of general health (SF36) and all these patients had been reviewed using the Hospital for Special Surgery (HSS) score. For SF36 and HSS, patients were divided into groups of women (38) and men (15).

**Results** Before surgery, 46 out of 53 patients (87%) were engaged in an average of 5.0 sports and recreational disciplines (tennis, football, cycling, swimming, jogging); 49 (92%) participated in an average of 3.1 different sports disciplines (tennis, cycling, swimming); resulting in a return to activity rate of 95%. The most common activities after surgery were cycling in 14 patients (26.4%), swimming in 12 patients (22.6%), dancing in 6 patients (11.3%), jogging in 5 patients (9.4%). Different high-impact sports, such as tennis in 5 patients (9.4%), football in 5 patients (9.4%) and mountain climbing in 3 patients (5.6%), revealed a significant decrease in participation as well as the winter disciplines of downhill in 2 patients (3.7%) and cross country skiing in 1 patient (1.8%). Five patients were able to resume recreational football player: compulsive postoperative rehabilitation with restoration of muscular control was important for optimum function and for return to this activity.

**Discussion** The majority of patients who underwent unicompartimental knee replacement surgery were very satisfied with their quality of life, and SF36 score also improved significantly from pre-operatory after surgery ($p < 0.0001$).

**Conclusions** A modern UKR may be considered a valid alternative to bridge the gap between a HTO (hight tibial osteotomy) and TKR (total knee replacement) for young active patients with isolated unicompartmental tibiofemoral non inflammatory disease. Most of the patients returned to sports and recreational activities after unicompartimental knee arthroplasty. The activities in which most patients participated were primarily low or mild impact such as cycling, swimming, fitness, golf. It is necessary a conclusion regarding UKR for football players.

### Medial pivot prosthesis in valgus knee

G. Bracci*, F. Trentani, G. Trisolino, E. Gozzi, E. Martucci

Istituto Ortopedico Rizzoli (Bologna, IT)

**Introduction** Total knee arthroplasty (TKA) in valgus knee is more difficult than in well aligned or varus knee, The correction of deformities and ligament balancing are the priority. The aim of this study was to evaluate our experience on 20 knees treated with Advance TKA.

**Materials and methods** The study group is based on 20 consecutive patients who underwent primary TKA using cemented prosthesis fixation and a medial pivot implant. Study participants included 14 women and 6 men with a mean age of 69.6 years (range, 60–82 years). Osteoarthritis (80%) was the most common diagnosis rheumatoid arthritis (16%), and other findings (4%). All patients received the Advance TKA (cruciate ligament sacrificing) prosthesis (Wright) using cemented fixation.

**Results** Twelve knees were rated excellent, 8 as good. The femoro-tibial mechanical axis was corrected from a valgus of 15° to 5° in post-operative. There was no varus-valgus instability in 18 knees, and 2 knees had approximately 5° varus-valgus instability in extension. There was palpable, nonpainful, patellofemoral crepitus noted in 1 knee.

**Discussion** The present study reports our experience on a total cohort of 20 knees using Advance medial pivot system in primary knee arthroplasty in valgus knee. This type of prosthesis imitates knee anatomy and biomechanics. Medial pivot and "ball in a socket" design provide great stability to the implant.

**Conclusions** The Advance TKA (with cruciate ligament sacrificing) prosthesis (Wright) with cemented fixation was successful in primary arthroplasty in valgus knees, also in severe deformities with incompetent medial collateral ligament.

### *AT19 – PAEDIATRIC ORTHOPAEDICS*

### Surgical treatment of fractures of the lateral condyle of humerus in children

R.M. Toniolo*[1], A. Di Lazzaro[1], S. Rivelli[1], V. Guzzanti[2]

[1]Ospedale Pediatrico Bambino Gesù (Rome, IT);
[2]Ospedale Pediatrico Bambino Gesù, Università degli Studi di Cassino (Cassino, Rome, IT)

**Introduction** Fractures of the lateral condyle of the humerus account for approximately 12% of elbow fractures in children. Maintenance or surgical reconstruction of the articular surface is a prerequisite for healing without complications, although literature describes poor outcomes even with a good reduction and stabilization of the fracture (nonunion, osteonecrosis, axial or fish tail deformity, overgrowth of the lateral condyle). The aim of our study is to propose a treatment consisting of open reduction and suture fixation of the fragments of this kind of in fracture.
**Materials and methods** From January 1999 to May 2010 we treated 53 children with lateral condyle fracture of the humerus, 33 males (62%) 20 females (38%), the average age was six years and two months. 5 fractures were Milch 1 type and 48 Milch 2 type. In some cases there were associated lesions: in 2 cases elbow dislocation, in 2 other cases ulna fracture, in one case medial condyle fracture. We excluded from evaluation the fractures treated only with cast and those treated with open reduction and stabilization with a screw (10 cases). The open reduction and fixation with trans-osseous slow resorbable suture material was performed in 5 type I unstable fractures, 12 in type II fractures and 17 in type III fractures.
**Results** 29 patients were checked on follow-up (min. 12 months, max. 10 years). In all, there was a good consolidation after plaster removal without any secondary displacement. In four cases a deficit of flexion or extension (max. 15°) was observed, with no subjective functional problems reported. Any limitation of pronation and supination was observed. A constant radiographic finding was the overgrowth of the metaphyseal region proximally to the lateral condyle, independently from the type of fracture. In 8 patients the overgrowth took the appearance of a true bony prominence, clinically expressed as pseudo-varus elbow. In two cases there was axial deviation, one of which was associated with necrosis of the trochlea. In one case we found the presence of a calcification radio graphically visible in the periarticular soft tissues.
**Conclusions** The results obtained allow us to state that the technique of trans-osseous suture, if properly executed, has, over other proposed methods, several advantages: minor surgical aggression, minimal soft tissue dissection to avoid damage to the vascularity of the fragment and to minimize the risk of periarticular calcifications and no need for removal of internal bone fixation devices.

### The relationship between the growth of the acetabular lateral cartilage and developmental dysplasia of the hip: a histological study

N. Portinaro, A. Panou*, S. Camurri, M. Mori

(Milan, IT)

**Introduction** It is known that the triradiate cartilage contributes significantly to enlarge the acetabular diameter proportionally to the growth of the femoral head, while the ring epiphysis enhances final acetabular depth. The function of these growth cartilages in normal and pathological hips were described but their microanatomy was not depicted in detail.
**Materials and methods** The pelvis of a healthy child without any orthopedic disease, died in a car accident, was investigated after ethical approval was obtained. The acetabulum was radially dissected with a microtome and each section was immediately fixed in a paraformaldeyde bath for 48 h and subsequently decalcified, dehydrated and then stained with Hematoxylin and Eosin, Giemsa and Toluidine Blue for histological and immunohistochemical analysis.
**Results** Our study showed the presence of multiple inclusions in the resting zone of each acetabular growth area, in the center of the acetabular cavity, in the periphery of the acetabulum and in the flanges of the tri-radiate cartilage. These inclusions varied in number and position in the three acetabular bones.
**Discussion** Although there have been previous descriptions of histologic acetabular anatomy, the role of the inclusions described here in the resting zone of the acetabular growth centers has not been clarified in detail. We suggest that these inclusions may represent, as in other chondro-epiphysis, sites for development of secondary preossific nuclei from which subsequent ossification centres will mature. Our data do not allow to completely quantify the number and position of these nuclei as suggested by the different geographical distribution of these inclusions about the three acetabular bones.
**Conclusions** In DDH there is always a morphological alteration of the lateral acetabular growing cartilage. If associated with a definitive lesion of these possible precursors of the secondary ossification centres, caused at any time of growth, the definitive acetabular maturation could be geopardized. This would explain the late evidence of an altered hip joint development in even apparently normal hip after treatment for DDH.

### Treatment of paediatric flexible flatfoot with calcaneo-stop: clinical and radiographic findings at a minimum five-year follow-up

A. Marmotti*, M. Germano, A. Tellini, A. Tron, M. Cravino, L. Marengo, R. Del Din, F. Castoldi

Università di Torino (Turin, IT)

**Introduction** Flexible flatfoot in children is one of the most common disorders in paediatric orthopaedic practice. In a small percentage of cases (less than 10%) symptoms will develop in middle-late childhood (from 8–9 years) as pain in the hindfoot and in the medial midfoot, and foot and lower limb fatigue, more often related to sports activities. When conservative measurements fail to relieve the pain in symptomatic children, joint-preserving deformity-correcting surgery might be considered in patients between eight and fourteen years. We propose the extra-articular subtalar arthroereisis ("calcaneo-stop" with STJ-SAMO device) in conjunction with soft tissue procedures as spring ligament plication (medial plication, according to Pisani), posterior tibial tendon tensioning repair, and percutaneous Achilles tendon lengthening if needed.
**Materials and methods** A prospective study was performed including 58 feet treated between January 2000 and December 2006. Preoperative and post-operative examinations included: clinical evaluation, podoscopic examination, AOFAS-outcome-scale, standing X-rays. Foot types were classified as calcaneo-valgus, flat-valgus and cavus-valgus. Calcaneo-valgus feet were treated by isolated extra-articular subtalar arthroereisis, while in flat-valgus and cavus-valgus we performed subtalar arthroereisis in conjunction with medial soft tissue procedures and, when needed by decreased ankle dorsiflexion in subtalar neutral position, percutaneous Achilles tendon lengthening.

**Results** At a minimum 5-year follow-up, no serious adverse events occurred. Heel-valgus and the longitudinal arch of the foot improved clinically without any loss of function. There were no cases of hypercorrection. 6 feet underwent implant removal. The average AOFAS-Hindfoot-scale improved from 81.66 to 97.63, and the average AOFAS-Midfoot-scale improved from 87.39 to 97.63. The patients who played sport before surgery, returned to unrestricted sports. Radiological results show Meary's line inferior to 10° in all patients (mean angulation of Meary's line was 12.8° (SD 4.1°) pre-operatively and 1.8° (SD 1.7°) at the time of follow-up.

**Discussion** Hindfoot correction might be the result of a direct mechanical activity of the screw in limiting calcanear eversion, or in externally stimulating the proprioceptive foot receptors allowing active inversion of the foot or by the combination of all these factors. Tensioning of medial structures and Achilles tendon lengthening are accessory procedures for selected patients presenting with significant medial laxity and decreased ankle dorsiflexion in subtalar neutral position.

**Conclusions** Extra-articular subtalar arthroereisis with STJ device, in conjunction with soft tissue procedures, allowed effective correction of flatfoot in symptomatic patients, with pain relief and return to sport activities. This technique is simple and less invasive than other procedures as calcaneal osteotomies.

### Ischial tuberosity avulsion in the young

M. Ceretti*, S. Pappalardo

Policlinico Umberto I (Rome, IT)

**Introduction** Ischial tuberosity avulsion in the young is a rare pathology that is due to ischiatic growth nucleus avulsion from the remnant ischium. This pathology has the maximum incidence in young athletes between 15 and 18 years and its pathogenesis lie in a strong thigh posterior muscles contraction during sport exercises. Differential diagnosis with Hamstring muscle–tendon damage is the first cause of misdiagnosis.

**Materials and methods** We analysed a 15-year-old athlete case of ischiatic tuberosity avulsion following a football match trauma. The patient had an intensive oedema located on the buttock with associated pain, lameness, and ROM reduction with knee locked in extension. The ischiatic tuberosity avulsion was confirmed lately by TC and had specified fragment sizes as follow: diameter 44.4 mm, depth 10 mm, fragment diastases 14.37 mm. The conservative treatment (defined by Boyd et all. study 1997) for ischial tuberosity avulsion with fragment diastases lower than 2 cm was based on the following therapeutic planning: functional rest for 25 days with following physiotherapy focused on stretching exercises, connectival massages, Mesier airway exercises, exercises of progressive knee, pelvis e trunk stabilization, firstly out of load then static load and finally dynamic load at different degrees.

**Results** Three months after trauma the patient had recovered biological consolidation and full articular range of motion in both sides and was pain free during all soft and strong body exercises. After 1 year the patient had a stable clinical picture and was symptoms free.

**Discussion** One year later the we discuss about indication and therapeutic choices.

**Conclusions** The conservative treatment for ischial tuberosity avulsion with fragment diastases lower than 2 cm guarantees results as better as diagnosis is earlier. Physiotherapy has not to be focused only on damaged limb but also on the other side because ischial tuberosity avulsion is only a muscular retraction clinical sign. A correct sportive education about posterior limb stretching exercises could be a valid instrument to reduce the incidence of hamstring retraction. This one is the substratum which in adding of a strong contraction produces muscular or skeletal damage. In conclusion ischial tuberosity avulsions with fragment diastases lower than 2 cm treated conservatively gives good clinical and functional results.

### Arthroscopic osteoplasty for slipped capital femoral epiphysis

N. Santori*[1], A. Bertino[1], V. Arceri[2], E Theodorakis[2], D. Potestio[3]

[1]Casa di Cura Quisisana (Rome, IT);
[2]Policlinico Umberto I (Rome, IT);
[3]Casa di Cura Villa del Rosario (Rome, IT)

**Introduction** Patients with severe (SCFE) develop osteoarthritis earlier in life in association with mechanical impingement. Therefore, these patients are candidate for open surgical treatment. If the slippage is minimal to moderate, pinning of the proximal femur is the most common approach. However, this "conservative approach" may still result in some degrees of deformity and subsequent impingement. Therefore, we started treating patients with mild (0–30 degrees) slippage and positive signs of impingement with arthroscopic osteoplasty at the time of pin removal. Our hypothesis is that the avoidance of anterior impingement at an early stage can prevent the development of osteoarthritis.

**Materials and methods** Between January 2008 and January 2010, three male patients (average age 14.8 months) were treated with femoral arthroscopic osteoplasty. All patients were pain free before arthroscopy but presented limited motion and a positive impingement test. Epiphyseal-metaphyseal offsets and alpha angles were recorded.

**Results** At last follow-up, the three patients were pain-free and none demonstrated clinical impingement. All patients had a significant increase of R.O.M.

**Discussion** We had no complications in this series.

**Conclusions** Arthroscopic osteoplasty at the time of hardware removal can reduce impingement after mild slipped capital femoral epiphysis. Due to short follow-up, we are unable to state that this procedure reduces subsequent articular damage. However, arthroscopic femoral osteoplasty is a minimal invasive procedure and restoration of neck-head offset and clinically normal joint motion are important achievements for the future on these young patients.

### Child's bone cysts treated with courrettage and filling with bank's bone: a 15 cases report

G.A. Vercio*[1], C. Vercio[1], G. Ribaudo[1], I. Guttadauro[1], L. Promenzio[2], M. Bisignani[2]

[1](Palermo, IT);
[2](Rome, IT)

**Introduction** By reviewing publications concerning the treatment of paediatric bone cystis, it should be noted the important role of filling bone lesions by bank bone. This study reviews, in light of obtained results, the outcome of treatment in 15 patients treated by surgery courrettage of the cystis and filling with bone bank, evaluating time of consolidation, risk of fracture and radiographic outcomes.

**Materials and methods** The examination of 15 patients with bone cystis located respectively on homerus (5), radio (2), femur (3), tibia (2), astragalus (1) and clavicle (1), processed by courrettage and filling with bone bank, with an average follow-up of 4 years. In 2 cases the diagnosis followed a fracture in the cystic site, in the remaining cases the response was occasional. The outcome was assessed radiographically (searching pathological fractures and retention of areas of bone loss) and clinically (generalized pain or deformity).

**Results** Our series highlights a good consolidation of the lesion in the whole sample with a complete obliteration of the cavity in 7–10 months with disappearance of the radiolucent areas.

**Conclusions** The results were compared with those reported in the literature confirming the validity of the technique.

### Histological study of the Osgood-Schlatter lesion: etiopathogenetic considerations

F. Falciglia[1], A. Poggiaroni*[1], M. Giordano[1], A.G. Aulisa[1], P. Savignoni[1], V. Guzzanti[2]

[1]Ospedale Pediatrico Bambino Gesù (Rome, IT);
[2]Ospedale Pediatrico Bambino Gesù, Università degli Studi di Cassino (Cassino, Rome, IT)

**Introduction** From histological, clinical and radiographic observations, various hypotheses have been advanced about the etiopathogenesis of the Osgood-Schlatter (O–S) lesion. To date, no study has reported the histological features of the various zones of the anterior tubercle of the tibia in the different stages of the O–S lesion. In order to determine the characteristics of these zones, we carried out histological evaluation of O–S lesions in patients undergoing surgical treatment in various stage of growth.

**Materials and methods** Specimens were taken from 13 patients with O–S lesion, four in the apophyseal stage and nine in the epiphyseal stage of anterior tibial tuberosity. Core biopsies were obtained using a Jamshidi needle prior to surgical fixation. Specimens were prepared and stained with hematoxylin and eosin and Masson's trichrome.

**Results** In the apophyseal stage, lesions were present in an altered fibrocartilage anterior to the ossification centre. Reparative tissues were also observed in the upper part of the secondary ossification centre. In the epiphyseal stage, varying degrees of reparative tissues were observed in the bed of the fragment of the secondary ossification centre. In 3 out of 9 patients a zone of lesion was observed within the fibrocartilage anterior to the ossification centre.

**Discussion** Our study documented that O–S lesion was present in the apophyseal stage in the fibrocartilage anterior to the secondary ossification centre. The same observation was made in the epiphyseal stage in association with different reparative tissues inside the ossicle and its bed, suggesting that the slippage of the patellar tendon insertion may be progressive and caused by pathological fibrocartilage. The various stages of repair described in previous histological studies within the secondary ossification center are to be considered as the result of a lesion occurred in an earlier stage of development, when the cartilage anterior to secondary ossification centre is not yet ossified.

**Conclusions** Considering the period of growth when O–S lesion more frequently appears, the cause of this cartilage weakness may be similar to that observed in slipped capital femoral epiphyses. This would lead us to consider this disease as a progressive slippage of the patellar tendon insertion within a pathological cartilage anterior to the secondary ossification centre of the anterior tibial tubercle.

### *AT20 – MISCELLANY*

### Use of prolonged peripheral neural blockade after lower extremity amputation: the effect on symptoms associated with phantom limb syndrome

N. Fabbri, A. Tognù, R. Borghi, B. Borghi*, M. Mercuri

Istituto Ortopedico Rizzoli (Bologna, IT)

**Introduction** Phantom limb syndrome (PLS) is basically the most common and severe complication after limb amputation, occurring in more than 90% of amputees. Different medical, physical and psychological strategies have been used in this field, with overall quite unsatisfactory results. The efficacy of a prolonged perineural infusion of a high concentration of local anesthetic solution in preventing PLS has been evaluated in this study.

**Materials and methods** A perineural catheter was placed immediately before or during surgery in 71 patients undergoing lower extremity amputation. A continuous infusion of 0.5% ropivacaine was started intraoperatively at 5 mL/h using an elastomeric pump, and continued for 4 to 83 days after surgery. PLS was evaluated on the first postoperative day and then 1, 2, 3, and 4 weeks, and 3, 6, 9, and 12 months after surgery. The severity of phantom limb and stump pain was assessed using a 5-point verbal rating scale (VRS), with $0 =$ no pain to $4 =$ intolerable pain, and "phantom" sensations were recorded as present or absent. If the VRS score was $> 1$ or significant phantom sensations were present, the ropivacaine infusion was immediately restarted at 5 mL/h. If the VRS score remained at 0 to 1 and the patient had not experienced phantom sensations for 48 h, the infusion was permanently discontinued and the catheter was removed.

**Results** Median duration of the local anaesthetic infusion was 30 days (95% confidence interval, 25–30 days). On postoperative day 1, 73% of the patients complained of severe-to-intolerable pain (visual analog scale $> 2$). However, the incidence of severe-to-intolerable phantom limb pain was only 3% at the end of the 12-month evaluation period. At the end of the 12-month period, the percentage of patients with VRS pain scores were $0 = 84\%, 1 = 10\%, 2 = 3\%, 3 = 3\%$, and $4 =$ none. However, phantom limb sensations were present in 39% of patients at the end of the 12-month evaluation period. All patients were able to manage the elastomeric catheter infusion system at home.

**Discussion** This technique has been associated with superior results when compared to other techniques reported in the literature and previously used.

**Conclusions** Use of a prolonged postoperative perineural infusion of 0.5% ropivacaine seems to be an effective therapy for the treatment of phantom limb pain and sensations after lower extremity amputation and is currently the Authors' technique of choice.

### Atypical femoral fractures as a potential complication of long-term bisphosphonate therapy

G. Caruso*[1], A. Cervini[1], L. Viziello[1], P. Franceschetti[2], M.R. Ambrosio[2], L. Massari[1]

[1]U.O. di Ortopedia e Traumatologia, Azienda Ospedaliero Universitaria di Ferrara, (Ferrara, IT);
[2]Sezione di Endocrinologia, Azienda Ospedaliero Universitaria di Ferrara (Ferrara, IT)

**Introduction** A subtrochanteric fracture seems to be an unusual complication of long-term treatment with bisphosphonates, however, despite numerous reports in the literature, it was not shown a significant risk increase. Clinical and radiographic features characterize this association: location in the subtrochanteric region and femoral shaft, transverse or short oblique orientation, minimal or no associated trauma, medial spike when the fracture is complete, absence of comminution, cortical thickening, a periosteal reaction of the lateral cortex, prodromal pain, bilaterality, delayed healing, comorbid conditions, and concomitant drug exposures, including BPs, other antiresorptive agents, glucocorticoids, and proton pump inhibitors.
**Materials and methods** Ee analyzed retrospectively patients treated at our Clinic for subtrochanteric fracture from 2007 to 2010 with the aim of identifying those who had the radiographic features suggestive of atypical fracture among them we investigate the use of bisphosphonates. On 72 patients of mean age 81 years, 9 women with radiographic pattern suggestive of atypical fracture were identified, all were treated with bisphosphonates (on average 9 years). Among the 63 patients with fractures without atypical characters, it was found that seven were using bisphosphonates (average 2.7 years), 39 had never used bisphosphonates, while for 17 patients no data could be obtained.
**Results** Statistical analysis showed that in patients undergoing long-term treatment with bisphosphonates significantly increase the risk of atypical fractures (odds ratio $\gg 1$, $p < 0.02$). It also showed that the average duration of bisphosphonate therapy in patients who showed an unusual pattern of fracture (9 years) was significantly higher ($p \ll 0.05$) of the average duration therapy in patients treated with bisphosphonates, but with traditional X-ray pattern (2.7 years).
**Discussion** Although limited by a small sample, our data suggest that inhibition of bone turnover induced by prolonged use of bisphosphonates may increase the risk of atypical fractures of the femur in the subtrochanteric region. It remains unclear if simultaneous conditions can help to steer bone metabolism in this direction. The subsequent radiographic revaluation of the 9 patients with atypical fractures highlighted in the subtrochanteric region of the contralateral femur some of atypical characteristics as well.
**Conclusions** Without questioning the anti-fracturative role of these drugs in the treatment of osteoporosis, we believe that the risk of incurring an atypical fracture should be considered in patients undergoing long-term therapy with prodromal symptoms.

### Psychotropic drugs and hip fractures: sub-analysis of the INDACO 2 survey

G. Iolascon*, F. Gimigliano, M. D'Amico, R. Gimigliano

Seconda Università degli Studi di Napoli (Naples, IT)

**Introduction** Hip fracture is a devastating event in the life of an older person, as it often leads to loss of independence and death. The concomitant use of several medications for somatic and mental disorders is common in elderly people and increases the risk of falls and fractures. The use of CNS active drugs can impair the level of alertness and neuromuscular function. The aim of this study was to examine the association between the use of sedatives and antidepressants and the risk of hip fractures in a cohort of elderly people.
**Materials and methods** The study INDACO 2, an epidemiological survey promoted by the Italian Society of Orthopedics and Traumatology (SIOT), were collected data of 7,355 patients referring to 143 departments of Orthopaedics and Traumatology widely distributed throughout Italy over a 6-months period. It was created a form in order to investigate several aspects of patients' medical history, and evaluating the use of prior therapies, including psychotropic drugs (sedatives and antidepressants).
**Results** Of the 7,355 forms collected we excluded those who did not meet the inclusion criteria and therefore the final analysis was performed on 6,465 patients with a mean age of 76.9 years. The total number of patients who sustained a hip fracture was 2,902 (44.9%). Of the 6,465, 721 patients (11.15%) took sedatives, while 795 (29.12%) antidepressants. Of the 721 patients taking sedatives 439 (60.89%) had a hip fracture. Of the 795 subjects taking antidepressants, 409 (51.45%) had a hip fracture. The risk of sustaining a hip fracture was higher in both groups, with a odds ratio of 1.91 (95% CI: 1.63 to 2.23) for those using sedatives and 1.30 (95% CI: 1.12 to 1.50) for those using antidepressants.
**Discussion** The relationship between psychotropic medication and increased risk of falls in elderly subjects may be due to the impairment of postural stability, reaction time and sensory-motor functions. It is known that balance and attention significantly worsen even with small doses of drugs. The results of our epidemiological study confirmed that the use of psychotropic drugs increases the risk of hip fracture. This risk is significantly higher in those who make use of sedative drugs for a presumed increased risk of falling.
**Conclusions** The use of psychotropic drugs significantly increases the risk of hip fracture.

### Degenerative facet joint changes in lumbar percutaneous pedicle screw fixation: radiographic analysis of 30 patients

L. Proietti*, L. Scaramuzzo, S. Sessa, G.R. Schirò, C.A. Logroscino

Università Cattolica del Sacro Cuore (Rome, IT)

**Introduction** Aim of the study was to evaluate the appearance of degenerative lumbar facet joints changes due to percutaneous pedicle screw fixation for the treatment of lumbar fractures without neurological involvement.
**Materials and methods** Thirty patients affected by fractures of the lumbar spine underwent percutaneous pedicle screw fixation. In all patients a short fixation (4 screws) without fusion was performed. Radiographic analysis was performed by a thin cut CT scan in the pre-operative and post-operative time at 4 months in 10 patients, 8 months in 7 patients and 12 months in 13 patients. Facet joints degenerative alterations were classified according to Pathria criteria. In all patients the 4 facet joints adjacent the screws and these at fracture's level were evaluated. The facet joints encroachment due to screw misplaced was also evaluated.
**Results** Eleven patients presented an A3.1, 4 an A3.2, 15 an A.3 fracture. Patients were divided into 3 groups considering CT scan execution time: group A 4 months, group B 8 months, group C 12 months. Patients in the group A showed no statistically significant differences between pre and post-operative facet joints ($p > 0.05$). Patients in the group B showed a statistically significant worsening in the 28% of the facet joints at fracture's level ($p = 0.03$). Patients in the group C showed a plain degeneration in the 100% of the cases ($p = 0.001$). Considering screws placement there was an impingement with the articular surface in 6 cases, responsible of an early degeneration of the related facet joint. Seven patients were older than 65; 3 of them showed a complete degeneration of the facet joints at fracture's level comparable to a fusion yet to 8 months.
**Discussion** Results' analysis showed the presence of significant facet joints degeneration yet to 8 months after a percutaneous screw fixation. Facet joints fusion was observed only in patients older than 65 with a high pre-operative degeneration grade.

**Conclusions** In the authors' opinion lumbar fractures treatment with percutaneous screw fixation without fusion causes an early facet joints degeneration and has so not to be considered an harmless procedure. This study encourages to remove percutaneous pedicle screw instrumentation no later than 1 year to avoid severe facet joints degenerative changes.

### Hip and knee arthroplasty in Italy: temporal trends and gender analysis

V. Manno*, M. Masciocchi, M. Torre

Istituto Superiore di Sanità (Rome, IT)

**Introduction** In 2000 TKR was defined as the joint of the decade, a successful procedure for which there is a large unmet need. Thanks to the advances in both bioengineering technologies and surgical techniques, in 2007 THR was defined as the operation of the century.
**Materials and methods** The national Hospital Discharge Records Database (2001–2008) was browsed for the selected procedures in every of the six fields available. The total number of THR and TKR was computed. Hospitalizations rates (per 100.000) standardized for age and sex were compared for the years 2001 and 2008, the ratio between the two standardized rates (Relative Risk - RR, Poisson distribution) and the gender RR were computed.
**Results** In 2008, 60,835 THR and 56,642 TKR were performed in Italy. The average yearly increase was about 4% (THR) and 11% (TKR). From 2001 to 2008, age and gender standardized hospitalization rates increased from 79.54 to 99.58 (THR) and from 45.40 to 93.65 (TKR). Gender analysis of RR showed an excess of risk for women for both THR and TKR. For THR the excess of risk was 40% (RR = 1.40; C.I. 1.37– 1.43) in 2001 and 21% (RR = 1.21; C.I. 1.19–1.23) in 2008. For TKR the excess of risk was 144% (RR = 2.44; C.I. 2.37–2.51) in 2001 and 98% (RR = 1.98; C.I. 1.94–2.01) in 2008.
**Discussion** In the observed period, both interventions showed a significant increase in RR. Considering that 25% of the increase in RR can be related to the increase of the arthritis prevalence, the difference on the risk for TKR might be connected to a higher spread of the indication for intervention. As show in the literature, women have a significant higher risk to be operated than men.
**Conclusions** Trends of hospitalization rates are continuously increasing for both THR and TKR. TKR rate is significantly higher than THR rate. Women undergo THR and TKR significantly more than men.

### Open and arthroscopic arthroplasty of the STT joint

R. Luchetti*, R. Cozzolino, L. Pegoli

(Rimini, IT)

**Introduction** Osteoarthritis of the STT results in a limitation of wrist function characterized by pain and reduced range of motion of the wrist and hand grip strength. The alternative treatment to the arthrodesis is the arthroplasty of the STT which consists in the resection of the distal scaphoid. This procedure can be performed with both surgical and arthroscopic technique.
**Materials and methods** From 2000 to 2009, 20 cases were operated on. Twelve of them were operated by arthroscopical technique. The open surgery group consists of only females, with a mean age of 63 years (48–76 years). In the arthroscopic group, 9 cases were females: the mean age of this group was 65 years (63–67 years). All the cases were degenerative but one. All cases were clinically evaluated preop and postoperatively with the Mayo Wrist Score and all were given the DASH questionnaire. X-rays of the wrist were taken before and after surgery and in all cases the presence of a DISI deformity of the lunate was evaluated.
**Results** There were no significant complications in the operated cases. With a mean follow-up period of 46 months (12–112 months) in the surgery group the pain improved without reaching statistically significant values. The wrist ROM and grip strength were essentially unchanged. At a median follow-up period of 24 months (14–48 months), in the arthroscopic group, pain improved with statistical significance ($p > 0.001$), wrist ROM improved and grip strength unchanged. The DASH questionnaire score showed an improvement from 78 to 25. The radiological study showed the appearance of a DISI deformity in 4 cases of open surgery and in 3 cases of arthroscopic treatment.
**Discussion** There is no substantial clinical and radiological difference between the two techniques. Even if the DISI deformity is present, patients were painless and satisfied with the result being able to return to the same previous work. However, it was quite evident an earlier recovery in the cases operated by arthroscopy than by open surgery.
**Conclusions** Resection of the distal pole of the scaphoid for osteoarthritis of the STT seems to be an efficient method of treatment achievable by both open and arthroscopic surgery. Both techniques allow the achievement of good functional results with decrease of pain and recovery of the motility of the wrist and hand grip strength.

### The reimbursement model adopted to feed the Apulian Arthroplasty Registry results after 1 year since its introduction

M.T. Balducci*[1], M. Torre[2], D. Carbonara[3], S. Mudoni[1], D. Parisi[1], V. Manno[2], M. Masciocchi[2], C. Germinario[1], Gruppo di Lavoro Ortopedici Pugliesi[4]

[1]Osservatorio Epidemiologico Regione Puglia (Bari, IT);
[2]Ufficio di Statistica Cnesps, Istituto Superiore di Sanità (Rome, IT);
[3]Scuola di Specializzazione in Igiene e Medicina Preventiva, Università degli Studi di Bari "Aldo Moro" (Bari, IT);
[4]Regione Puglia, IT

**Introduction** Since 2003, the Epidemiological Observatory of the Puglia Region (OER) has maintained the Arthroplasty Registry (RIPO). The continuous increase in the number of primary and revision procedures and of the related health care expenditures made necessary to adopt post-marketing control systems to monitor over time the implanted devices and ensure the patients' traceability. Since 2007, the RIPO has actively participated in the National Registry project coordinated by the Italian National Institute of Health (ISS). The Registry is a prospective observational study on a large scale, aimed to collect a few essential information on all the performed arthroplasties with the single endpoint of detecting the implant failure (revision).
**Materials and methods** In 2010, to strengthen and implement the results obtained until then, Apulia Region established the Arthroplasty Regional Registry (Regional Law N.4 of 25 February 2010, Article 40) stating that all the public and accredited hospitals performing hip or knee and/or shoulder arthroplasties were required to fill in a specific paper data collection form. The same regulation established that the reimbursement of the intervention would be provided by the region to the hospital only if the data collection form has been duly filled in.

**Results** Apulia is one of the three regions in Italy currently running a regional registry. However, the number of reports (compliance), equal to 70% in 2003, fell to 16% in 2009. In 2010, being the surveillance system changed from voluntary to reimbursement based, participation of 100% of Apulia hospitals was registered (compliance 95%). In 2010, 7,274 arthroplasties were recorded (hip: 4,050; knee: 3,074; shoulder, 150) performed in 52 orthopaedic units of 32 public hospitals, 2 university hospitals, 2 ecclesiastical bodies, 1 IRCCS and 10 private hospitals. 37 different cups and 53 stems were identified. 28 mm femoral heads were used in 90% of implants. A higher prevalence of coxarthrosis on the right side was measured (56.3% *vs.* 43.7% on the left).

**Discussion** The dramatic increase of the compliance (+69% in 1 year) showed that, for the validity of the registry, rules that make compulsory the registration must be adopted. Therefore it is desirable that, following the Apulia example, these rules should be adopted at both national and regional levels.

**Conclusions** The adopted model will contribute to the implementation of the National Arthroplasty Registry, tracking the implanted devices and monitoring their quality, a prerequisite for undertaking appropriate health policies.

### ORAL COMMUNICATIONS

### *C49 – HIP 5*

### Use of two different hyaluronic acids (concentration/molecular weight) in the hip osteoarthritis, with the support of an echographic device

P. Bertocco*, G. Giaffreda, D. Clementi

Rho-Milano, IT

**Introduction** The first step of a hip osteoarthritis (HO) is an overweight for the joint, with loosing properties of the cartilagineous set. The pain is generally the first clinical evidence, with a secondary functional impairment. An important functional damage could be shown when a great part of the cartilagineous structure is involved. The pattern of the cartilagineous width is detected by X-ray score of Kellgren-Lawrence (KL). The hyaluronic acid (HA) seems to have a good viscosupplementation action for higher molecular weight. The use of HA could have a role in pain control and function improvement of the hip joint, quite preserving the cartilagineous structure by the damage.

**Materials and methods** Hip Osteoarthritis; KL 2-3. Hip infiltration using an ultrasound device (7.5–10 MHz); spinal needle (90 mm, 18 G). Two groups (G1; G2), 6 patients each; follow-up 6 months. G1: HA 30 mg, n° 4 infiltrations G2: HA 60 mg, n° 3 infiltrations. At each control, the following data were collected: ROMs, VAS, Oxford Hip Score (OHS), Harris Hip Score (HHS), Lequesne Index (LI), SF-36.

**Results** VAS decreases in each group, for G2 > G1 (61 to 41, final). OHS increases (functional improvement) in G2 (27 to 37, final), lesser in G1 (33 final). IL showed results quite similar to OHS. At the 6th month G2 *vs.* G1: + 25%. SF-36 and HHS did not show relevant variations. IL and OHS, when tested *vs.* ROMS, showed an association with extra-rotation and abduction.

**Discussion** Pain decrease, higher in G2, could be correlated to a different HA viscosity degree. The follow-up could better be tested by OHS and LI than HHS and SF-36, with ROMs assessing, too. These scores seem to be able to assess the difference between the two groups in terms of efficacy/no efficacy of the HA therapy. No sign of collateral effects was registered.

**Conclusions** The use of a higher molecular weight of HA in HO seems to be related to a better pain control and functional improvement. An ultrasound device is a non invasive technique with a quite good accuracy, which is be able to better define the hip anatomy in order to follow the needle through the tissues up to the joint line, without X-ray exposure.

### The volume surgeon and the frequency of endoprostesis, total hip arthroplasty and revision prosthesis dislocation

M. Marinelli*, D. Enea, S. Hassan, L. De Palma

Cattedra di Ortopedia e Traumatologia, Università Politecnica delle Marche, Azienda Ospedaliero-Universitaria, Ospedali Riuniti (Ancona, IT)

**Introduction** Hip dislocation has long been one of the major complications after hemiarthroplasty (HA), total hip arthroplasty (THA)

and revision prosthesis (RP). The purpose of this study is to report the incidence of dislocation after HA, THA and RP and analyze the factors involved, with particular emphasis to the concept of the volume surgeon.

**Materials and methods** 561 hips received either HA(202), THA (279) and RP (80). Of the 561 prosthesis, 31 sustained early or late dislocations one or more times. The associated database was reviewed for the occurrence of subsequent procedures involving closed or open reduction of the hip joint for dislocation of the index HA, THA or RP procedure. The factors to be studied included age, sex, original diagnosis, type of prosthesis, frequency of dislocation, the surgeon and hospital volume.

**Results** Thirty-one hips experienced dislocation with an overall dislocation rate of 5.5%. The rate of dislocation was 3.6% in HA (7/202), 5.3% in THA (15/279) and 11.2% in RP (9/80). There were 8 men and 23 women with an average age of 78.6 (45-96), with no significant difference in the dislocation rate between them. The year hospital volume in hip surgery was 112.2, and the annual volume surgeon was 18.7. In our experience, the dislocation rate was not related to sex, age, previous revision surgery, or types of prosthesis. The rate of recurrent dislocation was not related to a history of previous surgery.

**Discussion** The strengths of our study include the recent time period reflecting contemporary implants. Moreover, the same surgeons made the surgical intervention of THA, HA and revision surgery. Considered as a whole, we could support the idea of a positive association between surgical volumes and improvement in most arthroplasties outcomes, including dislocations. This correlation is more clearly established for surgeon volumes than it is for hospital volumes.

### Access road in anterior minimally invasive hip replacement surgery with the use of Maquet device for limb traction: our experience

L. Ciriaco*, M. Ferlazzo, A. Evanghelu

Casa di Cura COT (Messina, IT)

**Introduction** We analyze the experience resulting from the first three years of using the anterior access to the hip with the adoption of Maquet bed and a traction device.

**Materials and methods** An analysis of 64 procedures performed with this technique was carried out comparing it with 60 procedures performed with anterolateral access (the traditional approach). We took into account the following parameters: (1) surgical time; (2) blood loss; (3) number of staff needed during surgery; (4) clinical and X-ray charts.

**Results** We observed a substantial alignment in surgical times, a slight reduction in blood loss and a net improvement in the immediate clinical results with a tendency to an alignment after two years.

**Discussion** Given the increasingly felt need of a real miniinvasivity it was decided to take the route of direct anterior approach. This technique allows approaching the hip without any muscle detachment. However, it requires a certain adjustment period, thus the learning curve is quite long. The integration with the traction device of the Maquet surgical bed allowed us to optimize the gestures in the small surgical field available to the acetabulum at first and then to the femoral neck. The use of short stem prosthesis amplified the benefits of this access road. We believe that the good short-term results are directly linked to the greater compliance of the muscles and soft tissues.

**Conclusions** The Surgical approach is difficult, the surgical time is equivalent although we detected less blood loss. Good results in the short term. These considerations lead us to continue experimenting with this access.

### Cross-cultural adaptation and validation of the Italian version of the HOOS (hip disability and osteoarthritis outcome score) questionnaire

M. Torre*[1], M. Masciocchi[1], V. Manno[1], G. Tucci[2], V. Amorese[2], E. Romanini[3], G. Zanoli[4], R. Laforgia[5], L. Zagra[6]

[1]Istituto Superiore di Sanità (Rome, IT);
[2]Ospedale "Spolverini" (Ariccia-Rome, IT);
[3]Artrogruppo (Rome, IT);
[4]Casa di Cura S.M. Maddalena (Rovigo, IT);
[5]Clinica S. Rita (Bari, IT);
[6]IRCCS Galeazzi (Milan, IT)

**Introduction** More than 20 different scores are currently available to evaluate the results of total hip replacement (THR), most of them have not been still validated in the original version or at least in Italian. In the last years, the "objective" assessment of the patient has been gradually substituted by scores intended to evaluate the outcome from the patient's point of view (subjective scales). In 2003, starting from KOOS questionnaire developed for knee, the HOOS score was developed to assess hip function.

**Materials and methods** An observational prospective multicentric study including 5 orthopaedic departments and coordinated by the Italian National Institute of Health has been carried out. In the first phase the questionnaire was translated by two independent translators with a different cultural background. After reconciliation, the text was back-translated in English by an American native speaker without any specific background in the health field and blinded to the original English version. The reviewed and harmonized text together with the SF-12 standard and the EQ-5D questionnaires and the VAS scale was then administered to 96 patients undergoing THR (pre-op.) and then 6 months after surgery (follow-up). The first 10 patients recruited were also requested to fill in the questionnaires after 20 days to test its reliability.

**Results** The results of the administration of the HOOS questionnaire are presented. 87 patients filled in the pre-op. questionnaires properly. 65% of them were women (mean age 64.4 range 36–80) and 35% men (mean age, 64.4; range, 34–83). 80% of patients completed the follow-up questionnaires, enough to proceed in the validation of the score.

**Discussion** In this study the English version of the HOOS was translated and cultural adapted to Italian. Feasibility, internal consistency, reliability, construct validity and responsiveness were assessed as it should be done for every tool before its introduction into the clinical practice.

**Conclusions** The Italian version of the HOOS score for patients affected by hip osteoarthritis undergoing THR was demonstrated to be valid for patient centered outcome assessment. This study confirms that the Italian version of HOOS is a tool that can be adopted in clinical studies aiming at assessing outcomes after hip surgical procedures.

### Clinical preliminary results of the COLLO-MIS neck preserving short stem

E. Heijens*, M. Krieger, N. Kuhn

Gelenkzentrum (Wiesbaden, DE)

**Introduction** The COLLO-MIS short stem was designed to preserve the bone stock of the proximal femur and the femoral neck. This design combined with the surface texture permitts the rearrangement of an optimal biomechanical balance being essential to provide primary and secondary stability.
The aim of the study is to describe clinical and radiographic results of the COLLO-MIS short stem.
**Materials and methods** Between November 2008 and September 2010 we treated 401 patients with a total hip replacement implanting a short stem type COLLO-MIS (Limacorporate, San Daniele, Udine). Four surgeons operated using a minimally invasive surgery (MIS) technique with an anterolateral approach. The common data are presented as a clinical case report. For the first group including 102 patients (55 women and 47 men, mean age 65 years) the study was designed as a prospective surveillance study with a follow up period of 12 months. The underlying pathology was primary coxarthrosis (90), dysplasia (9) and necrosis of the femoral head (3).
**Results** The average Harris hip score improved from 48 before to 97 at 1 year follow-up. Preoperatively 95% of the patients had moderate or severe pain whereas at first follow-up there was either no or only slight pain in 95% of the cases. No patient had significant thigh pain and no limp could be observed. The WOMAC showed excellent satisfaction scores. In the 401 patients there were 2 stem relied complications. In both cases the mechanical loosening was due to intraoperative under sizing.
**Conclusions** With regard to the satisfactory clinical short-term results, short rehabillitation period and low failure rate this device seems promising. The intraoperative achieved primary stability is consolidated by a good osteointegration. However long-term follow-ups are necessary to confirm the results and to evaluate the durability of the implant.

### *C50 – HIP 6*

### 242 cups in Trabecular Titanium: short-term follow-up

L. Perticarini*, L. Piovani, S.M.P. Rossi, A. Combi, A. Padolino, F. Benazzo

Clinica Ortopedica e Traumatologica, Fondazione IRCCS Policlinico San Matteo (Pavia, IT)

**Introduction** Trabecular Titanium (TT) is an alveolar structure composed by a plurality of 3D complex shape hexagonal cells, with pores average diameter by 640 μM, such as to ensure good osseointegration without modify the mechanical strength of titanium. The low rigidity of the structure of the TT favors the transmission of loads from the implant to bone.
**Materials and methods** From September 2007 in Department of Orthopaedics and Traumatology of Pavia were implanted 242 Delta TT cup: 23 in 2007, 71 in 2008, 50 in 2009, 77 in 2010 and 21 in 2011. 117 cases the hip prosthesis was the right and 97 cases the left, while in 14 cases, the implant was bilateral. The mean age of patients was 57.4 years (range 20–93 years). In 228 cases the cup was used by first implant, while in 14 cases it was used by revision for aseptic loosening. In the first implant, the cup was applied in 146 cases of primary hip osteoarthritis, in 41 cases of DCA, in 20 cases of aseptic necrosis of the femoral head in subcapital fractures of the femorale, in 10 cases of post-traumatic arthritis, in 1 case the outcome of slipped capital femoral epiphysis and in 1 case of cut-out nail of femur nail. The cups size ranged from 44 mm to 62 mm. In 52 cases, screws were used to increase primary stability. The cups were associated with a straight stem type C2 (67), C2 lateralized (68), alloclassic (2) and with modular tapered stems modulus (93) according to the morphology of the femur. In six cases of revision surgery only the cup was replaced, and in other 6 cases a tapered revision stem was implanted. The average follow-up was 19.2 months, and the last follow-up was at 41 months.
**Results** The Harris Hip Score (HHS) pre-operative average was 40.8. At final follow-up HHS was 95.2. One cup was replaced 9 months after surgery for aseptic loosening. Twelve cases presented radiolucent lines areas around the cup (7 in Gruen Zone 1, 2 in Gruen Zone 2, 5 in Gruen Zone 3), which not exceed 2 mm and were stable in subsequent follow-up.
**Discussion** Trabecular Titanium showed high capacity of osseointegration, providing excellent results in short-medium term follow-up. Further studies and long-term follow-up to compare the clinical results of TT with those obtained with other cups have already been validated.

### A comparative analysis of the state of periprosthetic bone density in cementless prosthetic femoral components resurfaced with hydroxyapatite and spongy metal

F. Bove*[1], F. Volpato[1], G. Bove[2]

[1]Istituto Neurotraumatologico Italiano (Grottaferrata-Rome, IT); [2]Università "Sapienza" (Rome, IT)

**Introduction** Hydroxyapatite and spongy metal are two of the most frequently used resurfacing coatings for cementless prosthetic femoral components. The objective of this analysis is to compare the periprosthetic bone remodelling process of these two coatings.
**Materials and methods** About 1200 patients were operated between 2001 and 2010 (800 patients operated between 2001 and 2007 had prosthetic implants resurfaced with spongy metal, while 400 patients operated between 2007 and 2010 had their prostheses resurfaced with hydroxyapatite), clinically evaluated (by using the Harris score) and underwent DEXA (Hologic QDR 4500 W) densitometric analyses as well as periprosthetic (Gruen zone) analyses.
**Results** The densitometric analyses of the prostheses resurfaced with spongy metal showed bone density (BMD) to remain fixed over time in the Gruen zones 2, 3, 4, 5, and 6, while zones 1 and 7 (the greater trochanter and the calcar) indicated a tendency towards remodelling. The analyses of the prostheses resurfaced with hydroxyapatite indicated a post-operative steady remodelling trend which lasted up to 18 months and then stabilized without further relevant variations. The Harris score for both types of prosthetic implants was 90 on average at the final follow-up.
**Discussion** The results obtained by the DEXA analyses showed a tendency to proximal stress shielding for the prostheses resurfaced with spongy metal. On the other hand, the prostheses resurfaced with hydroxyapatite showed no variations at a metaphyseal level.
**Conclusions** The prosthetic stem resurfaced with spongy metal osteointegrates distally through a distal distribution of the burden of weight. The prostheses coated with hydroxyapatite showed a uniform osteointegration over time.

### The middle-term experience of a single center with 486 Conserve Plus® Wright hip resurfacing prostheses: 5.2-year mean follow-up

V. Bellotti*[1], R. Ledesma[1], C. Cardenas[1], D. Marinò[2], M. Ribas[1]

[1]Instituto Universitario Dexeus (Barcellona, Spain);
[2]Dipartimento di Ortopedia e Traumatologia, Università Federico II (Naples, IT)

**Introduction** The hip resurfacing protesis has lived a return in the last ten years, thanks to the possibility to re-establish the anatomy, to preserve the bone stock, and to offer some excellent functional results to middle term. We presents our experience matured in the resurfacing hip replacement of a single Department of Hip Surgery based on 486 interventions.
**Materials and methods** A series of 486 Plus® Wright hip resurfacings, performed between July 2003 and September 2008. The mean follow-up was 5.2 years (2–74). On a total of 450 patients, 386 were male. The mean age was of 46.6 years (16–69). A minimally back Kocher-Langenbeck approach was performed in 454 cases, anterior of Hueter in 32 cases. Osteoarthrosis on femoral-acetabular impingment was the diagnostic on 86% of the cases. The patients were evaluated with the Merle of Aubignè-Postel, Harris and WOMAC scales. The implants were evaluated with X-rays. Surgical time, bleeding, dimensions of the components and complications were recorded.
**Results** The average inclination of acetabular component was of 43.4° (35°–65°), the femoral one of 137.9° (127°–150°). The hospitalization and bleeding were greater in the cases operated by posterior approach. The surgical time was greater in the cases of anterior approach. At middle-follow up a notable improvement in all the clinical-functional scores was observed. The cases of revision were 10: one due to infection, one happened within the first three years of experience. The middle survival of this series is attested on 97.9%. Among the complications: 2 cases of TVP, 12 cases of disestesia of the lateal femorocutaneus nerve, 4 transitory paresis of the crural nerve, 11 ileopsoitis, 2 superficial hematomas.
**Discussion** We believe that resurfacing prosthesis represents a minimally invasive procedure, in terms of bone stock preservation, bleeding and hospitalization, in comparison to traditional implanta. Even though we have a limited experience, observed survival rate lead us to maintain this indication in the young adults patients with osteoarthosis.
**Conclusions** Hip resurfacing prosthesis technically represents a complex procedure with an important lerning curve. A suitable selection of patients, as well as a correct technical execution, allow to get satisfactory results with few complications.

### Mid-term results of a non cemented modular cup

A. Bistolfi*, F. Rosso, G. Massazza, F. Lagalla, F. Galetto, C. Olivero, M. Crova

Dipartimento di Ortopedia e Traumatologia e MDL, AO CTO/M. Adelaide, Università degli Studi di Torino (Turin, IT)

**Introduction** Acetabular cups made of a press-fit metal-back with morse taper for the insert joint liner (available in ceramic, metal or polyethylene) are widespread used. However, this kind of design was recently introduced and updated clinical follow-up studies, even in the short term, are necessary. The purpose of this study was to evaluate 207 acetabular cups delta type PF (Lima®) with a minimum follow-up of 3 years.
**Materials and methods** From 2004 to 2007, 207 Delta PF cups have been implanted in 190 patients (17 bilateral) with an average age of 61.7 years (SD ± 12.3); 82 men (43.2%) and 108 women (56.8%). The indications to surgery were: 161 idiopathic hip arthritis (77.8%), 31 dysplasia (DCA) (14.9%), 8 outcomes of necrosis (IOFH) (3.9%), 3 rheumatoid arthritis (1.5%) and 4 other diseases (1.9%). In 84 cases (40.6%) ceramic–ceramic coupling was used, while metal–metal in 93 cases (44.9%) and in 30 cases (14.5%) metal-polyethylene one. The cups were evaluated using the Harris Hip Score (HHS) and plan radiographs for signs of loosening.
**Results** 44 patients were lost to follow-up so 163 patients returned the study (85.9%, 178 cups) with an average follow-up of 49.7 months (SD ± 8.1), minimum 3 years. All HHS scores suffered a statistically significant increase from pre-operative: the total score increased from 55.5 (SD ± 5.7) to 94.7 (SD ± 3.4). 3 cases of failure (1.7%, two cases of loosening and one dislocation), the cumulative survival rate was 98.6% (SD ± 0.8%). The radiographic analysis was performed on 171 cups (96.1%): in 2 cases (1.2%) progressive radiolucent lines (loosening) were detected and in 20 cases (11.7%) heterotopic ossification were found.
**Discussion** The clinical and radiographic results are good, with a low failure rate and a high cumulative survival (98.6%). In addition, this acetabular cup has a good press-fit and the possibility of choosing the coupling according to the needs of the patient. In particular, it is suggested the use 36 mm femoral heads.
**Conclusions** The cup in study, at mid-term follow-up, provides good clinical and radiographic results. Since the implant was recently introduced but widespread used, longer follow-up is needed to confirm the results.

### Our experience with TRIBOFIT® Acetabular System

A. Berizzi*, P. Friso, A. Pozzuoli, R. Aldegheri

Clinica Ortopedica, Università (Padua, IT)

**Introduction** TRIBOFIT® Acetabular System is a polycarbonate-urethane buffer that can be applied as an insert in the metal back or in direct contact with bone. The characteristics of the polycarbonate-urethane are elasticity, low coefficient of friction coupled with metal surfaces, and large size of debris, exercising low action on macrophages activation. The buffer thickness (3 mm) allows the use of large diameter metal heads. The aim of our study is the evaluation of short-term results of a consecutive series of hip replacement with TRIBOFIT® Acetabular System applied directly to the acetabular bone.
**Materials and methods** Among the 54 hip replacements, the study included 45 patients (36 female, 9 male, mean age 81.2 years) based on the following criteria: aged 75 years, with non-pathological femoral neck fracture (Garden 3 or 4), physically active, self-sufficient, absence of osteoarthritis and acetabular dysplasia. The study provided patients reassessment at 6, 12 and 24 months with radiographic and clinical examination using SF-36, VAS, and HHS tests.
**Results** 38 patients completed the 6 and 12 months follow-up. After 6 months, the mean SF-36 score was 57.3, the VAS score was 4.3 and the HHS 74.2. After 1 year, the average SF-36 was 53.2, the VAS score 2 and the HHS 86. After 6 months, the HHS was excellent in 2 patients, good in 6, sufficient in 2 and poor in 5 patients. 4 patients died for causes independent of the arthroplasty.
**Discussion** We can take the following considerations: TRIBOFIT® Acetabular System allows the removal of "only" articular cartilage, implies a low invasiveness, a considerable saving of bone, and less blood loss.
**Conclusions** Our study shows that the use of TRIBOFIT® Acetabular System allow good results on short-term and it can represent a viable alternative to the partial arthroplasty.

### Polinucleotides versus ialuronic acid in ecoguided hip infiltration: preliminary results

A. Sanfilippo*[1], G. Letizia Mauro[2], A. Geraci[3], R. Sutera[4], A. D'Arienzo[1], M. D'Arienzo[1]

[1]Clinica Ortopedica, Università degli Studi (Palermo, IT);
[2]U.O.C. di Medicina Fisica e Riabilitativa, Università degli Studi (Palermo, IT);
[3]Ospedale di S. Maria del Prato (Feltre, IT);
[4]Istituto di Radiologia, Università degli Studi (Palermo, IT)

**Introduction** The ecoguided viscosupplementation has a large application in the treatment of hip osteoarthritis: consequently various drugs are suggested for intra-articular use, and among them, the Polinucleotides recently. Therefore the object of the study was to compare the efficacy between the gel of polideossiribonucleotides (PDRN) and the Hyaluronic Acid that is esteemed the "king drug" in the intra-articular infiltration and certainly is very good evaluted for his clinical effects.
**Materials and methods** We examined two homogeneous groups of 50 patients each with diagnosis of hip osteoarthritis valued group I-II in conformity with the Kellgren-Lawence's scale. Both the group advised to treatment with ecoguided viscosupplementation: in the first group (PDRN group) with a gel of Polinecleotides (Condrotide 2 ml. - 20 mg/mL), in the second group (HA group) Acid Hyaluronic-molecular high weight (Hyalubrix – 2 ml). All the patients of the two groups were infiltrated for 3 times, every 45–60 days.
**Results** The outcome measures were a 100 mm visual analogic scale (VAS) of the ankle pain, the Lequesne functional index and the drug consumption. These parameters are valued in the time of recruitment (T0), after 3 months the first intra-articular injection (Ti) and till the end of the examination (not before 6–12 months - Tf). The PN group and the HA group showed a mean symptomatic improvement from baseline (T0) with reduction of VAS (from 6.67 to 4.21 in the HA group and from 6.40 to 4.25 in the PDRN group), reduction of Laquesne index (from 11.87 to 8,98 in the HA group and from 11.57 to 9.08 in the PDRN group) and reduction of drug consumption (from 6,18 to 3,14 after 3 months and to 1,62 after 6-12 months in the HA group and from 5, 59 to 3,98 after 3 months and to 1, 77 after 6–12 months in the PDRN group). There were no differences in the incidence or nature of adverse events between groups.
**Conclusions** The data, clinically and statistically significant, can be considered superimposable in both groups and showed that polinucleotides and HA are important resources against the hip osteoarthritis because they reduce pain, improve function and mobility and reduce the drug consumption.

### *C51 – HIP 7*

### Total neck preserving prosthesis: 2-year follow-up

A. Camera*

Ospedale "Santa Corona" (Pietra Ligure-Savona, IT)

**Introduction** For the last two years we have been utilizing the Just stem as a total neck preserving prosthesis. The philosophy of the prosthesis foresees the substitution of the femoral head that is replaced by a curved mini-stem which reproduces the physiological forces of the femoral neck. There are no contraindications from femoral head osteonecrosis to Just prosthesis. The Just prosthesis is inserted into the trabecular bone system and follows Wolf's law by recreating physiological bone remodelling and benefiting from that same trabecular system for integration.
**Materials and methods** Presently 156 Just stems have been implanted, with a 2-year follow-up. The clinical and radiological results as well as the DEXA studies that have been carried out on the first 100 cases are very encouraging. Stem/bone integration has been demonstrated both clinically and via bone density meter. In no cases has the stem shown subsidence. There has been one revision with a straight, primary stem due to trauma 2 months from the implantation.
**Results** Less blood loss due to non opening of the canal, complete bone preservation of the neck with the exclusive substitution of the femoral head which guarantees optimal functional recuperation and physiological offset reproduction of the hip, independent of the cervical-diaphyseal angle.
**Discussion** The femoral neck is totally preserved, which implicates the maximum preservation of bone stock and for this reason this type of stem represents the only real alternative to the hip resurfacing prosthesis. Femoral head necrosis does not represent a contraindication to this prosthesis implant. On the contrary, the implant of the acetabular cup results more damaging and in fact use of mini invasive acetabular reamers.
**Conclusions** The new total neck preservation prostheses represent a true alternative to the current prostheses, which are to date less utilized. For young patients these surely represent the best choice for elevated bone stock preservation.The Just prosthesis inserts into the femoral trabecular bone system obtaining optimum primary stability and secondary osteointegration reproducing the joint physiological offset independent from the cervical-diaphyseal angle.

### Gait analysis of THA with different head diameters: a prospective randomized study

L. Zagra[1], L. Bianchi*[1], V. Licari[2], C. Champlon[3], R. Giacometti Ceroni[1]

[1]Chirurgia dell'Anca 1, Istituto Ortopedico Galeazzi IRCCS (Milan, IT);
[2]Medicina Riabilitativa 1, Istituto Ortopedico Galeazzi IRCCS (Milan, IT);
[3]Laboratorio di Gait Analysis, Istituto Ortopedico Galeazzi IRCCS (Milan, IT)

**Introduction** In recent years thanks to the development of biomaterials with lower wear, the use of prosthesis with bigger diameter head has increased. Bigger heads have advantages such has reduced risk of dislocation, an increased range of motion and perhaps a feeling of more "physiological" hip. The aim of this study is to evaluate the walking recovery of patients after THA with different head diameters by the means of gait analysis.
**Materials and methods** A prospective randomized blind study was conducted on 60 patients operated by the same THA with different head diameter. Inclusion criteria were: primary hip arthritis, women, age between 55 and 70 years. Exclusion criteria were: other problems influencing walking ability. The same uncemented stem, same uncemented press-fit cup, same surgical technique and approach (posterolateral), same surgeons, same postoperative protocol and rehabilitation were employed. The only difference was the head diameter. The patients were randomized into three groups, of twenty patients each one (> 42 mm Met-on-Met, 36 mm Cer-on-XPE, 28 mm Cer-on-XPE). The gait evaluation was performed on three temporal steps: pre-operatively, two months post-operatively and four months post-operatively.
**Results** The first group (head > 42 mm), 2-month after surgery was better than other groups in 3 variables (stance percentage, double support percentage and stride length) and 4-month after surgery was better in 2 variables (stride length and step length). In the second group (36 mm head), 3 variables were better than other groups at 2 months: (cadence, stride width, average speed). At 4 months the improvements were less than the other two groups. The third group (28 mm head) was better than the others in step lenght and swing speed at 2 months after surgery and has an index of improvement greater than the other groups at 4 months in 6 parameters. So the first two groups had an index of improvement better in 3 variables at 2 months after surgery, while the last group at 4 months after surgery has a higher rate of improvement in 6 variables of 8 total variables.
**Discussion** There were no statistically significant differences for the kinematic variables between the three groups, but all the variables assessed at 4 months after surgery improved significantly in all groups.
**Conclusions** This study shows that there are no statistical significant difference in standard gait analysis parameters in patients with different head diameters (> 42 mm, 36 mm, 28 mm) after THA.

### The treatment of lateral hip fractures by intramedullary nail "Veronail": our experience

G. Caputo*, G. Fanzone, A. Rossitto

Ospedale "M. Chiello" ASP 4 (Piazza Armerina-Enna, IT)

**Introduction** Each year, the treatment of lateral hip fractures, need high economic resources. We believe that the treatment of choice should be characterized by mininvasive surgical technique, which allows a reduction of blood loss and reduction days of hospitalization, as well as the use of fixation devices good value for money.
**Materials and methods** We treated from 2008 to 2011, 143 patients with "VeroNail" (mean age, 77 years). All patients were studied by X-ray examination and all hip fractures were classificated of according to AO principles. Of the 143 nails VeroNail implanted, only 4 were long nails, in the remaining cases were implanted VeroNail standard. We used the cephalic parallel sliding screws in 123 patients and the cephalic converging screws in 16 patients. Was performed in all patients the distal locking nail: we preferred the distal dynamic configuration of the system to not increase the rigidity of the system and to limit the cephalic screws cut-out. Post-operative rehabilitation was always performed to functional recovery of the limb.
**Results** In all cases, the clinical and radiographic healing of the fracture was achieved within 3 months after surgery. The average of hospitalization days was five. We granted immediately partial load in patients with stable fractures. Walking with partial load was granted for the unstable fractures only after three weeks. The functional recovery of fractured limb was comparable to that existing before the fracture.
**Discussion** Our approach was to use systems that have a second anti-rotational screw, to ensure more stability at the synthesis. The marketing of a nail with double-screw cephalic in parallel or converging configuration, has made the system VeroNail our reference in the treatment of extracapsular proximal fractures femur. However we have not always limited to implant only a single brand nail, but we are continuing to check the validity of the twin cephalic screws systems compared to single cephalic screw.
**Conclusions** Our experience with "VeroNail", confirms the validity of this nail for the treatment of the pertrochanteric hip fractures as well as basicervical hip fractures. This nail is suitable to the treatment of persottotrochanteric femur fractures by convergence configuration of the cefalic screws too. Less suitable for young people in the impossibility of changing and the difficulty encountered with the removal of the nail. The use of this nail is not indicated in young patients because it is impossible to change the neck-shaft angle and because it is very difficul to remove.

### Treatment of avascular necrosis of the femoral head: the role of hip arthroscopy

N. Santori*[1], A. Bertino[1], V. Arceri[2], E. Theodorakis[2], D. Potestio[3]

[1]Casa di Cura Quisisana (Rome, IT);
[2]Policlinico Umberto I (Rome, IT);
[3]Casa di Cura Villa del Rosario (Rome, IT)

**Introduction** Avascular necrosis of the femoral head (AVNFH) mostly presents in young patients between the ages of twenty and forty years. The disease affects the vascularisation of the femoral head and not directly the articular surface. If untreated, AVNFH typically progresses to subchondral and articular collapse and early osteoarthritis. Conventional staging systems, based on lesion size/location and radiographic appearance, do not provide information on the real conditions of the joint surface. Above all, imaging, often does not detect detachment of the necrotic fragment with interruption of the articular surface.
**Materials and methods** In the last ten years, with the ambition of maximising the chances of success of non prosthetic surgical treatment in early stages AVNFH patients, we introduced arthroscopic joint evaluation for selected cases. Criteria for arthroscopy were atypical symptoms in contrast with the radiographic appearance and young age. Between 2000 and March 2010, we performed 34 arthroscopies in AVNFH.
**Results** In 9 hips out of 29, arthroscopy showed complete detachment of the necrotic fragment of the head. These cases were rescheduled for total hip replacement. In 25 hips, even with partial collapse of the subchondral bone, there was continuity of the femoral head articular surface. These cases, after arthroscopic assessment, were directly treated with core decompression and autologus bone grafting.
**Discussion** Since both procedures are carried out on the traction table, the addition of hip arthroscopy took, on average, only 20 extra minutes. Rate of success of non prosthetic surgical procedures for early and intermediate AVNFH stages depends of a number of factors. Complete detachment of the necrotic fragment is a negative prognostic factor for non prosthetic treatment. In our experience, hip arthroscopy is a useful additional tool for accurate staging and for selection of the best possible treatment. Larger numbers are needed to draw definitive conclusions.

**Conclusions** In our experience, hip arthroscopy is a useful additional tool for accurate staging and for selection of the best possible treatment. Larger numbers are needed to draw definitive conclusions.

### Gait recovery after total hip replacement: one-year longitudinal assessment of primary and compensatory gait deviations

M. Fravisini*[1], M. Longhi[2], D. Mazzoli[2], A. Baccarani[1], A. Merlo[2], P. Prati[2]

[1]U. O. Ortopedia, O.P.A. Sol Et Salus, Torre Pedrera (Rimini, IT); [2]Laboratorio di Analisi del Movimento e di Biomeccanica, O.PA. Sol et Salus, Torre Pedrera (Rimini, IT)

**Introduction** The decision of hip replacement tends to be delayed as much as possible, due to the limited time duration of the prosthesis and to minimize risk of a second replacement. The aim of this work was to perform a one-year follow-up of kinematic and kinetic gait deviations of patients that underwent hip replacement and to investigate if clinical and functional conditions just prior to surgery influenced the recovery over time.

**Materials and methods** Patient gait was acquired before surgery, 2 weeks, 6 months and one year after hip replacement. 26 patients were enrolled, 21 of them completed the study.

**Results** Walking speed and ten gait related variables were investigated and their variation over time was assessed by means of the Wilcoxon test. The effects of initial hip force, retractions and dynamic angular ranges on final gait speed, hip sagittal range and hip extension symmetry were assessed by the U Mann–Whitney test and the Spearman correlation coefficient respectively.

**Conclusions** Gait speed, hip dynamic flexion–extension range and pelvic tilt range progressively improved during the follow-up year, while most of the compensatory gait deviations diminished during the year. Initial conditions minimally differentiated the degree of recovery in the sample patients after one year, who had all reached both a nearly normal hip flexion–extension range and gait speed. Due to the sample characteristics, these results pertain to patients with mild or no force reduction of hip muscles before surgery.

### *C52 – HIP 8*

### Early experiences with Vitae stem

P. Parchi*, N. Piolanti, C. Giannetti, P. Battistini, M. Lisanti

1a Clinica Ortopedica, Università di Pisa (Pisa, IT)

**Introduction** Titanium has been used in clinical practice for 35 years and it has proven to be extremely biocompatible. The further development of porous titanium has made it an excellent material for prosthesis implant. The aim of this paper is to present the first experiences with Vitae Adler Ortho Stem neck retaining modular stem built with Ti-Por® titanium.

**Materials and methods** We will report the preliminary experiences with Vitae Stem in the 1st Orthopaedic Department of Pisa. The preoperative evaluation included X-rays of the hip, clinical evaluation, Harris Hip Score and Western Ontario and McMaster Universities Arthritis Index. The surgical approach was postero-lateral in all patients. We collected clinical and radiological data.

**Results** The intraoperative results using this stem showed an excellent primary stability due to triplanar stability of such kind of stem and to titanium Ti-Por® roughness. The modularity (27 different neck combinations) allowed an easier implant technique with a restoration of original articular biomechanics. Also the post-operative clinical recovery and short term radiological results were satisfactory.

**Discussion** Short stems represent a less invasive choice and an interesting alternative for hip prosthesis. The design and biomechanics of these implants allow the preserving of diaphyseal bone stock and a more physiological distribution of loading forces. Long-term results begin to emerge from literature and are encouraging. Porous materials allow implant integration and long term survival.

**Conclusions** Our experience with Vitae Stem is short, but the clinical and radiological results are satisfactory. The design of this stem is indeed updated, implementing the philosophy of metaphyseal loading, modular neck and porous coated implant. Technical, bibliographic and scientific assumptions together with our preliminary results, allow supposing that this stem will have good results in the long run.

### The new stemless hip implants: our experiences with the ultra short stem

G. Logroscino*[1], G. Magliocchetti Lombi[2], V. Ciriello[1], D.A. Santagada[1], C. Fabbriciani[1]

[1]Istituto di Clinica Ortopedica, Università Cattolica del Sacro Cuore (Rome, IT); [2]Casa di Cura San Feliciano (Rome, IT)

**Introduction** “Ultra-short hip stems” in prosthetic surgery are increasingly more widely used and allow preservation of “bone stock”. Even if these new implants are very promising in particular in young patients, the reliability is scarcely described in Literature. The aim of this study is to assess the effectiveness of a new “stemless” hip implant.

**Materials and methods** A new cementless neck preserving, stemless hip prostheses (NANOS; Smith & Nephew) was implanted between 2005 and 2010 in 136 patients with a mean age of 55.5 (M:F ratio 2.3:1) affected by osteoarthritis or avascular necrosis. All the patients were followed radiographically (Engh’s criteria) and clinically (HHS, VAS pain and SF-12) before surgery and at 1, 3, 6 and 12 months postoperatively with a mean follow-up of 30 months (min. 6 and max. 66).

**Results** The rate of implant failure (MFR) was 0%, with a percentage of osseointegrated implants of 100% and consistent improvement in recover from pain, in joint function and in the quality of life (HHS = 98, SF-12 = 96 VAS < 3 mm.). Some complications were observed: 1 metaphyseal crack, 2 perforations of the diaphyseal cortical bone, 2 dislocations, 4 leg length discrepancies (> 1 cm., but < 2 cm.) and one cup aseptic loosening.

**Discussion** Ultra short neck preserving stems showed to allow min-invasive surgery associated to primary stability and excellent preservation of the proximal femoral bone quality. Some limitations were observed in the correction of leg length. Nevertheless the clinical results were certainly better, at early follow-up., than of the traditional stems.

**Conclusions** The new stemless implant showed to be well indicated in young patients or in case of well preserved proximal bone quality.

### MiniMAX stem: preliminary results of a new anatomic stem implanted using the anterior approach

P.G. Vasina*, R. Rossi, P. Palumbi, G.M. Giudice, M. Barchetti, M. Di Scioscio, F. Mazzotti, P. Saccozzi

AUSL Ferrara (Argenta, IT)

**Introduction** We analyse clinical and radiological results of 146 patients who were operated on cementless hip artroposthesis with MiniMAX® anatomic stem (Medacta International SA), between October 2007 and December 2010, through an anterior approach.

**Materials and methods** The prosthesis was chosen because it is designed following the modern concepts of shape and anchorage, which must be performed between femur and metallic implant to optimize "fit and fill". It is an anatomic stem prosthesis. The contemporary acetabular shell implant was done using the cup Versafitcup® CC (Medacta International SA), as press-fit, with a ceramic insert. All surgeries were performed by using AMIS® technique (Anterior Minimally Invasive Surgery, Medacta International SA). The anterior approach is the only technique that follows a path both intermuscular and internervous and therefore considerably reduces the risk of damaged periarticular structures. To facilitate the surgical phases and to make the surgery reproducible, we add an integrative extensor device to the standard operating table: the AMIS® Mobile Leg Positioner which allows hip flexion, extension, abduction, adduction and internal and external rotation. The use of this device allows the reduction of: (1) theatre assistants required throughout the procedure; (2) surgical phases reproducibility.

**Results** We found the following relevant advantages: no muscles cut, significantly shorter rehabilitation, a small skin scar, decreased postoperative pain, less blood loss, reduced hospital stay, no dislocation, faster return to daily activities. In the short-term, the stem is radiographically well integrated in the bone with a good metaphyseal grip. No loosening has been highlighted. Two revisions were performed because of suspected infection.

**Discussion** The curved design of the stem on both surfaces, sagittal and frontal, facilitates the insertion through a small incision. Also dimensions, which are shorter respect to other prosthetic patterns, are suitable for a mininvasive approach. The clinical result of lack of thigh pain in all examined cases confirms the choice of a specific and unique tip design optimised to guide the prosthesis insertion into the femoral canal and to avoid distal interference.

**Conclusions** Very good efforts in the short-term support a good synergy in choosing the implant of this anatomic stem with anterior approach.

### Evolution in osseointegration of standard stem versus neck preserving stem in total hip arthroplasty

E. Gasbarra*, R. Lundusi, M. Celi, I. Cerocchi, F.L. Perrone, U. Tarantino

Fondazione PTV, UOC Ortopedia e Traumatologia, Università degli Studi di Roma Tor Vergata (Rome, IT)

**Introduction** In the last years, neck preserving femoral stems were more and more frequently used, due to the minimally invasive surgical technique and to less metaphyseal resorption with respect to standard stems. Aim of the study was to compare bone remodelling, regain of ROM and ambulation, and blood loss in patients treated with neck preserving stems or standard stems.

**Materials and methods** We studied 33 neck preserving implants (Fitmore and Trialoc) and 35 standard stems (TMP). Average age of patients was 62 years (range, 24–83) in the first group, and 72.3 in the second group (range, 26–98). Both groups had similar BMI. Functional regain was evaluated by HHS and Timed Up and Go (TUG) test. Bone remodelling was measured by DXA immediately after intervention, and at 3, 6 and 12 months based on Gruen protocol. Coagulation indexes, peri-operative blood loss and quality of life by SF-36 were also evaluated.

**Results** Results analysis evidenced faster functional regain, measured by HHS and TUG, in patients with neck preserving stems. Densitometry showed higher BMD at ROI 1 after 12 months for standard stems (+6,7%) and an increase of BMD by +4,1% and +8,3% at ROIs 3 and 7 for neck preserving stems, while, at the same ROIs, standard stems showed values of −0,5% and −2,3% respectively. In patients with conservative stems Hb loss was 2.1 mg/dl lower with respect to standard stems. Obtained data showed no significant age-related changes.

**Conclusions** Neck preserving stems showed good short and long time results. DXA study of peri-prosthetic bone remodelling demonstrates lower metaphyseal resorption compared to standard stems, due to reduced size at the proximal diaphysis and better load distribution in the metaphyseal areas. Functional regain was also faster, thanks to less invasive surgery and contained blood loss. They can therefore represent a good alternative to standard stems, which require more invasive techniques and higher loss of bone-stock immediately after surgery, both in younger and elderly patients.

### *C53 – BIOMATERIALS AND COATINGS IN ORTHOPAEDICS 5*

### Short stem and porous titanium: is it a winning combination?

M. Lisanti*[1], C. Fioruzzi[2], U. Fusco[3], M. Olivieri[4]

[1]1ª Clinica Ortopedica, Università di Pisa (Pisa, IT);
[2]ASL Piacenza (Piacenza, IT);
[3]Ospedale Galmarini Tradate, USL Busto Arsizio (Tradate-Varese, IT);
[4]Ospedale Galliera, ASL 3 Genova (Genoa, IT)

**Introduction** Short stems have had in the last years a remarkable development, but some surgeons are still not sure about their rotational and vertical stability and this limits their diffusion. In our opinion, porous titanium has fully changed prospects by using short stems.

**Materials and methods** The primary stability of a short stem is linked both to the design and the surface. The topic of this paper is Stem Vitae Adler Ortho. This stem has a design that allows high primary stability and a titanium coating with a high initial grip due to porosity with interconnected titanium pores and additional 40 micron spikes at the junction of the pores (Ti-por®). Vitae Stem was designed with the philosophy of minimal diaphyseal invasion, metaphyseal load, cancellous bone saving and adaptability to the major part of the proximal metaphysis.

**Results** The first 100 implants gave satisfactory clinical and radiological results even though within a short follow-up (max. 10 months).
**Discussion** As demonstrated in the literature dimension and geometry of porous, materials have a fundamental rule in promoting bone in-growth and biological stabilization of the implants to bone. The production technique of Electron Bean Melting [EBM] Adler-Ortho makes it possible to obtain optimum porosity at the peripheral level, which is ideal for the trabecular graft and consequently to ensure secondary osteointegration. The developed technology with Ti-por® titanium allows to cover Vitae Stem with an extremely rugged (3D pores 700 micron) envelope. With modularity, (27 different neck combinations) this stem allows a perfect restoration of the hip geometry.
**Conclusions** Currently there are no short stems with this coating, which according to previous studies allow bone in-growth and consequently ensure secondary stem osteointegration. Its small size allows safety and rapidity of use, less bleeding and absence of thigh pain.

### Fixa Adler Ti-Por cup with coating surface in porous titanium: clinical and radiographic follow-up to 2 years

G. Sacchetti*, S. Bondioli, C. Tsatsis, F. Pinto, A Ambrosio, F. Catani

Modena, IT

**Introduction** Fixa Ti-Por Adler acetabular cup is a press-fit hemispherical 4th generation cup that improves the primary press-fit and promotes secondary bone-integration. Electron Bean Melting process allows coating bone contact surface with three-dimensional microstructure of titanium. This process promotes bone tissue growth in titanium's interstices, to obtain optimal size between 300 and 1000 micron, exactly 700 micron in cone penetration. Bone growth's surface was generated from a CAD model to obtain a three-dimensional surface with outer tips to improve primary fixation to bone. To obtain this surface you must use dust technology. This surface is forged with the cup from which it can't be removed or released, adding strength and endurance to the system. You can coup with polyethylene liners, metal and Delta ceramic, with possibility of using large diameter heads. The aim of our study was to evaluate the outcomes of patients treated with this technique, with a 2-year follow-up.
**Materials and methods** From April 2009 to February 2011, we implanted 129 Fixa Ti-Por acetabular cups in primary total hip arthroplasty with uncemented anatomic Apta stem and neck preservation Parva stem. We treated patients with acetabular dysplasia, coxa profunda and also elderly patients with osteoporosis, without intra-operative and post-operative complications. Clinical evaluations were performed with HHS, WOMAC and HOOS; radiographic evaluations with X-rays in A/P and L/L cross-table view, with measuring cup inclination and anteversion and in some cases with Dexa study to evaluate the mineral distribution around the cup.
**Results** In the first cases we had some position defects because reaming did not match to cup size and we were not able to reach the shedding of cup completely, without compromising cup's primary stability. Nowaday no cup was revised. We never needed to use stabilization acetabular screws. We coupled ceramic/ceramic in 90% of cases, never metal to metal.
**Conclusions** In our experience the use of 4th generation-porous titanium coated cup with dust technology allowed the press-fit implant without use of acetabular screws and with excellent primary stability and rapid secondary bone-integration. We believe this type of cup to have extended indications for hip joint pathology, because of also coupling to be performed.

### Innovative hydroxyapatite nanostructured coatings on titanium substrates by pulsed laser deposition (PLD)

A. Bianco*[1], I. Cacciotti[1], J.V. Rau[2], S. Laureti[3], M. Fosca[2], V. Rossi Albertini[2], A. Ravaglioli[4], M. Celi[5], I. Cerocchi[5], U. Tarantino[5]

[1]Dipartimento di Scienze e Tecnologie Chimiche, INSTM UDR Roma "Tor Vergata", Università di Roma "Tor Vergata" (Rome, IT);
[2]Istituto di Struttura della Materia, Consiglio Nazionale delle Ricerche (Rome, IT);
[3]Istituto di Struttura della Materia, Consiglio Nazionale delle Ricerche (Monterotondo Scalo-Rome, IT);
[4]Parco Torricelli delle Arti e delle Scienze (Faenza-Ravenna, IT);
[5]U.O.C. Ortopedia e Traumatologia, Fondazione PTV Policlinico Tor Vergata, Università degli Studi di Roma "Tor Vergata" (Rome, IT)

**Introduction** Synthetic hydroxyapatite (HAp, $Ca_{10}(PO4)_6(OH)_2$) is a material widely used in osteosynthesis devices, due to its similarity with the main inorganic component of bone. Bioactivity of HAp can be improved by the incorporation of different cations (i.e. $Sr^{2+}$, $K^+$, $Mg^{2+}$) and/or anions (i.e. $F^-$, $Cl^-$, $CO_3{}^{2-}$, $SiO_4{}^{4-}$), in order to mimic the composition of biological apatites. Several in vivo animal models studies have demonstrated that the incorporation of silicon within the apatite lattice promotes the osteoblastic activity, allowing enhanced biological performances in terms of bone apposition, bone ingrowth and cells mediated degradation [1]. The PLD (Pulsed Laser Deposition) technique presents pivotal advantages, such as good reproducibility of target composition, control of coating adhesion, crystallinity, and roughness associated to limited contamination. Si-substituted hydroxyapatite coatings could further increase the stability and the osteointegration of bone implants in orthopedical surgery.
**Materials and methods** Si-substituted (Si content 1.4%wt) hydroxyapatite films were deposited on the treated Ti substrates at different substrate temperatures (namely: 400, 500, 600 and 750°C) in a high vacuum PLD chamber, using as target a disc obtained by sintering wet precipitated nanopowders [2]. Depositions were performed by focusing a pulsed KrF Lambda Physik excimer laser on the rotating target. The microstructure was studied by microscopy techniques (scanning electron, SEM and atomic force, AFM), infrared spectroscopy (FT-IR) and X-Ray diffraction (XRD). Microhardness measurements were also carried out. Besides, preliminary bioactivity in vitro tests were performed.
**Results** The optimal parameters (i.e. laser fluente and substrate temperature) for the deposition of Si-HAp coatings were identified (i.e. 2 $J/cm^2$ and 600°C), aimed to obtain uniform, homogeneous films with the composition close to the target one. The produced coatings resulted dense, compact and characterised by low surface roughness and a thickness of 1.5 μm.
**Discussion** The experimental investigations demonstrated that the employed deposition technique allowed obtaining homogeneous nanostructured thin films of composition very similar to the target one. In vitro tests evidenced the formation of an apatite layer, suggesting an improved bioactive behaviour. Microhardness measurements corroborate the potential application perspectives of these coatings for osteosynthesis implants.
**Conclusions** In this work the PLD technique suitability to the deposition of Si-substituted hydroxyapatite coatings was demonstrated. The produced bioactive nanostructured coatings satisfied the requirements necessary for the applications in osteosynthesis devices.

**Suggested readings**
1. Pietak AM, Reid JW, Stott MJ, Sayer M (2007) Biomaterials 28:4023–4032
2. Bianco A, Cacciotti I, Lombardi M, Montanaro L (2009) Mater Res Bull 44:345–354

### Resorbable Herbert screws in capitellum fractures fixation

R. Rotini*, E. Guerra, A. Marinelli, M. Cavaciocchi

Istituto Ortopedico Rizzoli (Bologna, IT)

**Introduction** The use of resorbable materials is one of the main recent applications of bioengineering in orthopaedics. We report our experience in osteosynthesis of capitellum fractures by resorbable Herbert screws.
**Materials and methods** In the period 2005–2010, at the Shoulder and Elbow Surgery Unit of the Rizzoli Institute, 10 cases of capitellum fracture have been treated. There were 3 men and 7 women, mean age 46.4 yearrs (range, 15–65 yrs). According to McKee, fractures have been divided in: type 1, 3 cases; type 2, 1 case; type 4, (limited to capitellum) 1 case; type 4 (shear fractures), 5 cases. Type 3 fractures (highly comminuted) were excluded. ORIF was performed by Herbert resorbable screws and pins (poly-L-lactic acid, RSB Implant, Hit Medica, San Marino). Follow-up was 31.7 months (range 12–65). An MRI evaluation was performed to check the process of resorption of the biomaterial and any articular reaction at least at one-year after surgery. In every case a stable intra-operative fixation was achieved, allowing to begin the rehabilitation the day after surgery (early CPM). In two cases a screw broke during positioning, because of a non perfect congruency between the tapped hole and insertion axis.
**Results** The fractures healed in all cases with a mean MEPS score of 98 (85–ma100). No chondrolysis or foreign body reactions have been observed at follow-up.
**Discussion** The main advantage in the use of PMMA is to achieve an anatomical fixation passing through the articular cartilage to reach subchondral bone; the in–out technique is better in order to fix the small fractured fragments with a compression force; further surgery to remove the hardware has not to be expected.
**Conclusions** We tested the safety and the effectiveness of capitellum fractures osteosynthesis by resorbable screws. On the other hand the surgical technique has to be extremely precise because of the fragility of the resorbable implants.

### A new porous/osteoconductive cement: experimental study

C. Dall'Oca*, T. Maluta, G.M. Micheloni, P. Bartolozzi

Clinica Ortopedica e Traumatologica, Policlinico G.B. Rossi (Verona, IT)

**Introduction** A new bone acrylic cement (Porosectan) made of PMMA and ß-tricalciumphosphate is tested for evaluate its biocompatibility, osteoconduction and osseointegration.
**Materials and methods** We implanted cylindric samples (2 × 6 mm) in 12 New Zealand White rabbits. In particular samples of Porosectan (powder + granules) in right femur proximally, Porosectan (powder) in right femur distally and PMMA in left femur as a control. Couples of rabbits were sacrificed after 1, 2, 3, 6, 9 e 12 months.
**Results** Porosectan, with low polymerization temperature, is turned out a biocompatible cement. The increase of cement-bone interface, due to its porosity and the formation of new-borne trabeculae, not only at the interface, prove the optimal osseointegration of this cement. Porosectan shows its porosity, with 200–500 μm pore size, which create a structure open to outside. Moreover this cement is penetrated by organic fluids and cells superficially, that allow us to considerate Porosectan as a osteoconductive cement, in particular the formulation powder + granules seemed to be the right structure for the formation of a scaffold that allows bone cell neoformation inside.
**Conclusions** Neotrabeculae growth inside the cement is histologically demonstrated, and after 12 month involve all Porosectan (powder + granules) structure. This, could be related to $\beta$-TCP reabsorption that releases osteoinductive factors like calcium and phosphate ions.

### Ceramic-on-ceramic bipolar head in partial hip replacement: a mid-term experience

A. Olmeda*, S. Zanarella, P. Friso

Azienda Ospedaliera di Padova (Padua, IT)

**Introduction** Femoral neck fractures represent currently one of the thoughest problems for our National Health Service. There are not comproved differences in outcome between partial arthroplasty with bipolar head and total hip arthroplasty, especially in elderly patients. The main cause of bipolar head failure is due to polyethylene wear, that leads to a grip of the inner head with the external cup. Ceramic-on-ceramic bipolar head can avoid this complication, and allow extending the life of the prosthesis quite as long as a THA, with a less invasive and a cheaper surgery.
**Materials and methods** From 2005 to 2010, we performed partial hip replacement with ceramic-on-ceramic bipolar head in 375 patients. For the implants we employed only uncemented press-fit stems. The materials were CeramTec Duo bipolar head and SL Plus or Proxy Plus (Smith & Nephew) stems. The mean age at the time of surgery was 81.5 (range 51 to 98), the timing was 36 h. Full load on the side of prosthesis was restored after three days from surgery. All of the patients were controlled with clinical and radiographical examination six weeks after surgery. The minimum follow-up was 6 months (mean 17.5%), 85% were controlled with clinical examination or phone interview.
**Results** We saw 2 periprosthetic fractures, 1 infection, 2 dislocations (all treated for intertrochanteric fracture), 2 periprosthetic fibrosis (asymptomatic). We saw non cases of intolerance or ceramic breakage. At the follow-up, the sinking of the stem was below 1.5 mm. Pain was absent or mild in 84.9% of patients. Six months after surgery, 82% of patients returned to normal life and were able to walk individually, or using one cane or crouch.
**Discussion** With the new ceramic-on-ceramic bipolar head we did not noticed new or unusual complications, the intra.-operative assembling is easy and does not extend the time of surgery. The follow-up was free from accidents, and the new bipolar head seems reliable. Considering problematic outcome of THA in some elderly patients, and resurfacing in younger ones, bipolar ceramic-on-ceramic cup may become an ultimate solution for partial hip arthroplasty in elderly patients and a good solution for the other categories.
**Conclusions** On a mid-term follow-up we can demonstrate that no particular complications have been observed. In our experience, outcome of bipolar ceramic-on-ceramic cups has no difference in comparison with the other kind of implants.

### *C54 – BIOMATERIALS AND COATINGS IN ORTHOPAEDICS 6*

### Preliminary outcomes of bone marrow concentrate and AMIC technique in 24 knee chondral lesions in patients aged between 55 and 65 years

A. Siclari*, T. Aramo, M. Piras, M. Vigna Suria, E. Boux

(Biella, IT)

**Introduction** One of the most important factor limiting the treatment of chondral injuries is the age of the patient. In the most recent technique, the AIMC, chondral regeneration is demanded to mesenchimal cells (MSC) of sub-chondral bone. As well known, the number of MSCs in sub-chondral bone depends on many factors, between those the most known is the age of the patient. Experimentally MSCs in patients aged 55 to 65 appear as efficient as these of younger patients to become condrocities, but fewer.
**Materials and methods** To get around this problem, from June 2009 to February 2010 we treated 24 patients from 55 to 64-year-old presenting chondral knee injuries with AMIC technique plus PRP, as we did before in younger patients, but adding bone marrow concentrate to increase cell number. 15 and 30 days after surgery patients received a PRP knee injection.
**Results** We excluded patients with load alteration or previous surgical treatments. Patients were re-evaluated with KOOS after 1, 3, 6, 12 months of follow-up: average score increased from 53.82 (before surgery) to 81.85.
**Discussion** 23 cases had an MRI examination 1 year after surgery, demonstrating a good reconstruction of cartilage surface. In 22 cases we carried out knee fibroarthroscopy after 4 and 8 months, demonstrating good cartilage regrowth, similar to younger patients.
**Conclusions** No biopsy was performed. No patient needed successive surgical treatment. Clinical outcomes at short-term follow-up are now very encouraging, long-term results will give us more information to give definitive judgement.

### Why adding vitamin E to UHMWPE?

L. Costa*[1], P. Bracco[1], P. Dalla Pria[2], D. Masoni[2]

[1]Dipartimento di Chimica IFM (Turin, IT);
[2]Samo (Bologna, IT)

**Introduction** Although UHMWPE has been successfully used in total joint replacement for more than 30 years now, its lifetime is often limited due to oxidative degradation induced by radiation with high energy radiation in air. Alkyl macroradicals formed upon irradiation can react with oxygen dissolved in the polymer matrix, triggering an oxidative cascade that reduces UHMWPE molecular mass with consequent worsening of mechanical properties, decrease of the abrasive wear resistance and increase of wear debris. The incorporation of the antioxidant vitamin E has been proposed recently as an alternative to postirradiation melting to avoid oxidation and simultaneously preserve the mechanical properties of crosslinked UHMWPE.
**Materials and methods** GUR 1020 UHMWPE plates, blended with vitamin E (0.1%wt) were irradiated at 25 to 100 kGy and tested without any further thermal treatment. The materials were characterized by FTIR, DSC, crosslink density and mechanical properties before and after accelerated aging for 14 days.
**Results** Cross-link density increases with radiation dose. The maximum oxidation index measured before ageing was 0.05 on all samples. After ageing, the maximum OI was measured in the sample irradiated to the highest dose, but even there it did not exceed the value of 0.14. Percent crystallinity of the irradiated blends increases slightly with the radiation dose and does not vary significantly after ageing, confirming that no significant structural changes occur to the polymer. The mechanical properties of irradiated vitamin E blended UHMWPE do not differ from that of unaged, irradiated virgin UHMWPE. While the mechanical performances of irradiated UHMWPE are well known to deteriorate rapidly, the tensile tests performed on the irradiated blends before and after accelerated ageing show little differences: US decreases slightly, while YS increases and EB decreases.
**Discussion** Vitamin E is known to be a very active antioxidant for polyolefines. We have shown that UHMWPE blended with 0.1%wt of vitamin E exhibit a high oxidation resistance, even after irradiation to 100 kGy in air without any following thermal treatment and subsequent accelerated ageing in a highly oxidizing environment. The efficiency of cross-linking is decreased in the presence of vitamin E, but an additive concentration of 0.1%wt still allows modulating the cross-link density to the desired level.
**Suggested reading**
1. Bracco P et al. (2011) Clin Orthop Relat Res (*in press*)

### The features of chemical, physical and biological properties of the optimal coating material in the AMIC technique

A. Siclari*, T. Aramo, M. Piras, M. Vigna Suria, E. Boux

(Biella, IT)

**Introduction** The treatment of cartilage injuries is a standing problem for the orthopaedic surgeon. Reparing potential of mesenchymal pluripotent cells opened new horizons. Biologically, tissue regeneration requires several factors: cells with the ability to reproduce and differentiate, growth factors, a suitable micro-environment and a scaffold for correct tissue reconstruction.
**Materials and methods** AMIC technique (Autologous Matrix-Induced Chondrogenesis), which we adopted in more than 200 cases, is the most recent way to treat cartilage injuries and runs in a single surgical step, combining micro-fractures that expose therefore mesenchymal ancestors, and a three-dimensional matrix.
**Results** The purpose of this report is to describe the features of chemical, physical and biological properties of the optimal coating material in the AMIC technique. A successful scaffold should give mechanical support, must be able to be biologically active and to allow cell colonization. This balance is often a compromise between a very dense scaffold with excellent resistance and a very porous one which allows an optimal distribution of cells. About this type of bioengineering we can paraphrase the architect Robert Le Ricolais who said: "the art of structure is where to put the holes!"
**Discussion** A coating material must have optimal mechanical, chemical and biological properties to ensure the best result. The mechanical properties: (1) breaking resistance; (2) handling; (3) three-dimensional; (4) visco-elasticity. Chemical properties: (1)

hydrophilic; (2) optimal duration. Biological properties: (1) condro-conduction; (2) biocompatibility; (3) safety; (4) condro-induction.
**Conclusions** The report will discuss all these aspects evaluating scaffolds available and reporting clinical and experimental literature to better define what is the "ideal" scaffold. This work have also the goal of to stimulate research to get as close as possible to a scaffold that satisfies all the requirements for the treatment of cartilage defects.

### Morphological and gene expression analysis of matrix-induced autologous chondrocyte implantation (MACI)

M. Ronga*[1], M. Protasoni[2], M. Reguzzoni[2], A. Passi[3], P. Bulgheroni[1], P. Cherubino[1]

[1]Dipartimento di Scienze Chirurgiche Ricostruttive e Tecnologie Avanzate, Università dell'Insubria (Varese, IT);
[2]Laboratorio di Morfologia Umana, Università dell'Insubria (Varese, IT);
[3]DSBSC (Varese, IT)

**Introduction** Matrix-induced autologous chondrocyte implantation (MACI) is a tissue engineering technique for the treatment of deep articular cartilage lesions. Cultured chondrocytes seeded on a type I/II collagen membrane are implanted in the chondral lesion using fibrin glue. Several authors have evaluated biopsies of the regenerates by light microscopy, while only few results have been obtained from ultrastructural and gene expression analysis after MACI. The aim of this study is to study both structural and molecular evolution of MACI.
**Materials and methods** Three biopsies harvested from 3 different patients at 2-year follow-up were evaluated. In order to define cell phenotype, gene expression and biochemical methods were employed. Biochemical evaluation was performed with Fluorophore Assisted Carbohydrate Electrophoresis (FACE), which studies the composition of the extracellular matrix disaccharide GAG units. On the other hand, type I and II collagen gene expression analysis was performed by Real Time PCR (RT–PCR), which observes the activation of a specific gene during the different polymerization phases.
**Results** FACE demonstrated the presence of glycosaminoglycanes with a pattern resembling hyaline cartilage. QRT–PCR recorded type II collagen gene expression in all samples. In only one case, type I collagen gene expression was observed as well. Histological analysis revealed hyaline-like tissue formation (with type II collagen, condroitinsulfate and tidemark formation) in all cases. In one sample concomitant type I collagen expression was identified at the superficial layer. At ultrastructural evaluation, a hyaline-like cartilage collagen fibrillary network reorganization and cell cluster arrangement were observed.
**Discussion** Ultrastructural and gene expression analysis of MACI reveal hyaline-like cartilage tissue reorganization at 2-year follow-up.
**Conclusions** The present study demonstrates that type I/II collagen membrane is an ideal scaffold for chondroblast growth, allowing hyaline cartilage-like tissue formation once implanted on the chondral defect.

### Bone substitutes and growth factors: from basic science to clinical results

G. Logroscino*[1], E. Pola[1], V. Izzo[1], M. Saccomanno[1], G. Malerba[1], A. Tampieri[2], C. Fabbriciani[1]

[1]Università Cattolica del Sacro Cuore (Rome, IT);
[2]Istituto delle Scienze e Tecnologie dei Materiali Ceramici, CNR (Faenza, IT)

**Introduction** The request for bone substitutes has increased significantly and consequently "osteobiology" developed significantly in the last years. Although several studies reported good results after use of bone substitutes, there are no clinical studies with medium or long term follow-up. The purpose of the present study was to evaluate the efficacy of bone substitutes in the clinical practice.
**Materials and methods** Thirty four patients, treated for fractures with bone loss (15), nonunion/delayed union/cavitary defects (17) or acetabular revisions (2) which required the use of bone substitutes have been reviewed retrospectively through a clinical and radiological (X-ray/CT) examination after 16.5 months of follow up (min: 1 – max: 60 months). The following parameters were evaluated: bone healing, bone substitutes reabsorption and bone replacement, outcomes, adverse events. Finally, it has been drawn up a cost-benefit analysis of the different bone substitutes.
**Results** Forty nine bone substitutes/growth factors/bone graft were implanted. Calcium sulphate (2), DBM (16), PRP (8), HA/P–Ca (8), heterologous bone grafts (3), homologous bone grafts (6), autologous bone grafts (6) were used. Results resulted in: 23 bone healing (67.6%)(at an average follow up of 5.9 month), 44.4% bone substitutes reabsorption or bone replacement, 79.3% of good to excellent clinical results (excellent or good range of motion, painless), 20.7% poor results (moderate pain and/or limited range of motion), 29.4% adverse events or complications (pain, infection, osteonecrosis, nonunion).
**Discussion** In the literature, there is no evidence of a golden standard bone substitute. Actually the best one is still autologous or homologous or heterologous bone. The calcium sulphate, is not osteoconductive and has a too rapid reabsorption, not sufficient for bone replacement. Platelet growth factors are effective only in combination. The DBM are effective, but not totally reliable. The tricalcium phosphate represents a good solution, especially in combination with the DBM.
**Conclusions** Bone replacement is still far from an ideal solution. Bone grafts are still the best available choice, but they have significant morbidity. Bone substitutes, especially tricalcium phosphate in combination with DBM are the most viable alternative.

### Osteoinductive capacity of demineralized bone matrix: a clinical study

D. Carniel*, A. Pozzuoli, R. Aldegheri

Clinica Ortopedica, Università di Padova (Padua, IT)

**Introduction** Advances in biotechnology and the requirement of less invasive strategies for bone healing have led to the introduction of biomaterials for the treatment of bone loss. Among the materials available to the orthopaedic surgeon there is the demineralized bone matrix (DBM). The aim of this study was to evaluate the osteoinductive capacity of DBM in the short term.
**Materials and methods** The study is ongoing and so far we used demineralized bone matrix (DBM) in 27 patients (17 males and 10 females; mean age of 44 years); 5 treated for enchondromas or bone cysts, 2 for correction of deformities, 20 for loss of substance in complex fractures or nonunion. The DBM was used in 22 cases alone and in 5 cases, in which the loss of substance was greater than 2 cm, in combination with bone substitutes. Patients underwent clinical and radiographic follow-up at 3, 6 and 12 months and were evaluated clinically for pain and joint function and radiographically for bone healing. The patients with a positive follow-up at 3 months were considered healed.
**Results** All cases of bone cysts, deformities, and enchondromas healed. 70% of patients treated for complex fractures or nonunions achieved healing within 3 months. There were no cases of surgical site infections or osteomyelitis.
**Discussion** The demineralized bone matrix was very useful in filling bone cysts or enchondromas, leading to healing. Its use was also very successful in the treatment of nonunions and bone gaps in comminuted fractures.
**Conclusions** The results obtained till now prove that DBM is very effective and valid, if used in the indications we have proposed, reducing the healing time. Furthermore none of the patients required additional surgery.

### *C55 – KNEE 7*

### Allograft versus autograft in first planting ACL reconstruction

E. Lupetti*, C. Goretti, A. Belluati, A. Colombelli

Ospedale Santa Maria delle Croci (Ravenna, IT)

The report assesses the clinical results obtained from a prospective randomized study on a population of 30 patients treated for ACL reconstruction with hamstrings autograft (15) and cadaveric allograft for anterior or posterior tibial tendon (15). The report highlights the benefits and limitations of such techniques, comparing the clinical results obtained in the short and medium term, evaluation grids and compared with internationally accepted.

### Use of homologous fresh osteochondral grafting in the treatment of knee articular cartilage defects

D.M. Donati*[1], L. Cevolani[1], T. Frisoni[1], F. Catani[2], S. Giannini[1]

[1]Istituto Ortopedico Rizzoli (Bologna, IT);
[2]Clinica Ortopedica e Traumatologica, Università degli Studi di Modena e Reggio Emilia (Modena, IT)

**Introduction** The traumatic lesions of the articular cartilage of the knee are a frequent pathology of athletes. The symptoms are tumefaction, joint block, instability, pain during activities (especially when climbing stairs) and maximal flexion. The aim of this work is to develop a new surgical technique that can allow the patient to start again taking physical activity without limits and to preserve the function of the knee joint.
**Materials and methods** From December 2006 to February 2011, five patients were treated. The average age was 30 years (range 18–46 years). The patients had a lesion at the medial condyle of the femur and were treated with a fresh osteochondral grafting. They were followed for an average of 13 months (range 1–51 months) and underwent clinical examination every 3 months for the first year, and then half-yearly checks.
**Results** The treatment was 100% successful. Three patients had good joint function 6 months after surgery without pain during the physical activity. One patient presented articular stiffness and lameness.
**Discussion** At present, there is not a standard treatment and there are many treatment proposed for this disease. They consist of microperforation, homologous transplantation of chondrocytes or prosthetic joint replacement. However, the prosthetic joint replacement should be considered as a last instance because of the young age of the subjects affected by this disease and the need to return to physical activity without limitation. The treatment that we propose allows patients a rapid recovery of physical activity, it is effective and safe.
**Conclusions** The osteochondral grafting is indicated when facing a full-thickness cartilage lesion with irregularity or loss of bone substance of the basic system of the cartilage. Other factors that may determine the success of the technique are the presence of an isolated cartilage lesion, ligamental stability and the absence of axial defects. Our treatment is effective for the therapy of the osteochondral disease because it allows the rapid recovery of limb function load and a functional recovery of the joint. Moreover, the risks of this technique are minimal.

### Total knee arthroplasty in rare case of type 3 Von Willebrand arthropathy

C. Ruosi*, A. De Caro, C. Cavallaro, F. Granata, D. Rossi, S. Liccardo, D. Di Minno, A. Coppola, E. Marrone, D. Marinò

Policlinico Federico II (Naples, IT)

**Introduction** This case describes a 43-year-old woman with "type 3 Von Willebrand disease" and severe diffuse arthropathy that in the 2007 underwent left total knee arthroplasty. At age of 14, due to recurrent hemarthrosis, the patient underwent left knee synovectomy, the following year underwent right knee synovectomy and after five years underwent left elbow synovectomy.
**Materials and methods** The patient presented chronic left knee pain, ROM 5°–30°, double support walking, HSS Knee score 38. For the poor conditions of both bone-stock and ACL and collateral ligaments, we chose a constrained prosthesis. Factor VIII (FVIII) was administered during the operation and also in the following 4 weeks to further correct the plasmatic levels of these factors. Considering the high thromboembolic risk (related to surgery, reduction of mobility and the chronic use of estroprogestinic therapy), thrombotic prophylaxis was performed with low molecular weight heparin.
**Results** After 26 months the clinical and radiographic control showed a substantial improvement with pain reduction, ROM 0°–95°, HSS knee score 94 and perfect prosthesis integration.
**Discussion** Total knee arthroplasty in type 3 Von Willebrand disease patients (prevalence 1:1,000,000) is a complex surgery with high risk of hemorrhagic and infective complications.
**Conclusions** The possibility to improve quality of life in such a satisfying manner by surgery, let the patients do not take care of potential problems and complications related to it.

### ACL reconstruction and coagulation factor XI deficiency

V. Marcialis*, V. Verderosa, A. Manunta, A. Melis

AOU Sassari, Clinica Ortopedica (Sassari, IT)

**Introduction** We present a case of post traumatic anterior cruciate ligament lesion in a sixteen-year-old patient with closed physis which we diagnosed coagulation factor XI deficiency after surgery.
**Materials and methods** Ligament was reconstructed with four-strand semitendinosus and gracilis tendon using zip loop suspension system for femoral fixation and interference screw to achieve tibial fixation. Thromboembolic prophylaxis was administered as pharmacological protocol.

**Results** Post-operative great hemorrhagic extravasation occurred; after drains were removed (500 cc approx.) limb was swollen, edematous with important subcutaneous ecchymosis. The patient reported pain and weakness. Blood tests showed severe anemia (hemoglobin 5.7 g/dl) and coagulation profile showed factor XI deficiency states so heparinic therapy was suspended. In the following days clinical condition improved and passive motion of the limb began. Weight bearing was permitted as tolerated from day 5; the patients had to obtain an arc of at least 0° to 90° in 20 days with Kinetec passive motion. Thrombo embolic prophylactic therapy with Clexane 4000 UI was administered in consideration of high thrombo-embolic risk and the temporary resolution of hemorrhagic symptoms.
**Conclusions** Congenital deficiency of coagulation factor XI disease causes mild bleeding symptoms and it is often diagnosed after trauma or surgery. In patients with this kind of diseases is necessary to perform minimally invasive technique to reduce morbidity, swelling, soft tissue hematomas and simplify rehabilitation. Reconstruction with patellar tendon gives minor risk of blood loss than four-strand semitendinosus and gracilis tendon. Gravity drain is to prefer to vacuum type because it can reduce haemarthroses and retain into the joint cavity factors released by tunnels accelerating integration; constant monitoring is an essential tool managed in close collaboration with Blood Transfusion Centre related to clinical objectivity.

### Total knee replacement and non traumatic patellar tendon rupture in a patient with recurrent patellar dislocation

D. Pili*, M. Cossavella, P. Laura, A. Manunta

AOU (Sassari, IT)

**Introduction** More and more patients undergo TKR surgery because of the increasing average age, life expectancy and higher demand for functional capacity of the population. This has increased the cases of total knee replacement implants and therefore also the complications of this kind of surgery.
**Materials and methods** We report the case of a 71-year-old retired lady who underwent her first right TKR at 68 years of age. This lady was examined in our Department one week after noticing functional loss of knee extension of her right knee, pain over the insertion of the patellar tendon and the presence of an extensive bruising over the same area. X-rays of the right knee showed a well positioned total knee replacement. The ultrasound carried out on the area revealed a subtotal rupture of the patellar ligament. In the patient's medical history, suffering from malignant breast cancer, it was revealed that the patient was previously treated with tamoxifen and targeted radiotherapy one year after the operation. In addition this lady underwent a proximal quadriceps tendon tenoplasty at the age of 25 years and a transposition of the patellar tendon at the age of 66 years to avoid the recurrent dislocations. We treated this lady surgically with a full reconstruction of the extensor tendon with homologous calcanear tendon graft with resolution of pain and functional recovery.
**Results** Patients with a history of recurrent dislocation of the patella are at greater risk of tendon rupture of the patellar tendon. This risk increases in patients with TKR and in conjunction with other factors such as the use of tamoxifen.
**Discussion** We believe it is appropriate, in patients with a history of risk factors associated with tendon rupture, the study of the patellar tendon with MRI or ultrasound scan to identify patients with high risk of rupture prior undergoing a TKR.
**Conclusions** In cases with high risk for tendon rupture we advocate the use of biotechnologies or primary implant of auto-graft to reduce the risk of rupture.

### The treatment of knee cartilage defects: results with the AMIC technique

A. Schiavone Panni, S. Cerciello, M. Vasso, A. Palombi

Istituto di Clinica Ortopedia, Università del Molise (Campobasso, IT)

Surgical treatment of cartilage defects is the most debated aspect of orthopaedic practice since these lesions are relatively common in young and active patients. Unfortunately they influence the function of the whole joint and the may prejudice the career of a sportsman. Moreover they are responsible for fast cartilage degeneration even on the unaffected compartments. Several surgical options have been proposed in the past decades, varying from bone marrow stimulating techniques such as perforations and microfractures, to osteochondral and autologus chondrocyte transplantation. All these techniques have their own specific inconveniences and they recreate a fibrous cartilage and not the native hyaline one.
The AMIC (autologus matrix induced chondrogenesis) technique has been recently introduced. It consists in a porcine biocompatible and biodegradable collagen membrane which is called Chondrogide. This membrane has a two-layer structure with a porous deep part and a smooth superficial one. It is constituted by type 1 and 3 collagen which act as a scaffold for the penetration, growth and differentiation of chondral cells. To enhance cells flow from the bone marrow multiple perforation or microfractures are performed within the lesion. The membrane is then sealed to the margins of the lesion with fibrin glue to avoid blood dispersion. This technique is simple, it necessitate neither two-step surgery, nor donor site and recreates a hyaline like cartilage. The membrane must be protected in the first post-operative weeks, thus although mid range continuous passive motion is allowed, weight bearing is delayed for at least 4 weeks. Moreover we stress the necessity of strict patient selection. In case of knee cartilage defects we propose this procedure in patients with age varying from 18 to 50 years, with more than 2 $cm^2$ stage 3 or 4 defects according to the ICRS classifications, and in stable and well aligned knees. In case of kissing lesions this technique is contraindicated. Following these strict indications, from June 2007 to June 2009, 13 patients were treated with the AMIC technique in our Department. The lesion involved the medial condyle in 5 cases, the lateral in 2, the femoral trochlea in 4 and the patella in the last 2 cases. At the time of the last clinical follow-up, 9 patients were satisfied or very satisfied with their result, 3 were fairly satisfied and 1 was not satisfied. We believe the AMIC technique represent a reliable option in the treatment of chondral pathology in young and active patients. Nonetheless to achieve the expected satisfactory results strict indications must be followed.

### Revision of unicompartmental knee replacement

A. Schiavone Panni, M. Vasso, S. Cerciello

Istituto di Clinica Ortopedia, Università del Molise (Campobasso, IT)

Continue surgical technique and instrumentation improvement associated with materials and design evolution have increased effectiveness and popularity of UKR in the last 10 years.

Unicompartmental knee arthroplasty provides some advantages as less peri-operative morbidity and post-operative pain, less peri-operative bleeding, shorter hospital stay and inferior social costs. Polyethylene wear, loosening and deterioration of the unreplaced compartments remain the most important causes of failure in UKR. Careful patient selection and newer instrumentation has reduced the progression of arthrosis and tibial loosening leaving polyethylene wear as the predominant failure mechanism in contemporary UKR. Revision for polyethylene wear usually occurs after a minimum of 8 years, but some early catastrophic failures from wear have been reported. Excessive wear affects joint alignment and stability leading to an increased load at the bone-implant interface and accelerate loosening. Wear increases the debris volume at the bone-implant interface producing osteolysis which plays a role in component loosening (and unreplaced compartment degeneration). Factors associated with accelerated wear of the tibial polyethylene are polyethylene thickness, manufacturing processes, sterilization method, shelf aging, reduced conformity in the design. Polyethylene is now thicker than 6 mm for fixed bearings and the shelf age of polyethylene inserts sterilized with gamma irradiation in air is well documented. Congruous mobile-bearing implants could play a substantial role in reducing wear if they are not associated with dislocation and non-reproducible surgical technique. Recent improvements in manufacturing processes like cross-linking may be valuable in some fixed and mobile designs. Important technical factors include accurate and modern instrumentation avoiding component-to-component malposition and edge loading, allowing slight under-correction of the pre-operative deformity even with a minimally invasive incision. Conversion of the UKR to TKR may result technically demanding, especially in septic revisions. Challenges of UKR revisions may be joint exposure, component removal, bone defects and instability. Conversion of the failed UKR can be a technically demanding procedure that depends on how conservative the initial procedure was and the mode of failure. As the amount and success of UKR procedure increase we will continue encounter failures that require conversion to TKR.

### Unicompartmental knee replacement with ZUK prosthesis: mid-term results

A. Schiavone Panni, M. Vasso, S. Cerciello, A. Palombi

Istituto di Clinica Ortopedica, Università del Molise
(Campobasso, IT)

Differently from the historic clinical reports, all recent series in literature show excellent or good results at long-term follow-up after unicompartmental knee replacement. This difference is mainly the consequence of improved surgical technique and materials, and the new prosthetic designs and ancillary. Aim of our study was to report the mid-term results with ZUK prosthesis. Thus we performed a retrospective study on 80 patients treated for unicompartmental arthritis or necrosis. Maximum follow-up was 5 years.

From February 2005 to December 2007, 80 ZUK prosthesis were implanted in 80 patients affected by primary unicompartmental arthritis (68 patients) or necrosis (12 patients). Right and left knees were affected in 43 and 37 cases respectively. Medial and lateral compartments were involved in 71 and 9 patients respectively. The status of the ACL was not a strict indication criteria as well as patello-femoral cartilage aspect except for bone to bone damage. Mean age at the time of surgery was 69 years. In all cases a ZUK prosthesis, consisting in fixed bearing metal back tibial tray was implanted. All patients were evaluated with IKS scoring system and with complete X-ray study to check pre-operative and post-operative limb alignment and components positioning.

Survivorship rate at the last follow-up was 100%. Knee and function scores improved from 46 and 54 preoperatively to 80 and 94 at the lat follow-up. As expected, since this implant replace the loosened amount of cartilage and do not correct bony deformities, no variations in the lower limb alignment (hyper or hypo correction) was detected on postoperative X-ray controls.

Unicompartmental knee replacement joins the concepts of tissue sparing and minimally invasive surgery. This allows for lower peri-operative morbidity and faster recovery with shorter hospitalization and lower costs for health system if compared to total joint replacement.

### Technical notes on the implant of a total knee arthroplasty after tibial valgus osteotomy

A. Schiavone Panni, S. Cerciello, M. Vasso, A. Palombi

Istituto di Clinica Ortopedia, Università del Molise (Campobasso, IT)

High tibial osteotomies represent an historic chapter of orthopaedic practice. In the past decades they have been proposed in the treatment of constitutional varus deformity in young adult and arthritic varus knee. Recently they have been associated with ligament reconstruction and chondral defects treatment to change the lower limb axis and thus to protect them. The advantages of maintaining the propioception and the native knee make them the ideal choice in young and high demanding patients. Unfortunately several series in literature report deterioration of the initial good functional and X-rays results along with time. In such cases the only option relies on the implant of a total knee arthroplasty. This is a more technically demanding option if compared to a primary prosthesis. Thus an accurate preoperative planning as well as precise surgical techniques are mandatory. However some aspects must be considered in preoperative planning to adopt proper surgical solution at the time of surgery. Some problems are related to previous surgeries: surgical approach, joint exposure and hardware removal. These aspects may require a two-stage procedure and can be extremely challenging especially in case of patella baja. Soft tissue balancing can be demanding in case of concavity retraction and convexity slackness. Finally proximal tibia malunions could lead to loss of anatomical references and increased tibial slope thus making tibial cut difficult. Even in case of a precise surgical technique, several studies report lower functional and clinical results compared to primary implants. According to these considerations we do believe patient information to be crucial both at the time of osteotomy (explaining the possible deterioration of functional results) and at the time of further prosthesis (explaining the possible intra-operative complications and unpredictable post-operative functional results).

### *C56 – BASIC RESEARCH*

### Effect of local injection of platelet concetrate and microfrature on the treatment of focal chondral defects of the knee: an experimental study

L. Deriu*, M.F. Saccomanno, V. Izzo, F. Donati, G. Milano, C. Fabbriciani

Istituto di Clinica Ortopedica, Università Cattolica del Sacro Cuore
(Rome, IT)

**Introduction** Arthroscopic microfractures represent the mostly used surgical technique for the treatment of full-thickness chondral defects of the knee. It is well known that platelet-derived growth factors (GFs) can stimulate chondrogenic differentiation of BMSCs and enhance chondrocyte proliferation and metabolism. Autologous Conditioned plasma (ACP) is a blood-derived product with no leukocytes and a two-fold platelet concentration compared to whole blood. The purpose of the present study was to assess efficacy of repeated local injections of platelet concentrate on cartilage repair after microfractures for the treatment of full-thickness focal chondral defects of the knee.
**Materials and methods** A full-thickness chondral lesion on the weight-bearing area of the medial femoral condyle was performed in 30 sheep. Animals were randomly divided into 2 groups (15 animals each), according to treatment: in Group 1: Microfractures; Group 2: Microfracture + five weekly injections of ACP. Animals were sacrificed at 3, 6 and 12 months after treatment. Contralateral knees were used as controls. Histological evaluation was performed on each sample. Slices were stained with haematoxylin eosin (H-E) and Safranin-O/fast green, and examined under the light microscope. Histological appearance was scored according to a modified O'Driscoll score. Comparison between groups was performed with Mann–Whitney test. Significance was set at $p < 0.05$.
**Results** In group 1, repair tissue consisted of a thin layer of non-cartilaginous tissue. A great amount of small rounded cells covered the surface of the defect. Tidemark formation was almost absent. Healthy cartilage surrounding the defect showed clusters of chondrocytes along the border of the defect. In group 2 repair tissue showed a regular surface, flushing with that of healthy surrounding cartilage and covered almost completely the defect. However, it was thinner than surrounding cartilage. A great amount of small rounded cells showed a columnar arrangement similar to that of hyaline cartilage. Tidemark was present in the defect area. Group 1 showed a significantly worse histological score than group 2 at each time interval.
**Discussion** Local injections of ACP combined with a microfracture produce hyaline-like cartilage at 6 and 12 months after treatment.
**Conclusions** ACP showed a positive effect on the healing process of chondral lesions of the knee after microfractures.

### Perineurium and Endoneurium contribute two distinct patterns in the production of the "bands of Fontana" in the rat sciatic nerve

A. Merolli*, L. Mingarelli, S. Serpieri, F. Fanfani, F. Catalano

Università Cattolica (Rome, IT)

**Introduction** In 1781 the anatomist Felice Fontana identified the appearance of transversal and oblique bands (or "striae") along peripheral nerves. The gentle stretching the nerve produces an increase in the spacing of the spiral bands but, withdrawing the distracting force, a "recoiling" occurs similar to that occuring in a metallic spring. Since this early observation, several Authors reported on the possible structural basis of this morphological characteristic; the conclusions described the "bands of Fontana" as an optical illusion produced by the scattering of incident light on the assembly of wavy nerve components. Several papers identified staggered endoneural components as the cause, while others pointed at the contribution of perineural components.
**Materials and methods** We documented the recoiling phenomenon, in the rat sciatic nerve, by a 30 frames per second digital video recording, while performing in vivo microdissection of the nerve trying to single-out the causative components. With the aid of a Computer Assisted Design (CAD) software, we thus made a model of the nerve structural assembly trying to reproduce the visual appearance of the "bands of Fontana".
**Results** Well defined "spiral bands" were visible, in vivo, around the main fascicles of the nerve; however, they became extremely faint (and more "staggered" than "spiral") as tiny endoneurial streaks were dissected. Digital video recording showed that the spiral bands recoil back and forth, always with the same morphology, in fascicles with an intact Perineurium. In vivo microdissection showed distinctive black & white (greater amplitude), highly packed (shorter wave-length) bands in the Perineurium which are distinguishable from widely spaced (longer wave-length), translucent dark grey - pale grey (smaller amplitude), staggered bands in the Endoneurium. Images of "interference figures" produced by CAD, merging these two patterns of waving features, provided pictures resembling the appearance of the bands in vivo.
**Discussion** Our observations may provide a possible explanation which is able to incorporate some of the apparently conflicting evidences of the past.
**Conclusions** Two different repetitive structures, one with a shorter wave-length and a bigger amplitude, in the Perineurium, the other with longer wave-length and smaller amplitude, in the Endoneurium, can merge under incident highlight and give rise to the appearance of the "bands of Fontana".

### Rivaroxaban versus Enoxaparin Sodium in the prevention of venous thromboembolism in major orthopaedic surgery

S. Gagliardi, A. Macaione, F. Boniforti*

Fondazione San Raffaele Giglio (Cefalù, IT)

**Introduction** Patients undergoing major orthopaedic surgery have a high risk for thromboembolic events. The low molecular weight heparins (EPBM) are commonly used in post-operative prophylaxis, but there are other molecules that act by directly inhibiting factor Xa, and are administered orally, such as rivaroxaban.
**Materials and Methods** We studied sixty patients undergoing hip and knee prosthetic surgery, thirty-three received LMWH (Clexane 1FL 4000 IU sc daily with the first dose 12 h after surgery), twenty-seven 1CP Rivaroxaban 10 mg daily (with first dose 8/12 h after surgery), post-operative prophylaxis lasted 8 weeks for patients receiving EPBM and 6 weeks for patients receiving Rivaroxaban. Patients were evaluated clinically for deep vein thrombosis and pulmonary embolism post-operatively and 8 and 12 weeks after treatment.
**Results** There were no cases of pulmonary embolism and deep vein thrombosis from a total of sixty patients concerned.
In the Rivaroxaban group 11% (3 patients) had blood loss from the wound (more than 500 ml) and infarcitura of the surgical site that required discontinuation of therapy.
**Conclusions** The oral administration of the drug offers benefits on the tolerability and patient compliance, but in our experience there were episodes of bleeding that must be taken into account in assessing the effectiveness and the handling of the drug.

### One stage osteochondral repair with cartilage fragments in a hybrid scaffold: rabbit and goat animal model

A. Marmotti*[1], M. Bruzzone[1], F. Castoldi[1], R. Rossi[1], D.E. Bonasia[1], U. Cottino[1], A. Maiello[1], C. Bignardi[2]

[1]Università di Torino (Turin, IT);
[2]Politecnico di Torino (Turin, IT)

**Introduction** One-step techniques are a promising approach for cartilage repair. This study introduces a simple chondrocyte culture-free approach with autologous cartilage fragments loaded onto a mixed scaffold composed of high molecular weight hyaluronic acid derivative felt (Hyaff-11), injectable human fibrin glue (Tisseel) and autologous platelet-rich plasma (PRP).

**Materials and methods** *In vitro* "Rabbit-scaffold" preparation: Hyaff-11 was cut to pieces of 0.7 $cm^2$. Two sheets were piled in a culture dish and homogeneously hydrated with 100 ul of autologous PRP. 10–15 mg of cartilage fragments were evenly seeded atop the membrane and retained with a coating of approximately 200 ul of fibrin glue (thrombin-diluted to1:4). "Goat-scaffold"preparation: Hyaff-11 was trimmed into pieces of 0.7 $cm^2$. Two sheets were piled-up in a culture dish. 10–15 mg of cartilage fragments were evenly seeded on the membrane and retained with a coating of approximately 50 µl of fibrin glue (thrombin-diluted to1:4). 200 µl of Platelet-rich-fibrin-matrix (Cascade) was then placed on top of the cartilage fragments.

**Culture in standard growth medium** At 1 and 2 months: H/E-staining, Type-II-collagen-immunohistochemistry.

*In vivo rabbit*: one large-full-thickness-osteochondral-defect (4,5-mm-diameter,4-mm-depth) center of the trochlea of the right knee; defects randomly assigned to one of 5 different groups: (Group1) cartilage-fragments loaded onto the rabbit-scaffold with fibrin glue; (Group 2) cartilage-fragments loaded onto the rabbit-scaffold without fibrin glue; (Group 3) rabbit-scaffold alone with fibrin glue; (Group4) rabbit-scaffold alone without fibrin glue; (Group 5) empty-defects. Rabbits euthanized after 1 month (2 animals per group), 3 months (3 animals per group), 6 months (4 animals per group).

*Goat*: 2 large-full-thickness-osteochondral-defects (7-mm-diameter; 3.5-mm-depth) center of the trochlea of the right knee; defects randomly assigned to 3 different groups: (GroupA) cartilage fragments in goat-scaffold; (GroupB) goat-scaffold alone; (GroupC) empty-defects. Goats euthanized after 1 month (3 goats), 3 months (3 goats), 6 months (3 goats), 12 months (6 goats). All animal specimens: stained with Haematoxylin/Eosin, Alcian blue, immunoistochemistry for type-II-collagen. Nanoindentation performed on goat samples.

**Results** *In vitro* Chondrocyte outgrowth into the scaffolds was observed at 1 month; at 2 months neo-matrix positive for collagen-type-II was evident within the scaffold. *In vivo*: quality of repair tissue was better in presence of cartilage fragments (rabbit group 1 and 2, goat group A) than in control groups, albeit differences were more noticeable in the goat model because fibrin glue impaired repair process of rabbit defects. Nanoindentation of goat samples showed mechanical properties of group A close to native goat-trochelar-cartilage.

**Discussion** Autologous cartilage fragments loaded into a HA/PRP/fibrin glue scaffold are a viable cell source and improve the repair of osteochondral defects in rabbit and goat models.

**Conclusions** This approach shows an effective alternative for a simple non-culture-based-one-stage-repair of osteochondral defects. A clinical application seems promising, although further studies are needed to clarify the underlying mechanisms of this repair process.

### Biophysical stimulation with pulsed electromagnetic field of human SAOS-2 osteoblasts seeded onto trabecular wood (Luffa aegyptiaca) scaffolds

L. Fassina*[1], L. Visai[2], G. Magenes[1], F. Benazzo[3]

[1]Dipartimento di Informatica e Sistemistica, Università di Pavia (Pavia, IT);
[2]Dipartimento di Biochimica, Università di Pavia (Pavia, IT);
[3]Dipartimento SMEC, Università di Pavia (Pavia, IT)

**Introduction** A recent study [1] showed the potential of trabecular wood as biomaterial scaffold for orthopaedic applications. In order to extend the use of trabecular wood to the field of bone tissue engineering, we developed a new culture technique involving a pulsed electromagnetic field [2] (Igea Clinical Biophysics, Carpi, Italy) and an in vitro culture of human SAOS-2 osteoblasts seeded onto trabecular wood (Luffa aegyptiaca) scaffolds.

**Materials and methods** In particular, the pulsed electromagnetic field (magnetic field intensity = 2 mT; induced electric tension = 5 mV; frequency = 75 Hz; duration = 24 h/day) was applied to the trabecular wood for 22 days after the seeding of 1 million of osteoblasts/scaffold.

**Results** DNA extraction showed that the cell number grew to 2.40 millions in the control without biophysical stimulation, whereas to 3.91 millions in the electromagnetically stimulated wooden trabecular scaffolds ($p < 0.05$). The extraction of bone proteins (type-I collagen, decorin, type-III collagen, osteopontin, osteocalcin, osteonectin, fibronectin, and alkaline phosphatase) via a sample buffer and their quantitative evaluation via ELISA showed that the electromagnetic stimulation significantly enhanced the secretion and the deposition of bone matrix ($p < 0.05$). The SEM images showed that, in the control cultures, the cells were few and not surrounded by bone matrix, whereas the biophysical stimulation elicited the development of osteoblasts/bone matrix clusters tending to cover the surface of the trabecular wood.

**Discussion** All these observations reveal our "biomimetic strategy": onto the wooden biomaterial surface, the electromagnetic stimulation caused an enhanced formation of a new biocompatible surface composed of bone cells and their extracellular matrix.

**References**

1. Aho AJ, Rekola J, Matinlinna J et al. (2007) Natural composite of wood as replacement material for osteochondral bone defects. J Biomed Mater Res B 83:64–71.
2. Fassina L, Visai L, Benazzo F et al. (2006) Effects of electromagnetic stimulation on calcified matrix production by SAOS-2 cells over a polyurethane porous scaffold. Tissue Eng 12:1985–1999

### Vitamin MK-7 and vitamin D effects on proliferation and differentiation of human mesenchimal stem cells

S. Cecconi*, D. Enea, A. Busilacchi, S. Manzotti

Clinica Ortopedica, Università Politecnica delle Marche (Ancona, IT)

**Introduction** A significant proportion of clinical orthopaedic research involves attempts to stimulate bone healing thanks to substances with osteoinductive properties. A considerable amount of data indicates that vitamin D and vitamin K are essential to maintain proper homeostasis of bone metabolism. The aim of the present study is to assess the effects on differentiation and proliferation of vitamin MK-7 alone and in combination with vitamin D on human mesenchymal stem cells (hMSC).

**Materials and methods** Proliferation of hMSC cultured with the two types of vitamins alone and in combination was evaluated at 3, 10 and 20 days by MTT method. A differentiation of hMSC in osteoblastic sense was assessed at 10 and 20 days by analysis of gene expression and intracellular (Flow Cytometer) and extracellular (ELISA) presence of osteogenic proteins such as osteocalcin. As controls untreated hMSC were used.
**Results** As for the MTT analysis there is a greater proliferation in cells treated with the combination of both vitamins. The gene expression showed activation of genes typically for osteoblastic cell line in those cells treated with vitamin D alone or in combination with MK-7. The osteocalcin expression, mainly intracellularly, increased in cell groups treated with vitamin D alone or in combination compared with the control group, while having an increase of its carboxylated form in the group treated with vitamin MK-7 alone or in association.
**Discussion** Results showed that the combination of the two vitamins seem to have a positive influence on both proliferation and differentiation in osteoblastic sense of human mesenchymal stemm cells. This effect appears to be due mainly to the presence of vitamin D, while the combination with vitamin MK-7 promotes the production of the active form of osteocalcin, a protein essential for bone ingrowth.
**Conclusions** The literature has so far placed the focus on the role that these vitamins can have in the treatment of osteoporosis based on their role on bone metabolism. The results of our study suggest that association of vitamin MK-7 and D can promote or at least accelerate the process of reparative osteogenesis, as in delayed union or non-union, thanks to their osteoinductive properties.

### Bone regeneration from adipose tissue: experimental study

A. Pozzuoli*[1], S. Tamburin[1], B. Zavan[2], G. Abatangelo[2], R. Aldegheri[1]

[1]Clinica Ortopedica, Università di Padova (Padua, IT);
[2]Dipartimento di Istologia, Microbiologia e Biotecnologie Mediche, Università di Padova (Padua, IT)

**Introduction** An innovative approach for the treatment of bone loss is represented by tissue engineering based on the ability of some cells to differentiate in vitro in osteogenic sense when seeded on adequate biomaterial scaffolds and in presence of osteogenic medium. This capacity is proper of human adipose derived stem cells (hADSCs) which are multipotent, easily accessible, and abundant. However, some studies hypothesize the involvement of stem cells in the oncogenic process. The aim of this study was to investigate the osteogenic differentiation potential of hADSCs seeded on 3 different biomaterials and the safety of this procedure evaluating possible chromosome abnormalities which could compromise the use of this method.
**Materials and methods** ADSCs were isolated from lipoaspirate by enzymatic digestion and morphologically characterized researching stem cell markers by immunofluorescence. Then, cells were seeded on three scaffolds (one osteoconductive x and two demineralized bone matrices y1 and y2) in presence of osteogenic medium and tested for cell proliferation by vitality assay. Osteogenic differentiation was evaluated analyzing gene expression of bone matrix proteins by real-time RT–PCR. The research of structural alterations in the genome was done with CGH-array.
**Results** The study is still in progress; the results obtained till now are referred to the osteoconductive scaffold. The stem cells markers are positive. The vitality assay shows that cells proliferate on biomaterials. Real time RT-PCR confirms that seeded cells express osteogenic markers. None chromosome abnormalities were present in all the cultures at every passages.
**Discussion** hADSCs seeded on osteoconductive scaffolds have positive stem markers confirming that mesenchymal stem cells have been obtained. The vitality test confirms that cells proliferate and good amplify on the biomaterial that is, therefore, not cytotoxic. Moreover, hADSCs express osteogenic markers showing that correct phenotype has been acquired. Absence of chromosomic alterations guarantees the safety of biomaterial and of stem cells.
**Conclusions** The results show that hADSCs adhere, proliferate, and differentiate in osteoblast-like cells if seeded, in presence of osteogenic medium, onto an osteoconductive scaffold that is, therefore, biocompatible. Moreover, chromosomic analyses confirm the safety of the cultures. The results obtained till now seem to support the use of hADSCs as source of mesenchymal stem cells for bone tissue engineering.

### Chitosan porous biomimetic scaffolds for tissue engineering

A. Di Martino*[1], L. Liverani[2], A. Rainer[2], F. Abbruzzese[2], G. Salvatore[1], M. Trombetta[2], V. Denaro[1]

[1]Cattedra di Ortopedia, Università Campus Bio-Medico di Roma (Rome, IT);
[2]Laboratorio di Ingegneria Tissutale, Università Campus Bio-Medico di Roma (Rome, IT)

**Introduction** Tissue engineering represents an innovative approach to for tissue regeneration and repair, and it is based on the development of scaffolds that aim to promote adhesion, proliferation and differentiation of non-differentiated cellular populations. It is possible to fabricate these biomimetic scaffolds by adding specific macromolecules usually found in non-pathologic native tissues. In this work the chosen biomaterial for scaffold fabrication is chitosan, a natural polymer widely employed in several tissue engineering applications in the orthopaedic field.
**Materials and methods** We used a chitosan solution (medium molecular weight, Sigma–Aldrich) at 2% w/v in an aqueous solution of 2% v/v lactic acid (Sigma–Aldrich). The selected reticulant agent was genepine (Sigma–Aldrich). Samples were lyophilized at -20°C for 48 h. Through the same technique we also performed other functionalized samples adding hydroxiapatite (HA, Sigma–Aldrich) in the polymeric solution. Scaffolds were seeded with adult human mesenchymal stem cells and kept in culture both with undifferentiating (aMEM) and osteogenetic and chondrogeneic differentiating soil (positive control) for 3 weeks. The scaffolds, both the seeded and the unseeded ones (negative control), were submitted to morphological, mechanical and chemical analysis. Scaffolds were also submitted to test of citotoxicity (G6PDH, Invitrogen) and cellular proliferation (MTT assay, Sigma–Aldrich) as well as to, at the arrival of the culture time point, cell differentiation (IF and IHC with specific antibodies) in order to evaluate the influence of HA.
**Results** Samples with interconnected pores that have an average diameter of 20 micrometers were obtained. Addition of HA did not modify the scaffold morphology, but improved its mechanical properties. The citotoxicity and proliferation tests did not show significant differences compared to controls. Positive results were obtained for both osteogenic and chondrogenic differentiation.
**Discussion** The addition of HA in order to give the scaffold biomimetic characteristics improves its physical, chemical and mechanical properties, thus making the scaffold bioactive, and favouring both osteogeneic and chondrogeneic differentiation.
**Conclusions** Biomimetic scaffolds were fabricated with interconnected pores that showed good performance in both osteogenic and chondrogeneic tissue regeneration. The addition of other growth factors chemically bound to the scaffold is under evaluation.

### ROUND TABLE

### *PHYSICAL THERAPY AND REHABILITATION*

### Oxymetry evaluation of local perfusion reduction after shock wave in the treatment of tendinopathy

B. Moretti[1], A. Notarnicola*[1], L. Moretti[1], M. Forcignanò[1], M. Maiorano[1], S. Tafuri[2], V. Pesce[1]

[1]U.O. Ortopedia e Traumatologia, Policlinico di Bari (Bari, IT);
[2]Dipartimento di Igiene, Università degli Studi A. Moro di Bari (Bari, IT)

**Introduction** Some authors have recently demonstrated the prevalence of angiogenesis in the shoulder affected by rotator cuff tendinosis as compared to the contralateral unaffected shoulder. In view of this finding, Alfredson and Ohberg suggested treating tendinosis by sclerosing procedures, reporting a significant regression of the clinical and anatomopathological picture after such treatment. Aim of this study was to assess the effect of extracorporeal shock wave therapy (ESWT) on tissue perfusion in the treatment of tendinopathy.
**Materials and methods** An observational clinical study was made on 30 patients undergoing ESWT for tendinopathy of the rotator cuff. ESWT was administered with a Minilith SL1 (Storz, Swiss) electromagnetic generator equipped with in-line US-guidance, during 3 sessions, at 3–4 day intervals, emitting 2000 impulses with an energy flux density (EFD) ranging from 0.04 to 0.07 $mJ/mm^2$. Before each session, and at each follow up (FU), we recorded the tissue oxymetry values of the symptomatic and contralateral shoulders, using the cerebral and somatic/peripheral oxymeter INVOS 5100C (Somanetics Corporation, USA).
**Results** A clinical improvement was obtained in 65.6% of patients at 2 and 6 months. This was associated with a statistically significant reduction in the oxygen tissue saturation, measured by oxymetry, that was apparent already during treatment, as well as at subsequent follow-up visits.
**Discussion** It has been hypothesized, on the basis of results in experimental studies, that shock waves applied to the tendon tissue increase VEGF synthesis, inducing new vessel formation, increased transforming growth factor-beta1 and insuline-like growth factor-I expression and fibroblasts, stimulating the tenocyte metabolism and extracellular matrix deposits, as well as inhibiting the synthesis of interleukin-1 and metalloproteinases, thus limiting the inflammatory process. In view of our results, we can hypothesize that in an inflamed tendon (even in a degenerative process) where the angiogenic process is active, ESWT induces an integrated anti-inflammatory and regenerative action on the different tissue components. The progressive resolution of the inflammation process then limits and reduces the primary local angiogenic response and thus modulates the disease process and restores the metabolic and functional balance of the various tendon structures.
**Conclusions** The reduced perfusion achieved with ESWT supports the hypothesis that this treatment can regulate the inflammatory process and offset increased vascularization, restoring physiological tendon conditions.

### Importance of rehabilitation in the outcome of fragility fracture patients

I. Cerocchi*[1], N. La Terza[2], G. Cannata[1], M. Celi[1], M. Feola[1], U. Tarantino[1]

[1]UOC Ortopedia e Traumatologia, Fondazione PTV, Università degli Studi Tor Vergata (Rome, IT);
[2]Università degli Studi Tor Vergata (Rome, IT)

**Introduction** Several elements must be considered when creating a proper rehabilitation program in fragility fracture patients, aimed at obtaining global recovery, not ignoring the consequences of osteoporosis. Aim of the study was to evacuate the effect of different rehabilitation approaches on short- and long-term functional regain after a hip fracture.
**Materials and methods** Study population included 40 women (average age 67.5 years, range 60–75 years), 20 with a medial fracture of the proximal femur treated with hip arthroplasty, and 20 with a lateral fracture fixed by intramedullary nailing. Risk factors for osteoporosis were examined and DXA of the lumbar spine (L1–L4) and proximal femur was performed within 72 h from intervention, and after 6 and 12 months. The "Tinetti Balance and Gait Scale" and the "Short Form-36" (SF36) were also used, and the results were related to the rehabilitation received.
**Results** Seven patients (17.5%) only performed activities aimed at regaining articularity, muscle tone and ambulation (passive/assisted active movements, muscle strengthening exercises), and massage therapy. The others also followed a school for ambulation with walking aids, obstacle course at parallels and stairs. Only 3 women received water rehabilitation. SF-36 scores decreased after fracture and showed further reduction at 1 years, probably due to loss of positive effects related to rehabilitation, which was interrupted nor replaced by alternative physical activities. General SF-36 score was significantly higher in patients who performed at least three activities. Even the Tinetti Balance and Gait Scale values were lower, except for women who had followed a more complete rehabilitation program. In subjects who had received water rehabilitation we didn't observe any worsening at 12 months, and the score remained 28/28.
**Discussion** In our study population patients who had a more complete rehabilitation program showed better quality of life and physical performance. Furthermore, in most cases we observed worse conditions after discontinuing rehabilitation.
**Conclusions** Full recovery of ROM should not neglect strengthening of all muscular chains, proprioception, ambulation school, balance rehabilitation, pain reduction, water rehabilitation.

### Observational studies on scoliosis in the province of Cosenza: the role of rehabilitation

L. Scalzo*[1], F. Burchi[1], B. Scornaienchi[1], A. Bucciero[2], S. Caracciolo[3]

[1](Piano Lago, IT);
[2](Rome, IT);
[3](Cosenza, IT)

**Introduction** Scoliosis is a disorder characterized by derlla spine tilt of the spine and rotation of the vertebrae. Epidemiologically in recent times has increased the spread of school age.
**Materials and methods** Our work is based on clinical and functional evaluations simple and complex in 5 schools in the province of Cosenza. The survey provides, in addition to the medical testing posture, the creation of working groups based on global postural re-education techniques or other types of posture.
**Results** The study is still in progress. The preliminary data are certainly insufficient to allow a statistical treatment. Anyway there is a clear correlation between scoliosis and some parameters of life (sex, sport, date of first menstruation). In some cases, scoliosis is part of a broader global postural dysfunction configuring a tonic postural system dysfunction.
**Discussion** Scoliosis is a condition extremely common in school age. Scoliosis is often in close connection with TMJ dysfunction or disruption of the visual system or the vestibular system.
**Conclusions** In this area a close collaboration between physiotherapists, physical therapists, orthopaedists, who graduated in physical education is fundamental.

### Rehabilitation pathway in hip and knee prostheses

M. Celestini*, G. Graziani, M.L. Nicodemo

UOC Medicina Fisica e Riabilitazione, Ospedale S. Spirito (Rome, IT)

Is the individual rehabilitation project in patient over 70 surgically treated with prosthetic hip or knee replacement always necessary?
In recent months, the ASP of Lazio has given indications of post-acute rehabilitation of patients surgically treated with prosthetic hip or knee replacement, supporting the indication of rehabilitation at home and/or possible admission to extensive rehabilitation.
We believe the need for comprehensive care of this type of patient, with a recovery program schema motor and step performed in a specialized environment, to prevent early and late complications (DVT, wound infection, dislocation, generalized pain, gait deficit, etc.), and to give the clinical stabilization of vital parameters (oxygenation, metabolic and hemodynamic balance) in relation to the complexity of care related to multiple comorbidities.
Therefore we believe a short stay in intensive rehabilitation (2 weeks) to be the most proper and safe procedure which was also directed to the proper recovery of the autonomy of the person and probably to a reduction of health costs.
